# Dimensions of Migrant Integration in Western Europe

**DOI:** 10.3389/fsoc.2021.510987

**Published:** 2021-04-29

**Authors:** Anthony F. Heath, Silke L. Schneider

**Affiliations:** ^1^ Centre for Social Investigation, Nuffield College, University of Oxford, Oxford, United Kingdom; ^2^ Department Survey Design and Methodology, GESIS – Leibniz Institute for the Social Sciences, Mannheim, Germany

**Keywords:** migrant integration, assimilation, Western Europe, structural integration, social integration, cultural integration, political integration, civic integration

## Abstract

The integration of immigrant minorities is a major concern for diverse societies–with major implications for the well-being of those affected, social cohesion and group relations, and economic and social progress. In this paper, we give a comprehensive description of long-term migrant integration in Western Europe to investigate theories of migrant assimilation and integration. We take a multidimensional approach, looking at 10 indicators measuring social, structural, political, civic and cultural integration. We take an innovative approach to measuring minority background by using two complementary measures: generational status, distinguishing first and second-generation migrants from the third and higher up ‘natives,’ and self-reported ancestry, separating those with autochthonous-only ancestry from those with various kinds of allochthonous ancestry. Using interaction effects between these measures, we can test whether generational change is faster or slower for some ethnic groups than for others, i.e. whether different groups integrate at differing speeds. Using the pooled samples of all Western European countries included in the European Social Survey rounds 7 and 8, we run multivariate regression analyses to estimate the effects of migrant background on the 10 indicators of integration. Compared to migrants with autochthonous ancestry, respondents of Middle Eastern, North African & Central Asian as well as Sub-Saharan African ancestry are less integrated on all dimensions of integration except the political and civic ones. The South & South-East Asian group is also substantially less assimilated socially and culturally, but not so much structurally. They are closely followed by the South East and East European groups, following the same pattern except that the latter are less integrated politically as well. We only find substantial interaction effects between ethnic group and migrant generation for two integration indicators, namely citizenship and homophobia, for which speed of integration thus appears to differ across ethnic groups. For all other indicators, integration speed does not appear to differ across ethnic groups, supporting straight line assimilation theory, with social integration in terms of interethnic friendship potentially rather following a ‘bumpy-line’ pattern.

## INTRODUCTION: Migration and Integration

With increasing ethnic diversity as a result of recent decades of immigration in all Western European countries, the integration of ethnic minorities with a migration background has become a major concern of national governments, policymakers, academics, the wider public, and the individuals directly affected themselves. These concerns cover a wide range of issues. The minorities themselves are concerned about the inequality and entrenched disadvantage that they experience in the labor market, as shown by the high rates of unemployment and low-skilled work experienced by migrants and the children of migrants (see for example OECD and European Union, 2015). Governments have been concerned about the possibility that ethnic minorities will come to live parallel lives in segregated communities and in consequence fail to adopt mainstream values and ways of behavior (Casey, 2016) increasing the risks of ‘homegrown’ radicalism and conflict. Academics have debated the drivers of lack of integration, exploring the roles of the characteristics of the migrants themselves (Koopmans, 2010), the impact of government policies such as multiculturalism (Wright and Bloemraad, 2012; Bloemraad and Wright, 2014), and of racisms and discriminatory treatment of minorities on the part of the majority group in the country of destination (Portes and Zhou, 1993; Reitz, 1998; Heath et al., 2013; Ramos et al., 2020).

While there has been considerable previous research on the integration of migrants in individual countries and some comparative work on specific dimensions (van Tubergen et al., 2004; Marks, 2005b; van Tubergen and Kalmijn, 2005; Levels et al., 2008; Kristen et al., 2011; OECD and European Union, 2015), there has been little systematic work using a multidimensional approach and distinguishing the major ethnic groupings (but see Crul et al., 2012). Although in a local context it may be clear who these immigrant minorities are, in wider debates very different groups are commonly treated as if they were one homogeneous group, or simply disaggregated by generational status only (Marks, 2005a; Marks, 2005b; Schleicher, 2006). As our results will demonstrate, such approaches hide more than they reveal. This over-simplification is also not likely to foster migrant integration.

The aim of this paper is therefore to describe immigrant integration on a wide range of indicators and examine how different ethnic groups as well as different migrant generations fare compared to the autochthonous population. Using pooled European Social Survey (ESS) data, we explore the integration of migrants and their descendants across eight Western European countries. We take a multidimensional approach to integration and compare the extent of integration along social, structural, cultural, political and civic lines. Exploring the integration of 11 broad ‘pan-ethnic’ groups (such as West Europeans, East Europeans, East Asians, Latin Americans) compared to the autochthonous majority, we test central theories of integration such as classic ‘straight line’ assimilation theories of generational distance from immigration, and more recent theories of segmented assimilation.

In the next section, we briefly introduce the concept and theories of immigrant integration. Section *Data, Measures and Methods* introduces the survey data used in the empirical analyses and the measures of minority background, presents approaches to the distinction of various dimensions of integration and their measurement, as well as the analysis strategy. Then we present the results, including a number of robustness checks. The last section summarizes and discusses the results and points to avenues for future research.

## The Concept and Theories of Immigrant Integration

There is no single settled definition of the concept of integration. One complication is that the term integration tends to be used more often by European scholars than by Americans, who tend to prefer the term assimilation, but there is a great deal of overlap between the two concepts. Early treatments of the concept of assimilation in the USA, developed in the context of migration from Europe in the early decades of the 20th century, tended to focus on processes of acculturation – “incorporation … in a common cultural life” as Park and Burgess (1921, p. 735) described it. In contrast, post-war European writing on ethnic integration (perhaps influenced by European theories of social class inequality) tended to focus on issues of the socio-economic inclusion and exclusion of post-war labor migrants from less developed countries such as Turkey (e.g., Castles & Kosack, 1973). However more recent American work, such as that of Gans (1992) on second-generation decline and Portes and Zhou’s (1993) theory of segmented assimilation incorporate the socioeconomic side into a broadened concept of assimilation, while European scholars have increasingly debated socio-cultural aspects of integration (Koopmans, 2016). We shall thus treat the two concepts as effectively synonyms. The core of both concepts is that individuals and groups become fully part of a wider whole, the latter usually thought of as the nation-state in which the individuals reside.

As one might expect, a variety of different theoretical frameworks for understanding migrant integration have been suggested. First, the classic ‘straight line’ theory of assimilation, based on the experience of European migrants to the United States, in essence put forward a generational approach to assimilation, arguing that the first generation of immigrants would be the least assimilated, retaining many of the values, identities and modes of behavior of their countries of origin (Gans, 1979; Warner and Srole, 1945). As Warner and Srole describe the process, based on their pre-war (1930–35) research on eight European-origin groups in Yankee City, “The ethnic generation born abroad and migrant to this country is the one attached most strongly to the ancestral social system, and its derivative, the ethnic community in Yankee City, and least to the Yankee City social system … The offspring of these immigrants, the “filial first” or the “F^1^” generation, having been born, reared and schooled in the United States, know nothing of the ancestral society of their parents except as it is partially represented in the ethnic group’s community organization. The members of the F^1^ generation acquire wider external relations with the Yankee City society than their parents and bring more elements of American culture into their internal group relations. The children of the F^1^ generation, whom we label F^2^, and the children of the F^2^ generation, whom we label F^3^, exhibit similar progressive shifts in social personality” (Warner and Srole, 1945, p. 30).

In effect, the legacy of the country of origin was expected to weaken the more distant it became in terms of generations. Later generations, Warner and Srole argued, “have ceased participating in the ethnic life of their ancestors and have disappeared in the larger American world” (p. 2). According to this account, generations are the motor of ethnic change, not simply the time frame (Alba and Nee, 1997, p. 832).

While Warner and Srole did not themselves use the term ‘straight line’ assimilation, their over-time data on the eight European-origin groups do approximate to straight lines moving steadily upwards (albeit at different speeds), although subsequent scholars such as Gans (1992) have suggested that ‘bumpy line’ might be a more apt description of the trends over generations. At any rate, in their study Warner and Srole (1945) described the way in which the eight groups each entered Yankee City at the lowest residential, occupational and social class locations but then moved gradually up over time and generations (the two being broadly equated). Warner and Srole concluded that “It seems likely that oncoming generations of new ethnics will go through the same metamorphosis and climb to the same heights that generations of earlier groups have achieved” (Warner and Srole, 1945, p. 2).

Warner and Srole do, however, qualify this broad prediction with two important caveats. First, they argued that the greater the difference between the host (American) and the immigrant cultures, the greater will be “the subordination, the greater the strength of the ethnic social systems, and the longer the period necessary for the assimilation of the ethnic group” (p. 285). Second, they argued that the process of assimilation might be even slower and more painful for racial minorities because of the “force of American organized “prejudice” against the dark-skinned people” (p. 294).

The scholarly consensus is that “assimilation has been the master trend among the descendants of the immigrants of the previous era of mass immigration, who mainly came from Europe in the period before 1930” (Alba and Nee, 1997, p. 841). A major concern in the subsequent literature is whether the picture of straight-line assimilation for European-origin groups drawn by Warner and Srole will apply to the ‘new’ immigration to Europe and America of the postwar period (see Gans, 1979 for a summary of critiques of straight-line theory). One important issue, as Alba and Nee (1997) and Waters (2014) have pointed out, is that a generational theory of change may have fitted the early 20th century American context better than it does contemporary contexts, where there is continuing migration from many origin countries. For example, there have been continuing inflows of new migrants from Mexico to the US, from Turkey to Germany, or from Pakistan to Britain leading to ‘replenishment’ of the ethnic community and potentially keeping alive the culture and traditions of the origin country (Waters, 2014). This is an important difference from the mid 20th century American situation where major restrictions on immigration were introduced after the first world war and effectively lasted until 1965.

Another important dissimilarity between early 20th century America and contemporary contexts is that the account of straight-line assimilation theory applied to the migration of European migrants from culturally similar Christian countries such as Ireland, Poland and Italy in contrast to the migration of labor migrants and refugees from non-European and often non-Christian countries in the postwar period. A number of scholars have suggested that straight line assimilation might not apply in the same way to these non-white and/or non-Christian groups as it had applied (and might still apply) to groups of European heritage.

There are two main variants on this argument, both of which had in fact been anticipated by Warner and Srole. The first emphasizes enduring processes of exclusion and discrimination on the part of the majority group against non-white or non-Christian groups, leading to downward mobility and socio-economic marginalization of stigmatized minorities (Gans, 1992; Portes and Zhou, 1993). Systematic reviews of field experiments suggest that racial discrimination in the labor market persists against non-white groups both in Europe and America and against the second generation as well as the first, thus inhibiting structural integration (Zschirnt and Ruedin, 2016; Quillian et al., 2019). These exclusionary processes in turn, it has been suggested, might lead to ‘reactive ethnicity’ on the part of marginalized ethnic groups, working in the opposite direction to cultural assimilation and serving to maintain distinct ethnic identities (Warner and Srole, 1945; Rumbaut, 2008, p. 284). Along somewhat similar lines, other scholars have focused on the differing ‘warmth of the welcome’ and contexts of reception facing different contemporary migrant groups and their implications for integration (Portes and Zhou, 1993; Reitz, 1998; Crul et al., 2012).

A second variant, which has been applied particularly by European scholars to Muslim minorities in Europe, has emphasized the role of Islam, or at least of some traditional attitudes associated with Islamic countries of origin, in impeding integration both structurally and socio-culturally (Sniderman and Hagendoorn, 2007; Koopmans, 2016; Zuccotti and Platt, 2017). This variant emphasizes the way in which high levels of religiosity and traditional attitudes associated with Islam, especially attitudes to gender roles, might impede the structural integration of Muslim women as well as limiting social mixing and intermarriage across ethnic lines. These values might in turn be preserved across generations by strong religious communities around the Mosque.

Another major difference of the contemporary 21st century context from the earlier context is that of growing income inequality in developed countries, especially in the United States and United Kingdom but also in many other Western and North European countries such as Sweden, Germany and the Netherlands (Atkinson et al., 2017). There have been declining opportunities for disadvantaged members of the majority group to achieve upward mobility, and hence there are potentially greater risks of entrenched disadvantage for minorities than there were in previous eras.

Theories of segmented and selective assimilation (Portes and Zhou, 1993; Zhou, 1997) provide an integrated account of these different theoretical ideas, focusing on the way in which different groups may follow different paths on different dimensions, depending on the extent of discrimination and exclusion they are subject to, the economic opportunities open to them, and the strength of the ethnic community and its social and economic capital. As Portes and Zhou write, “One of [these paths] replicates the time-honored portrayal of growing acculturation and parallel integration into the white middle class; a second leads straight in the opposite direction to permanent poverty and assimilation into the underclass; still a third associates rapid economic advancement with deliberate preservation of the immigrant community’s values and tight solidarity” (1993, p. 82).

Portes and Zhou’s examples show how Black migrant groups might experience downward mobility, while simultaneously assimilating socially with lower class African American youth, and how some Asian groups experience upward (structural) mobility while maintaining their ethnic communities. For the European context, one might want to extend Portes and Zhou’s account to include the potential role of religious communities and their institutions such as the gurdwara, temple or mosque. While Christian communities may provide a potential bridge with the mainstream and thus more rapid social and cultural assimilation, non-Christian religions can potentially maintain the separate existence of ethno-religious communities through their separate religious institutions, thus enhancing bonding rather than bridging social capital (Connor and Koenig, 2013).

Segmented assimilation theory therefore suggests that different ethnic groups may experience different intergenerational trajectories and that rates of assimilation may vary across different dimensions as well. Whereas Warner and Srole’s (1945) classic treatment had assumed that assimilation on residential, occupational and social class lines (in the form of upward social mobility) would also be accompanied by and indeed facilitate assimilation with respect to social behavior and cultural attitudes, Portes and Zhou’s (1993) segmented approach allows for discrepancies in the extent of assimilation along different criteria, with the pattern of upward across-the-board assimilation being only one among several possibilities.

Scholars have raised some doubts about Portes and Zhou’s (1993) account, particularly about the extent to which downwards mobility has actually occurred among the children of migrants in the USA. While there is evidence that many of the first generation – the migrants themselves – experience downwards mobility, especially at the early stages of their lives in a new country, downward mobility for the second generation does not appear to be a widespread phenomenon (e.g., Perlmann and Waldinger, 1997). As Portes and Zhou emphasized, the pattern of downward assimilation was contingent on the prior existence of a substantial and highly disadvantaged African American underclass in the United States, a phenomenon that has no direct parallel in any European country.

The central aim of this paper, therefore, is to explore in the European context questions about the integration of different migrant groups, and in particular to compare disparities across ethnic groups as well as migrant generations. Specifically, we will compare the integration of groups of European heritages, and thus relatively close culturally and ethnically to the majority groups in Western Europe, with visible minority groups such as black and non-Christian (especially Muslim) groups from origins outside Europe.

In the light of the American debates over straight-line, bumpy line and segmented assimilation accounts, we address three main research questions about integration and assimilation in the European context. Firstly, we ask which broad ‘pan-ethnic’ groups, in the contemporary European context, show patterns of increasing integration across generations along the lines of ‘the time-honored portrayal of growing acculturation and parallel integration into the white middle class’ (Portes and Zhou, 1993, p. 82). Specifically, is this pattern predominant among groups with a Western (i.e., European, North American or Australian) heritage but less frequent among non-European groups from more culturally distant origins? Secondly, do black groups stand out as being likely to experience entrenched disadvantage and downward assimilation across generations, perhaps as a result of persistent racism? Thirdly, do we find uneven patterns of change across the different dimensions of integration along the lines of Portes and Zhou’s third path of ‘rapid economic advancement with deliberate preservation of the immigrant community’s values and tight solidarity,’ that is to say with intergenerational progress on the socio-economic dimension while socio-cultural differentiation is preserved across generations?

## Data, Measures and Methods

### Data

We use the European Social Survey (ESS) rounds 7 and 8, collected in 2014/15 and 2016/17 respectively (European Social Survey, 2014a; European Social Survey, 2016), to examine migrant integration in Western Europe. The ESS is a biannual, cross-sectional face-to-face probability sample survey of attitudes, beliefs and behaviors conducted in over 36 European countries since 2001 (for an introduction to the ESS, see Schnaudt, et al., 2014). It covers individuals from the age of 15, with no upper age limit.^1^ The analyses reported in this paper are based on the pooled ESS samples in order to obtain sufficient numbers of respondents from various socio-cultural origins. We only use the data from the eight Western European countries included in the ESS: Austria, Belgium, France, Germany, Ireland, the Netherlands, Switzerland and the United Kingdom.^2^ Table 1 gives an overview of the country samples in ESS rounds 7 and 8, and Table 2 presents descriptive statistics on the control variables education, sex and age in the pooled sample.

**TABLE 1:** Sample sizes per ESS round and participating Western European country.

Country	ESS round		Total
	7	8	
Austria	1,795	2,010	3,805
Belgium	1,769	1,766	3,535
France	1,917	2,070	3,987
Germany	3,045	2,852	5,897
Ireland	2,390	2,757	5,147
Netherlands	1,917	1,681	3,598
Switzerland	1,532	1,525	3,057
United Kingdom	2,264	1,959	4,223
Total	16,629	16,620	33,249

Source, ESS round 7, ed. 2.2 and ESS round 8, ed. 2.1.

Notes, Data not weighted.

**TABLE 2:** Descriptive statistics for control variables.

		Round 7			Round 8			Total	
**Education**		***n***	**%**		***n***	**%**		***n***	**%**
Low: Less than upper secondary		4,539	27.56		4,025	24.39		8,564	25.98
Medium: Upper secondary		5,722	34.75		5,942	36.01		11,664	35.38
Post: Post-secondary not high		2,576	15.64		2,540	15.39		5,116	15.52
High: Higher education		3,631	22.05		3,995	24.21		7,626	23.13
Total		16,468	100.00		16,502	100.00		32,970	100.00
**Sex**									
Male		7,957	47.85		8,013	48.21		15,970	48.03
Female		8.672	52.15		8,607	51.79		17,279	51.97
Total		16.629	100.00		16,620	100.00		33,249	100.00
**Age**	**min**	**max**	**mean**	**min**	**max**	**mean**	**min**	**max**	**mean**
	15	102	49.61	15	100	49.81	15	102	49.71

Source, ESS round 7, ed. 2.2 and ESS round 8, ed. 2.1.

Notes, Data not weighted.

### Measures of Immigrant Background

In contrast to many studies, we measure both specific ethnic and cultural origins, as well as the generational status of migrants in Europe. The specific measures are described here in turn, followed by a presentation of their co-occurrence. Table 3 shows the joint distribution of both measures.

**TABLE 3:** Joint distribution of socio-cultural origin and generational status.

	Generational status			
	3rd gen and majority	2nd gen	1st gen	Total
0 Only autochthonous ancestry	24,371	1,537	298	26,206
11 West European	657	474	674	1,805
12 North European	9	12	24	45
13 South European	334	452	353	1,139
14 South-East European	32	196	390	618
15 East European	107	129	599	835
80 North American & Australasian	18	42	71	131
20 MENA & Central Asian	40	425	488	953
30 Sub-Saharan African	10	39	202	251
40 South & South-East Asian	33	156	243	432
50 East Asian	8	20	50	78
60 Latin American	7	38	116	161
70 Caribbean	53	68	61	182
Total	25,679	3,588	3,569	100

Source, ESS round 7, ed. 2.2 and ESS round 8, ed. 2.1.

Notes, Data not weighted.

#### Ethnic and Cultural Origins

In order to measure specific socio-cultural origins, we use the ancestry measure which was developed for the immigration module in ESS round 7. The respective questionnaire item asks the respondent which ethnic or cultural group (s)he considers him- or herself to descend from. It uses country-specific response options, presented to respondents on a showcard, which are recoded into the European Standard Classification of Cultural and Ethnic Groups (ESCEG, Heath and Schneider, 2018) after data collection. Respondents can indicate up to two ancestries. This measure focuses on ethnic and cultural *origins* rather than current identity because of the problem of ‘ethnic identity leakage’ - the fact that higher generation respondents, especially those of mixed ancestry, may not identify with the origin of their ancestors any more (see e.g., Waters, 2014) - and potential bias if current identity is used (for further details on this measure, see Heath et al., 2016; Schneider and Heath, 2019).

We use the broad groups of the ESCEG, i.e. the first digit of the classification, for all respondents reporting a non-European ancestry. The first digit broadly corresponds to what have been termed ‘pan-ethnic’ groups (Lopez and Espiritu, 1990). For respondents reporting a European ancestry, we differentiate the second digit of the classification (see left-most column in Table 3), since this group is large enough to merit closer inspection, and differences in the integration of migrants from different European origins in Western European destination countries are likely. Respondents are coded into the ‘only autochthonous ancestry’ group if they do not report any foreign ancestry.^3^

The traditional approach to measuring ethnic origins of respondents with a migration background has been to construct measures based on the respondents’ and their parents’ countries of birth (COB)–measures which are often available in large-scale datasets and which can readily be harmonized. The ESS also collects information on respondents’ and their parents’ country of birth. We construct a measure equivalent to the one mentioned above derived from measures of ancestry using country of birth information in order to give our results a robustness check. Although we would in general expect these COB measures to yield similar results to ours, one important problem with COB measures is that they are normally unable to identify third or higher generation respondents, who are likely to become increasingly numerous in Europe in the future. The measure of ancestry does not have any such limitation. It can therefore also be considered more ‘futureproof’ as a survey instrument for identifying ethnic minorities stemming from earlier migration waves than just those covering the current first and second generations. A second important problem is that COB measures are at risk of misclassifying ‘returnees’ – respondents who have autochthonous ancestry but who were born abroad, for example as a result of colonialism or religious persecution in earlier periods. Well-known examples are the French “*pieds noirs*,” Portuguese “*retornados*,” and returning members of the German diaspora in the former Soviet Union and those displaced from formerly German territories after WWII. In a measure based on COB, these will be classified as ethnic minorities although they rather belong to the majority ethnic group.

#### Generational Status

In the case of generational status, we distinguish three categories: First generation, second generation, and third and higher generations (i.e., mostly respondents without a recent migration background). All respondents who were born abroad and immigrated after age 5 (i.e., after primary education started in most countries) are regarded as first-generation immigrants, irrespective of where their parents were born. All native-born respondents with one or both parents born abroad, or foreign-born respondents who immigrated before age 6 to at least one foreign-born parent, are regarded as second generation i.e., the direct offspring of immigrants. Finally, respondents who were born in the country where the survey was held and whose parents were also born in this country, are regarded as 3rd and higher generation. While this group mostly consists of people without any migration background, it will also include respondents whose grandparents or more remote ancestors were migrants. Foreign-born respondents who immigrated before age 6 to native-born parents are also included in this last group.

To be sure, generational status and socio-cultural origin are related (see Table 3, Cramér’s V = 0.55). The large majority of respondents (74%) does not have a migration background and reports autochthonous ancestry only. Still, this means that a quarter of the population in the countries included in the analyses either has an observable migration background by belonging to the first or second generation, or reports a foreign ancestry (and mostly both). The smallest cell sizes are the Northern European, East Asian and Latin American third generation with less than 10 respondents each. We will not interpret the results referring to these combinations of backgrounds. Interestingly, non-ignorable numbers of respondents with first- or second-generation migration background report exclusively autochthonous ancestry - which would not be visible if using a COB-based measure of ethnic group (see above). We inspected the data very closely for a number of countries and concluded that while this may be counterintuitive at first glance, the numbers are very plausible in light of the history of migration and return migration in these countries (see Supplementary Material Appendix B).

### Integration Measures

In order to obtain a broad picture of immigrant integration in Europe, we measure integration in five distinct (although correlated) domains. Even within these broad domains, we aim to measure diverse aspects of integration, which is why we do not construct summary indices of e.g., structural integration or cultural assimilation but rather look at different socio-economic outcomes and different kinds of attitudes which may not correlate highly with one another.

Currently, there is no agreement among scholars either on the number of dimensions, or on their specific components. Jonsson and his colleagues (following Esser, 2006), for example, distinguish three dimensions – structural, cultural and social. They see the structural dimension as primarily capturing aspects of the vertical, hierarchical segmentation of society with respect to economic resources and positions, while the cultural dimension – the extent to which minorities and majority share knowledge, attitudes and values - and the social dimension – the extent of social ties between minority and majority groups - capture horizontal aspects of integration (Jonsson et al., 2018). Other scholars however have identified somewhat different dimensions or have grouped the sub-dimensions in different ways. Lessard-Phillips (2017), for example, on the basis of factor analysis of British data, empirically identified four dimensions - spatial, socio-economic, political, and cultural. Other scholars and institutions (e.g., OECD Union, European, 2015) have produced further different lists. Given the current state of knowledge, we do not believe that there is at present any one correct way to identify dimensions and sub-dimensions of migrant integration. In this paper, we distinguish five dimensions, adding a political dimension and a new ‘civic’ dimension to the three identified by Jonsson and colleagues.

#### Structural Integration

Structural integration is typically thought of as achieving parity with the major group in terms of economic resources and occupational positions. For structural integration, therefore, we look at the position of the individual in the labor market and household income. Thereby we do not only look at the situation of the respondent him/herself, but also at the wider household context. Consequently, we construct two structural integration indicators.

Firstly, we look at the socio-economic positioning of the individual in society based on their employment situation and occupation. More specifically, we examine whether there is a higher risk to be in a marginalized labor market position for people with an ethnic minority background. We speak about a marginalized labor market position if the respondent is either unemployed (variables *uempla* and *uempli*) or working in a low-skilled occupation, following Heath and Zwysen (2018), who looked at the economic integration of the second generation in Europe. Respondents who are economically inactive (e.g., in education, military service or retired) are excluded from this analysis. 25% of economically active respondents in the pooled ESS sample are either unemployed or work in low-skilled occupations. The variable is inverted so that negative coefficients signify ethnic minority disadvantages.

Secondly, the family’s material resources are operationalized by the total net household income from all sources (i.e., ignoring assets and wealth, for which we do not have any measures in the data). In the ESS, this is measured using country-specific income deciles. We use these deciles directly as our indicator (variable *hinctnta*). As usual, the income measure suffers from a high degree of item nonresponse (16%). The results regarding this indicator thus need to be treated with some caution. However, all deciles are populated with eight to twelve percent, so the distribution looks plausible, even though the bottom and two top deciles are somewhat smaller than the other deciles. The median is 5, as it should be.

#### Cultural Integration

Cultural integration is generally taken to involve the sharing of attitudes and values, although it could be broadened to include shared language (sometimes referred to as acculturation). Fluency in the destination-country language is a major influence on migrants’ structural integration (Dustmann, 1994), and probably on social integration, too (Chiswick and Houseworth, 2011). Unfortunately, however, the ESS does not include measures of fluency in the destination-country language(s) and so we focus instead on two specific values which are believed to differ markedly between immigrant and native populations in liberal Western European democracies (while recognizing that there is considerable diversity of opinion even within the majority group). The first one regards homophobia, where the ESS asks for agreement with the statement “Gay men and lesbians should be free to live their own life as they wish” (variable *freehms*) on a five-point agree-disagree scale. Higher values on this scale denote more homophobic attitudes, and we invert the measure. The mean for the West-European ESS sub-sample is then 4.3. The second value we look at is gender equality, measured by agreement with the statement “When jobs are scarce, men should have more right to a job than women” (variable *mnrgtjbrs*), using the same scale but where higher values mean more egalitarian attitudes. The cross-country mean here is then 4.2. This latter item is only available for ESS round 8.

#### Social Integration

Social integration – social mixing on equal terms between members of minority and majority groups – has been a key component of all conceptualizations from Park and Burgess (1921) onwards. This dimension is probably the most difficult to operationalize using ESS data, which for example does not measure partners' ethnic background. We have to make do with the following two proxies that are only available for ESS round 7 and thus entail a much smaller sample. Firstly, ESS round 7 asked respondents whether they have "any close friends who are of a different race or ethnic group from most [country] people?" (variable *dfegcf*). We code all as 1 who indicate No. So, we do not know whether respondents mix with natives, but only whether they do or do not mix with ethnic minority members. We however assume that those who mix more with minority members may mix less with the majority. We find that 56% of the sample report to have no close ethnic minority friends.

We can secondly use the question on the respondent’s area of residence (variable *acetalv*) and regard as more socially segregated those (minority) respondents reporting to live in an area where many people are of a different race or ethnic group from that of the majority group. 16% of respondents in the Western European ESS sub-sample report that they live in such an area. Those 84% living in an area with no or only some ethnic minority groups are coded 1.

#### Political Integration

While political integration does not figure regularly in accounts of integration and assimilation, we regard it as a valuable aspect of integration into the public life of a nation. We distinguish two indicators of increasing constraint: non-electoral political participation, which is open to everybody; and having voted in the last national election, only open to citizens of voting age. These two indicators both reflect the intention to influence the development of the destination country as the migrant’s new home and are straightforward to measure using ESS data. Non-electoral political participation is measured in the ESS with a sequence of items (variables *contplt, wrkprty, wrkorg, badge, sgnptit, pbldmn, bctprd*) asking e.g., whether the respondent signed a petition or took part in a public demonstration in the last 12 months. From these variables, we constructed a binary variable scoring 1 if the respondent said “yes” for any of these items. 53% of the Western European ESS sub-sample show political participation of one form or another.

Secondly, for electoral participation, the ESS asks: “Some people don’t vote nowadays for one reason or another. Did you vote in the last [country] national election in [month/year]?” (variable *vote*). We coded as 1 all those saying ‘yes’ and coded to missing all those reporting not to be eligible to vote as well as non-citizens (who often apparently did not use the ‘not eligible to vote’-option). 80% of citizens eligible to vote reported that they had voted in the last national election in the pooled ESS sample.^4^

#### Civic Integration

A key component of Gordon’s canonical account of assimilation was ‘identificational’ assimilation which he saw as the development of “a sense of peoplehood based exclusively on [the] host society” (Gordon, 1964, p. 74). The criterion of exclusivity has been challenged as unrealistic in practice, and we accordingly drop it, but the concept of peoplehood seems to us a valuable one, and one which is distinct from the other components that we have covered so far. We term the dimension ‘civic’. We use two indicators, one subjective and one objective.

The subjective one refers to national identification, which comes very close to Gordon’s concept of a sense of peoplehood based on the host society. While there are no identical measures for ESS rounds 7 and 8, there are two measures that can be regarded as indicators for the same theoretical concept. In round 7, there is a question with a four-point scale asking, “How close do you feel to [country]?” with 1 “Very close” to 4 “Not close at all” (variable *fclcntr*). In round 8, there is a question with an 11-point scale asking “How emotionally attached do you feel to [country]? Please choose a number from 0 to 10, where 0 means not at all emotionally attached and 10 means very emotionally attached” (variables *atchctr*). After harmonizing scale direction and inspecting the individual distributions – both highly skewed towards feeling very close/very attached –, we constructed a combined measure using simple standardization.

The objective indicator refers to being a citizen, which can be considered the ultimate sign of successful integration from the destination state’s point of view. Whether the respondent has the country’s citizenship was directly asked in the ESS. 92% of the Western European ESS sub-sample report to have the citizenship of the country in which they were interviewed.

### Analysis Strategy

We run multiple regression analyses on all indicators of integration on the pooled ESS round 7 and 8 sample. We first run what we term the ‘main effects’ model in which the integration indicators are regressed on ethnic origin and generational status (plus controls). We follow this with models which also include interactions between ethnic origin and generational status, in order to test whether generational differences are smaller (or larger) among some ethnic groups compared to the autochthonous population.

We use linear regression for all integration indicators - including the binary ones. Because of the issues over comparison of effect sizes in the case of logistic regression models, and in order to keep an already complex model computationally as simple as possible, we thus report the results of linear probability models (LPMs) for binary variables, which has become quite acceptable again in recent years (see e.g., Breen et al., 2018). We also run the corresponding logistic regression models as a robustness check. To ease interpretation, we present adjusted predictions at representative values (APRs) for the full set of combinations of ethnic origins and migrant generations (see Williams, 2012), complemented by standard regression tables.

The regression models are identical for all indicators i.e., we use the same measures of minority background and generational status, and the same control variables. We include only a basic set of control variables, namely, age, gender and level of education in four categories (less than upper secondary, upper secondary - including vocational, post-secondary but not higher education, higher education). No age restrictions are applied to the sample. In the results tables, only the minority background and generation effects as well as interactions between these two variables are shown (but full results are available as Supplementary Material Appendix A).

We use country fixed effects to control for country level heterogeneity. We cannot use multilevel models, because we only have a low number of countries in our pooled sample and we are not looking at country level effects, which would anyway be problematic with these data (see Möhring, 2012).

For socio-cultural origin, we use the autochthonous as the reference category in order to be able to interpret all ethnic group coefficients as deviations from the majority population. We use the ‘first generation’ as the reference category on the generational status variable, so that we can see whether the second generation and the third and higher generations are more integrated than the first generation. We should also note that the number of third-generation respondents in some ethnic groups is very small, making the third generation an inappropriate reference category when examining interaction effects.

Following ESS recommendations, the data are weighted by the design weight provided in the ESS data files to correct for unequal selection probabilities due to the complex sampling design applied in a number of ESS countries (European Social Survey, 2014b). The design weight was combined with purpose-built weights equalizing sample sizes across countries, adjusted as per dependent variable (i.e., when analyzing those eligible to vote only, the baseline case number was reduced accordingly for each country). Thereby countries with larger samples get the same weight in the analyses as countries with smaller samples, rather than using the population size weights provided with the ESS data, which lead to a stronger influence of larger countries in the data.

## Results

First, we compare the model fit of ethnic minority integration models without and with interaction between ethnic group and generational status. This provides a global test of the hypothesis that different ethnic groups integrate at different speeds.

Table 4 shows the model fit measures for the main effects and interaction effects models across all indicators. With one important exception (citizenship), BIC gives strong evidence against models with interaction effects. AIC in contrast gives preference to the interaction effects models in all cases but one - interethnic friendships. The adjusted R^2^s only change very little when adding interaction effects, again with the exception of the citizenship indicator. It is clear, then, that we need to take account of the interaction effects in the case of citizenship, while it is also clear that interaction effects can safely be ignored in the case of the friendship indicator. In between, the three criteria of BIC, AIC and change in adjusted R^2^ suggest similar orderings, with interaction effects being more important in the case of the homophobia indicator and rather less important with the other indicators. In substantive terms this means that while we find equal generational effects across ethnic groups for most integration indicators, for a few indicators, speed of integration across migrant generations differs across ethnic groups. We will discuss this in further detail for those specific indicators.

**TABLE 4:** Comparing model fit of models without and with interaction terms.

Ethnic minority integration		Main effects model			Interaction effects model			Model comparison		
**Domain**	**Indicator**	**adj. R^2^**	**BIC**	**AIC**	**adj. R^2^**	**BIC**	**AIC**	**Δ adj. R^2^**	**Δ adj. BIC**	**Δ AIC**
Structural	No low-skilled job/unemployment	0.1378	30,721	30,496	0.1389	30,906	30,482	0.001	185	−14
	Net household income	0.1901	128,586	128,364	0.1911	128,772	128,352	0.001	186	−12
Cultural	Not homophobic	0.1354	70,211	69,985	0.1383	70,326	69,899	0.003	115	−86
	Gender equality	0.1212	41,515	41,307	0.1237	41,677	41,284	0.003	162	−23
Social	No or some migrants in neighborhood	0.0611	13,215	13,008	0.0640	13,374	12,982	0.003	159	−26
	No minority ethnic friends	0.1366	21,022	20,815	0.1370	21,223	20,830	0.000	201	15
Political	Voting	0.1172	25,401	25,178	0.1183	25,588	25,167	0.001	181	−11
	Political participation	0.0858	44,318	44,092	0.0871	44,498	44,070	0.001	180	−22
Civic	Citizenship	0.4849	−16211	−16,437	0.5174	−18,104	−18,532	0.033	−1,893	−2095
	National attachment	0.0964	88,164	87,938	0.0975	88,348	87,920	0.001	184	−18

### Structural Integration

**FIGURE 1:**
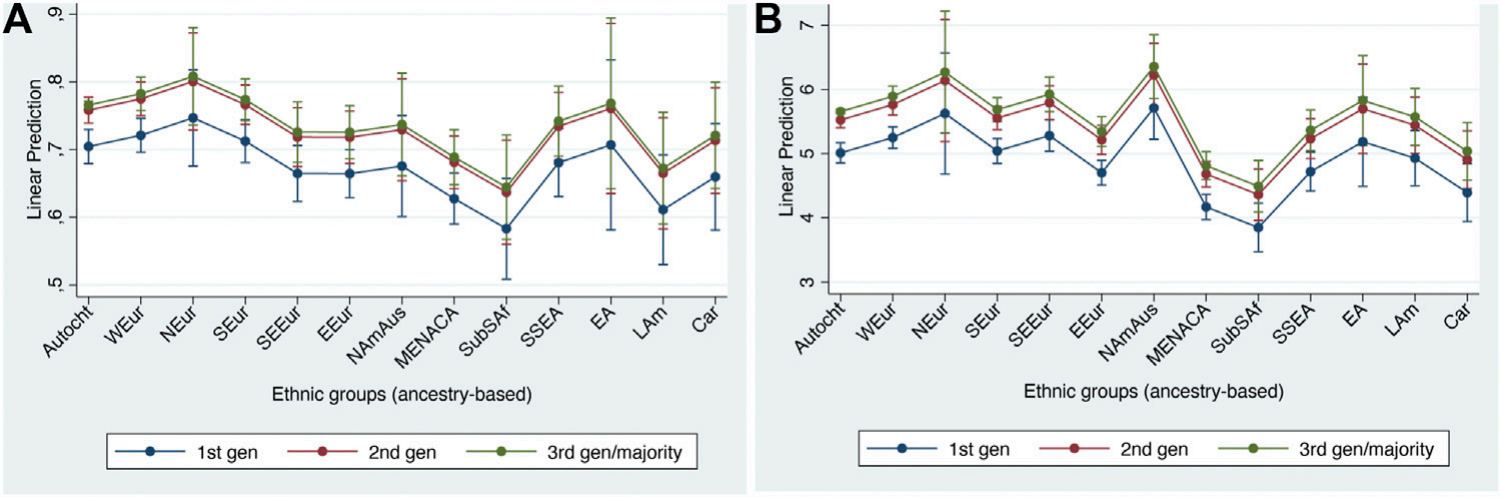
Adjusted predictions at representative values for structural integration indicators with 95% confidence intervals. **(A)** no marginal labor market position; **(B)** household income percentiles.

Table 5 shows the regression results of the ‘main effects’ model when predicting our structural integration indicators while Figure 1 shows the average predictions at representative values (APRs), derived from this main effects model, for both indicators.

**TABLE 5:** Regression models for structural integration (linear main effects models).

	No marginal labor market position (LPM, raw effects)			Household total net income percentiles (OLS, raw effects)		
	b		SE	b		SE
***Ancestry***						
Only autochthonous ancestry	Ref			Ref		
West European	0.02		0.01	0.24	**	0.08
North European	0.04		0.04	0.61		0.48
South European	0.01		0.02	0.03		0.10
South-East European	−0.04		0.02	0.27	*	0.14
East European	−0.04	*	0.02	−0.31	**	0.12
North American & Australasian	−0.03		0.04	0.70	**	0.25
MENA & Central Asian	−0.08	***	0.02	−0.84	***	0.11
Sub-Saharan African	−0.12	**	0.04	−1.17	***	0.21
South & South-East Asian	−0.02		0.03	−0.29		0.16
East Asian	0		0.06	0.17		0.36
Latin American	−0.09	*	0.04	−0.08		0.23
Caribbean	−0.04		0.04	−0.62	**	0.23
***Generational status***						
1st generation	Ref			Ref		
2nd generation	0.05	***	0.01	0.51	***	0.08
3rd gen and majority	0.06	***	0.01	0.64	***	0.08
***N***	30,259			27,843		

*p < 0.05, **p < 0.01, ***p < 0.001.

As we can see, the patterns shown by the two indicators are very similar, both with respect to the ethnic and to the generational differences. Generational status shows a highly similar pattern on the two structural indicators. The second generation is significantly less disadvantaged than the first, with only a slight further improvement among the third and higher generations. With respect to the ethnic differences, we find a considerable range between the twelve groups, ranging from substantial (but not always statistically significant) advantages for the West European, Nordic and North American & Australasian groups to substantial disadvantages for the Middle East, North African & Central Asian, and Sub-Saharan African groups. East Europeans are also significantly disadvantaged on both indicators, although to a lesser extent. Among the other groups, there are significant (dis)advantages on one or other indicator for respondents of Latin American origin (who are more often in a marginalized labor market position) and for the Caribbean group (who have substantial income disadvantages). First-generation South-East Europeans are as advantaged on the income indicator as West Europeans. Only the South European, the South & South-East Asian, and the East Asian groups are neither advantaged nor disadvantaged, relative to the autochthonous majority group, on either indicator.

Generational effects are weaker than the strongest of the origin effects, suggesting that some structural disadvantage remains even among second and third generation minorities of the most disadvantaged groups. In contrast, the East European disadvantages on both indicators appear to be restricted to the first generation and are cancelled out in the second generation, and the Caribbean disadvantage on income disappears with the third generation.

Even though the model with interaction effects between generation and ethnic group has already been shown to have worse model fit than the main effects model, supporting the hypotheses of equal speed of integration across ethnic groups, out of theoretical interest we briefly report on the significant interaction effects, to check whether we find any evidence for the hypothesis of downwards assimilation on the part of particularly disadvantaged, especially black, groups (see Supplementary Material Appendix A). There were in fact few significant interactions for either indicator, and when interactions were significant their signs were contrary to the hypothesis of downward assimilation for stigmatized groups. While this is not conclusive evidence, it is certainly out of line with the downwards assimilation hypothesis. Overall, then, our interpretation of these results for structural integration is that they are more in line with straight-line theory than they are with the hypothesis of downward assimilation.

### Cultural Integration

We turn to cultural integration next as many of the basic patterns are rather similar to those for structural integration. The two different indicators of cultural integration also show very similar patterns to each other, both for ethnicity and generation, although there are fewer significant effects in the gender attitudes model, probably because here the analysis sample is only half the size, with ESS round 8 available only.

**FIGURE 2:**
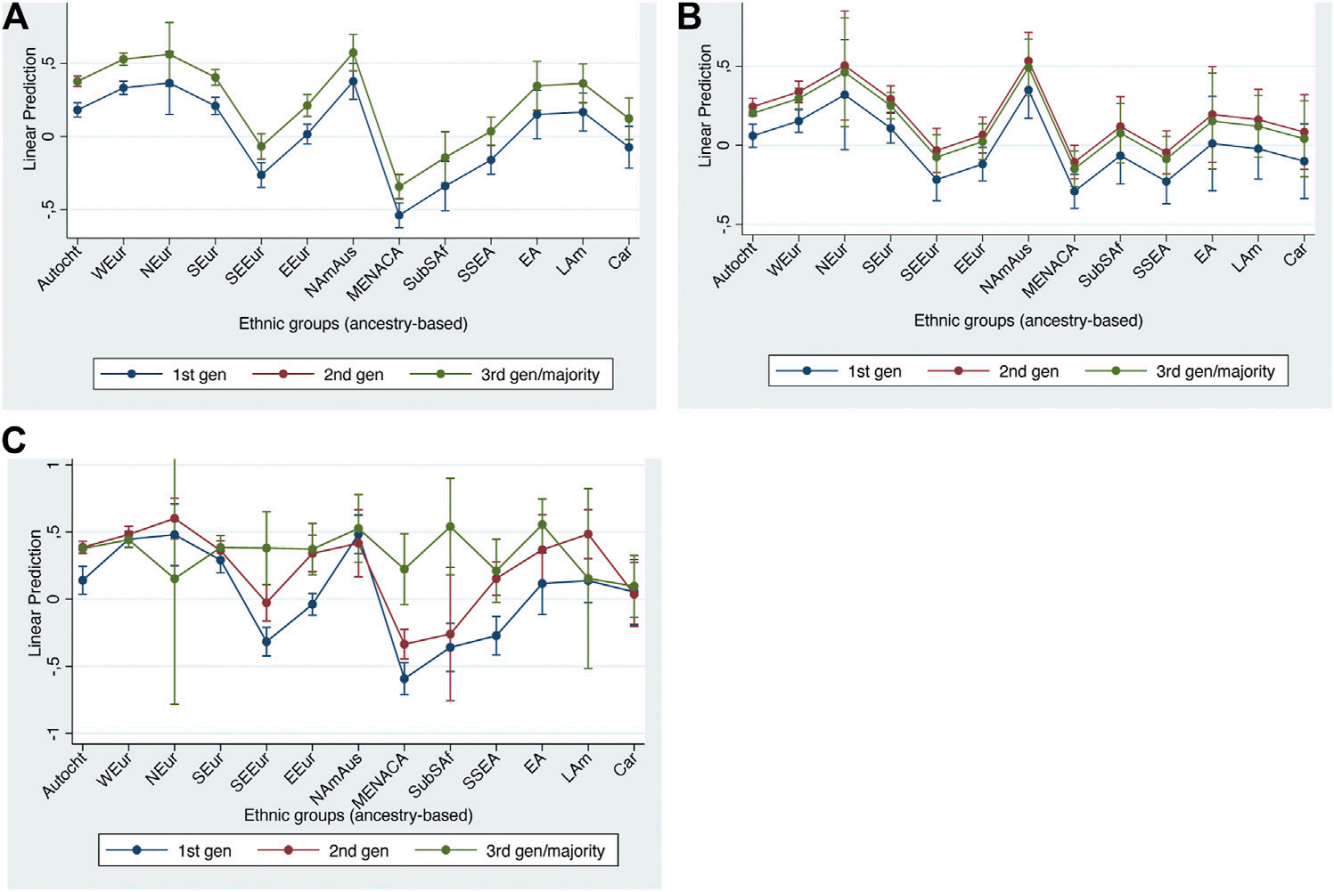
Adjusted predictions at representative values for cultural integration indicators with 95% confidence intervals. **(A)** non-homophobic attitude (main effects model); **(B)** gender egalitarianism (main effects model); **(C)** non-homophobic attitude (interaction effects model).

Looking first at Table 6 and Figures 2A,B, showing the APRs based on the main effects model, we find consistent generational effects, with more liberal attitudes amongst the second and third generations than in the first generation. Regarding ethnic group effects, we find a range of positive and negative effects, along similar lines to those for structural integration. Thus, among the West European, Nordic and North American & Australasian groups we find more liberal attitudes than among the autochthonous. In contrast there are consistently more conservative attitudes to be found amongst those of Middle Eastern, North African & Central Asian, South & South-East Asian, East, and South-East European as well as Sub-Saharan African (on one indicator) background.

**TABLE 6:** Regression models for cultural integration (gender equality in ESS8 only).

	Non-homophobic attitude (OLS raw effects)						Gender equality (OLS raw effects)		
	Main effects model			Interaction effects model			Main effects model		
	b		SE	b		SE	b		SE
***Ancestry***									
Only autochthonous ancestry	Ref			Ref			Ref		
West European	0.15	***	0.02	0.31	***	0.06	0.09	**	0.03
North European	0.18		0.11	0.34	**	0.13	0.26		0.18
South European	0.03		0.03	0.15	*	0.07	0.05		0.04
South-East European	−0.45	***	0.05	−0.46	***	0.08	−0.28	***	0.07
East European	−0.17	***	0.04	−0.18	**	0.07	−0.18	**	0.06
North American & Australasian	0.20	**	0.06	0.34	***	0.09	0.29	**	0.09
MENA & Central Asian	−0.72	***	0.04	−0.73	***	0.08	−0.35	***	0.06
Sub-Saharan African	−0.52	***	0.09	−0.50	***	0.11	−0.13		0.10
South & South-East Asian	−0.34	***	0.05	−0.41	***	0.09	−0.29	***	0.07
East Asian	−0.03		0.09	−0.02		0.13	−0.05		0.15
Latin American	−0.01		0.07	0.00		0.1	−0.08		0.1
Caribbean	−0.26	***	0.07	−0.09		0.13	−0.16		0.12
***Generational status***									
1st generation	Ref			Ref			Ref		
2nd generation	0.20	***	0.02	0.25	***	0.06	0.18	***	0.04
3rd gen and up	0.20	***	0.02	0.24	***	0.05	0.14	***	0.04
***Interaction effects*** (***only effects based on n ≥ 10 shown***)									
WEur # 2nd gen				−0.21	**	0.07			
WEur # 3rd gen+				−0.24	***	0.07			
NEur # 2nd gen				−0.12		0.15			
SEur # 2nd gen				−0.18	*	0.08			
SEur # 3rd gen+				−0.14		0.09			
SEEur # 2nd gen				0.04		0.1			
SEEur # 3rd gen+				0.46	**	0.16			
EEur # 2nd gen				0.13		0.1			
EEur # 3rd gen+				0.17		0.12			
NAmAus # 2nd gen				−0.31	*	0.16			
NAmAus # 3rd gen+				−0.19		0.16			
MENACA # 2nd gen				0.01		0.1			
MENACA # 3rd gen+				0.58	***	0.16			
SubSAf # 2nd gen				−0.15		0.28			
SubSAf # 3rd gen+				0.66	**	0.21			
SSEA # 2nd gen				0.18		0.11			
SSEA # 3rd gen+				0.25		0.15			
EA # 2nd gen				0.01		0.19			
LAm # 2nd gen				0.1		0.14			
Car # 2nd gen				−0.26		0.18			
Car # 3rd gen+				−0.19		0.18			
***N***	32,116			32,116			16,223		

*p < 0.05, **p < 0.01, ***p < 0.001.

As we noted above, there are indications that the interactions between ethnicity and generation may be important in the case of the homophobia indicator. Looking at Figure 2C, what we find when we add interaction terms is that there are both significant negative and positive interaction effects with a general pattern of regression towards the mean, i.e. assimilation to the destination country culture, in all groups. Among the West Europeans for example we find significant negative interactions for the second and third generations, cancelling out the generational main effects. In contrast, we find some significant positive interactions for the third generation (not second generation) among the Middle Eastern, North African & Central Asian, Sub-Saharan African, and South-East European groups. All groups converge with the majority in the second or third generation. This leaves no third-generation groups which are significantly less liberal than their autochthonous peers. The interaction effect between ethnic group and generation thus leads to equal attitudinal outcomes across ethnic groups despite the highly different starting points of the first generation in different ethnic groups.

In summary, there is wide dispersion among the first generation in their attitudes related to homosexuality and gender equality, with West European and North American being significantly more liberal, and East European, South-East European, Middle Eastern, North African & Central Asian, Sub-Saharan African, and South & South-East Asian being significantly less liberal. These latter differences are mitigated somewhat in the second generation with convergence in the third generation in the case of homophobia. The same could not be observed for gender equality, possibly because the sample for this indicator is too small to detect interaction effects.

### Social Integration

Patterns for social integration are quite similar to those that we have already seen for structural and cultural integration, although there are a few important differences, too. The two indicators also exhibit rather similar patterns as each other with respect to ethnic differences but differ with respect to generation.

**FIGURE 3:**
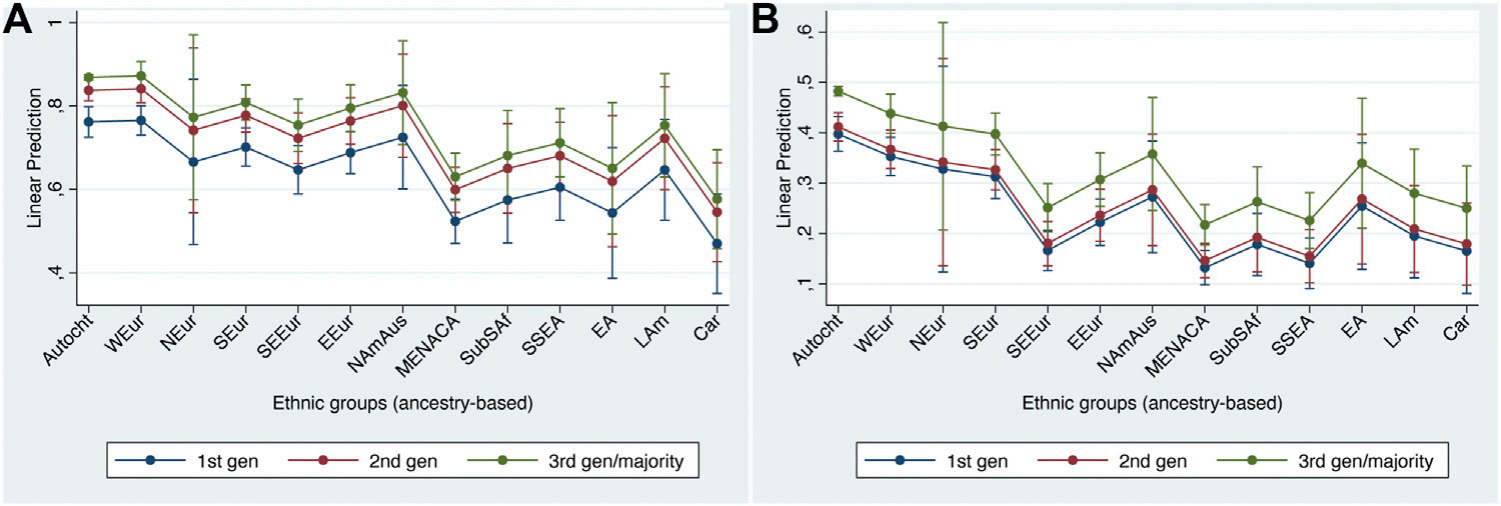
Adjusted predictions at representative values for social integration indicators with 95% confidence intervals (main effects models). **(A)** no or some minority ethnic minority people in living area; **(B)** no ethnic minority friends.

With respect to ethnic group differences, Table 7 and Figure 3 reveal that the European and North American & Australasian groups generally tend to show higher levels of integration than do the non-European groups. On the friendship indicator, however, several of the European groups (and the North American & Australasian group) fall below the autochthonous majority group, with the South-East European being quite similar to the Middle Eastern, North African & Central Asian, Sub-Saharan African, and South & South-East Asian groups in their pattern of co-ethnic friendships (In the case of the South-East European group, it may be relevant that this includes many Muslims such as Bosniaks and Albanians).

**TABLE 7:** Regression models for social integration (main effects models, ESS7 only).

	No or only some minority people in living area (LPM, raw effects)			No ethnic minority friends (LPM, raw effects)		
	b		SE	b		SE
***Ancestry***						
Only autochthonous ancestry	Ref			Ref		
West European	0.00		0.02	−0.04	*	−0.02
North European	−0.10		0.10	−0.07		−0.01
South European	−0.06	**	0.02	−0.08	***	−0.03
South-East European	−0.11	***	0.03	−0.23	***	−0.07
East European	−0.07	**	0.03	−0.18	***	−0.05
North American & Australasian	−0.04		0.06	−0.12	*	−0.02
MENA & Central Asian	−0.24	***	0.03	−0.27	***	−0.09
Sub-Saharan African	−0.19	***	0.05	−0.22	***	−0.04
South & South-East Asian	−0.16	***	0.04	−0.26	***	−0.06
East Asian	−0.22	**	0.08	−0.14	*	−0.02
Latin American	−0.11		0.06	−0.20	***	−0.03
Caribbean	−0.29	***	0.06	−0.23	***	−0.04
***Generational status***						
1st generation	Ref			Ref		
2nd generation	0.08	***	0.02	0.01		0.01
3rd gen and majority	0.11	***	0.02	0.08	***	0.07
***N***	16,077			16,158		

*p < 0.05, **p < 0.01, ***p < 0.001.

Whether respondents are socially integrated is also significantly related to their generational status. On both indicators the third and higher generations are significantly more likely to be integrated than the first (migrant) generation. The position of the second generation does however differ markedly between the two indicators. In the case of the residential indicator, the second generation is significantly more integrated than the first (as was the case with the structural and cultural indicators), whereas on the friendship indicator the second generation shows little difference from the first generation. This may perhaps indicate a generational lag in the case of social mixing. We therefore conclude that the residential indicator corresponds reasonably well to the straight-line account while the friendship indicator, given the lack of difference between the first and second generations, should perhaps be regarded as consistent with a ‘bumpy line’ account of integration.

### Political Integration

Turning to political integration, we find a rather different picture from structural, cultural or social integration with an absence of the marked differences between European and non-European groups that were evident for the first three dimensions. In the case of voting, we also find some striking differences in the size of the generational differences.

**FIGURE 4:**
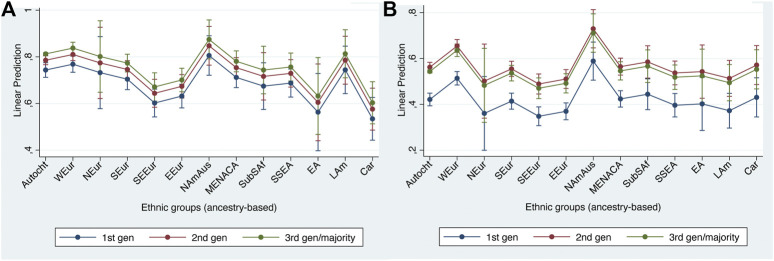
Adjusted predictions at representative values for political integration indicators with 95% confidence intervals. **(A)** Voting; **(B)** non-electoral participation.

Table 8 and Figure 4 again show the regression results and APRs respectively based on the main effects models (i.e., holding generational differences constant across groups). Here we can see that, for both indicators, there is a rather shallow gradient as we move from European to non-European groups, with the main non-European groups having similar levels of political integration to most of the European groups. While the North American & Australasian group stands out once again in a positive direction, it is notable that neither the Middle Eastern, North African & Central Asian nor the Sub-Saharan African groups stand out in the opposite direction.

**TABLE 8:** Regression models for political integration (main effects models).

	Voted in last national election (LPM, raw effects)			Non-electoral participation (LPM, raw effects)		
	b		SE	b		SE
***Ancestry***						
Only autochthonous ancestry	Ref			Ref		
West European	0.02		0.01	0.09	***	0.01
North European	−0.01		0.08	−0.06		0.08
South European	−0.04	*	0.02	−0.01		0.02
South-East European	−0.14	***	0.02	−0.07	**	0.02
East European	−0.11	***	0.03	−0.05	*	0.02
North American & Australasian	0.07		0.05	0.17	***	0.04
MENA & Central Asian	−0.03		0.02	0		0.02
Sub-Saharan African	−0.07		0.05	0.02		0.04
South & South-East Asian	−0.05		0.03	−0.03		0.03
East Asian	−0.18	*	0.08	−0.02		0.06
Latin American	0		0.05	−0.05		0.04
Caribbean	−0.21	***	0.05	0.01		0.04
***Generational status***						
1st generation	Ref			Ref		
2nd generation	0.04	**	0.02	0.14	***	0.01
3rd gen and up	0.07	***	0.02	0.12	***	0.01
***N***	28,443			32,468		

*p < 0.05, **p < 0.01, ***p < 0.001.

The generational effects are in contrast rather different across the two indicators: for voting, the 2nd generation is still markedly less likely to vote than the 3rd and higher generations. For non-electoral political participation, the largest progress is achieved by the 2nd generation, with the third generation statistically indistinguishable. The ‘hurdles’ are thus higher for voting than other forms of political participation.

### Civic Integration

**FIGURE 5:**
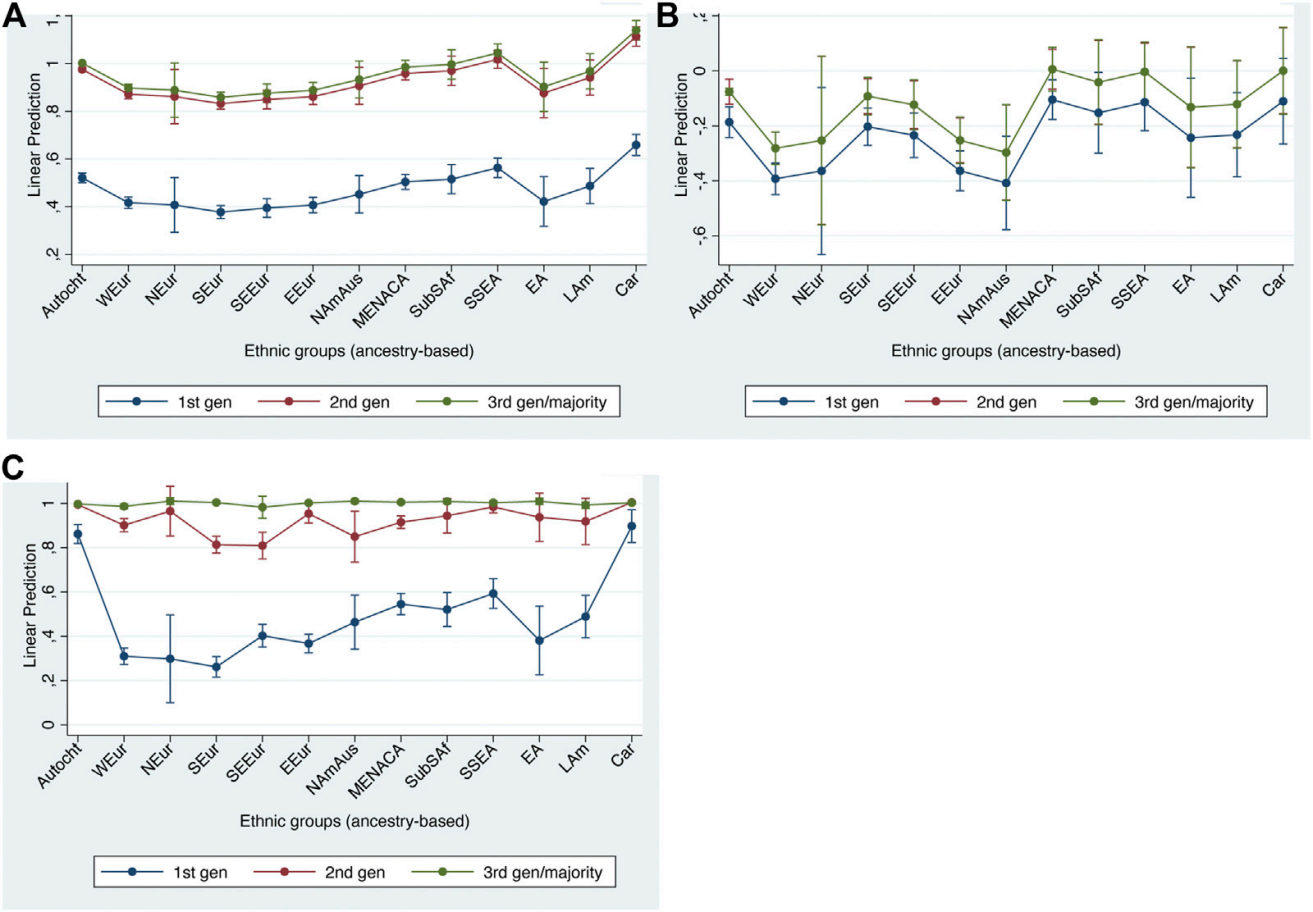
Adjusted predictions at representative values for civic integration indicators with 95% confidence intervals. **(A)** citizenship (main effects model); **(B)** national attachment (main effects model); **(C)** citizenship (interaction effects model).

Finally, we come to our two civic integration indicators – citizenship and national attachment (see Table 9 and Figure 5). As we noted earlier, our measures of model fit showed that, in the case of the citizenship indicator, the model including interaction effects unambiguously yielded a superior fit to the main effects model. However, for consistency with the presentations for the previous dimensions, we begin by the describing the results for the main effects model before turning to predictions from the model with interactions for the citizenship indicator (Figure 5C).

**TABLE 9:** Regression models for civic integration.

	Citizenship (LPM raw effects)						National attachment (OLS raw effects)		
	Main effects model			Interaction effects model			Main effects model		
	**b**		**SE**	**b**		**SE**	**b**		**SE**
***Ancestry***									
Only autochthonous ancestry	Ref			Ref			Ref		
West European	−0.10	***	0.01	−0.55	***	0.03	−0.21	***	0.03
North European	−0.11		0.06	−0.56	***	0.1	−0.18		0.16
South European	−0.14	***	0.01	−0.60	***	0.03	−0.02		0.03
South-East European	−0.13	***	0.02	−0.46	***	0.03	−0.05		0.05
East European	−0.11	***	0.02	−0.49	***	0.03	−0.18	***	0.04
North American & Australasian	−0.07		0.04	−0.40	***	0.07	−0.22	*	0.09
MENA & Central Asian	−0.02		0.01	−0.32	***	0.03	0.08	*	0.04
Sub-Saharan African	−0.01		0.03	−0.34	***	0.04	0.03		0.08
South & South-East Asian	0.04	*	0.02	−0.27	***	0.04	0.07		0.06
East Asian	−0.10		0.05	−0.48	***	0.08	−0.06		0.11
Latin American	−0.03		0.04	−0.37	***	0.05	−0.05		0.08
Caribbean	0.14	***	0.02	0.04		0.04	0.08		0.08
***Generational status***									
1st generation	Ref			Ref			Ref		
2nd generation	0.45	***	0.01	0.13	***	0.02	0.11	***	0.03
3rd gen and up	0.48	***	0.01	0.14	***	0.02	0.11	***	0.03
***Interaction effects*** (***only effects based on n ≥ 10 shown***)									
WEur # 2nd gen				0.46	***	0.03			
WEur # 3rd gen+				0.54	***	0.03			
NEur # 2nd gen				0.53	***	0.12			
SEur # 2nd gen				0.42	***	0.04			
SEur # 3rd gen+				0.61	***	0.03			
SEEur # 2nd gen				0.27	***	0.05			
SEEur # 3rd gen+				0.45	***	0.04			
EEur # 2nd gen				0.45	***	0.04			
EEur # 3rd gen+				0.50	***	0.03			
NAmAus # 2nd gen				0.25	**	0.09			
NAmAus # 3rd gen+				0.41	***	0.07			
MENACA # 2nd gen				0.24	***	0.04			
MENACA # 3rd gen+				0.33	***	0.03			
SubSAf # 2nd gen				0.29	***	0.06			
SubSAf # 3rd gen+				0.35	***	0.04			
SSEA # 2nd gen				0.26	***	0.04			
SSEA # 3rd gen+				0.28	***	0.04			
EA # 2nd gen				0.42	***	0.1			
LAm # 2nd gen				0.30	***	0.08			
Car # 2nd gen				−0.03		0.04			
Car # 3rd gen+				−0.03		0.04			
***N***	32,464			32,464			32,380		

*p < 0.05, **p < 0.01, ***p < 0.001.

There are marked differences between the two indicators in the size of the generational differences, which are much more marked in the case of the citizenship indicator than for the attachment indicator. Nevertheless, the two indicators are fairly similar with respect to the ethnic differences, which are relatively small and with no marked difference between the European and non-European groups. Indeed, if anything, the European groups tend to be less integrated on these two indicators than the non-European groups.

Moving finally to results for citizenship based on the model including interactions between generation and ethnic group, Figure 5C shows a very striking pattern. As we can clearly see, there are very large generational differences among all but one of the non-autochthonous groups contrasting with negligible generational differences among the autochthonous population. This makes very good theoretical sense, and also illustrates the advantage of our measure of ancestry over conventional measures based on country of birth. Thus first-generation members of the autochthonous group, whom we can broadly equate with returnees, are much more likely to have (or to have acquired) citizenship than are first-generation migrants with non-autochthonous ancestry.

The Caribbean exception may well reflect that some Caribbean islands continue to have the citizenship of the previous colonial power, for example the French territories of Guadeloupe, French Guyana and Martinique count as *départements d'Outre mer.* The former Dutch Antilles, although autonomous in many respects, also continue to belong to the Kingdom of the Netherlands and are therefore somewhat comparable to the French situation.

### Robustness Checks

To check whether the results are sensitive to specifications, we ran some robustness checks (results are available in Supplementary Material Appendix A). Firstly, we checked the robustness of our results from linear probability models using logistic regression models. There were only very few instances of effects that were statistically significant in the linear probability but not in the logistic model - namely the negative effect of the Eastern European group with respect to employment, and the negative effects for the North American & Australasian as well as Eastern Asian groups on the friendship indicator. One negative effect, namely for the Latin American group, only became statistically significant in the logistic model of neighborhood segregation. From this we conclude that it is justifiable to focus on the linear probability models, as we did.

As a second robustness check, we constructed a measure of ethnic background based on respondents’ and parents’ countries of birth, classified into the same categories that we have used for ancestry, and re-ran the analyses (figures with APRs for these models are provided in Supplementary Material Appendix C). As we expected, for most first and second-generation respondents, the two measures are in close alignment, although the ancestry measure also identifies a substantial number of first- and second-generation respondents who report autochthonous ancestry only (many of whom will potentially be returnees). Furthermore, the broad patterns of minority integration using the COB measure are very similar to those reported above. However, the ethnic coefficients with the COB measure are sometimes slightly smaller and less statistically significant. More interestingly, we often find that the COB measure produces a few strange negative coefficients for the autochthonous group together with inflated generational effects. This may be a sign of measurement error in the ethnic origin variable being picked up by the generational status variable.

## Discussion

In this paper we have explored ethnic and generational differences in the extent of migrant integration in Western European countries. Regarding ethnic groups, we compared twelve broad panethnic groups with the autochthonous group using a new measure of cultural and ethnic origins (ESCEG) rather than conventional measures based on country of birth. Regarding migrant generations, we compared members of the first, second, and third (including higher) generations. Finally, we compared results across five dimensions of integration which have been prominent in the theoretical literature – structural, cultural, social, political and civic – for each of which we employ two indicators. Table 10 provides an overview of results.

**TABLE 10:** Summary of patterns of the ethnic origin coefficients - ethnic groups compared to autochthonous.

Ethnic group effect	Structural integration		Cultural integration		Social integration		Political integration		Civic integration	
	Labor market	Household income	No homophobia	Gender equality	Neighborhood	Friendship	Voting	Participation	Citizenship	Attachment
Advantaged groups		NAmAus (NEur)	*NAmAus*	NAmAus (NEur)			(NAmAus)	NAmAus		MENACA
		WEur	*NEur*	WEur				WEur		
		SEEur	WEur							
			*SEur*							
Moderately disadvantaged groups (less than or equal to 3rd gen effect)	[EEur]	Car	EEur	EEur	SEEur	SEur (NEur)	SEur	SEEur (NEur)	*SEur*	NAmAus
		EEur			/LAm/(NEur)	WEur		EEur	*NEur*	WEur
					EEur				*WEur*	EEur (NEur)
					SEur				*EEur*	
									*EA*	
									*SEEur*	
									*NAmAus*	
									*LAm*	
									*SubSAf*	
									*MENACA*	
									*SSEA*	
Strongly disadvantaged groups (more than 3rd gen effect)	SubSAf LAm MENACA	SubSAf	MENACA	MENACA	Car	MENACA	Car			
		MENACA	*SubSAf*	SSEA	MENACA	SSEA	EA			
			*SEEur*	SEEur	EA	Car	SEEur			
			SSEA		SubSAf	SEEur	EEur			
					SSEA	SubSAf				
						LAm				
						EEur [EA]				
						[NAmAus]				
Effects of 2nd/3rd + generations	0.05/0.06	0.05/0.06	0.04/0.04	0.04/0.03	0.08/0.11	0.01/0.08	0.04/0.07	0.14/0.12	0.13/0.14 (plus interactions)	0.11/0.11

Notes: Groups ordered by effect size within model. Non-significant effects for groups <50 shown in parentheses () if order of magnitude is comparable to significant effects of other groups i.e., likely due to small cell counts. Effects in brackets [] n.s. in robustness check using logistic model, and effects in slashes//only became significant in logistic model. Effects in italics only in first generation (models with interaction effects). Generational effects were re-calculated by changing the maximum of all dependent variables into 1 (e.g., the coefficient for income deciles was divided by 10).

In a nutshell, our results show that.

1. There are, at least in the first generation, substantial ethnic differences in structural, cultural and social integration, but smaller ethnic differences on the political and civic dimensions;
2. There are, within almost all allochthonous ethnic groups, substantial generational differences in integration, with the first generation being markedly less similar to the majority group (third generation autochthonous) than the second and/or third;
3. Generational patterns of integration do, however, differ across dimensions, with particularly large differences between the first and second generations with respect to citizenship (an indicator of the civic dimension) but little or no difference between the first and second generations with respect to friendship (an indicator of the social dimension).
4. With one important exception, we find that generational differences are broadly similar across the different ethnic groups. The exception relates to the autochthonous group and the citizenship indicators. Whereas we find generational differences among the autochthonous group with respect to structural, cultural, political and social dimensions, they are negligible with respect to citizenship because the first generation autochthonous have very high rates of citizenship already, compared to the allochthonous groups.

In more detail, we find firstly with respect to ethnic differences in integration, that there are substantial overall differences between groups of a European (including North American & Australasian) and those of a non-European background on the structural, cultural and social dimensions. In particular we find that groups with a West European or North American & Australasian background (at least in the first generation) tend to be more advantaged structurally than the majority group in the country of destination, and more liberal culturally too. This is in line with our theoretical expectations, since these groups are ones from highly developed countries with similar democratic histories and liberal cultures. In contrast, the Middle Eastern, North African & Central Asian group together with the Sub-Saharan African group tend to be the most disadvantaged structurally and (together with the South-East European group) the most conservative culturally. The Eastern European group also tends to be somewhat disadvantaged structurally, along with the Caribbean group, and somewhat more conservative culturally. These differences however disappear with the second and/or third generation. While most European groups are not disadvantaged structurally and are as liberal culturally as the autochthonous, lacking social integration, especially on the friendship indicator, affects European groups as well (though to a lesser degree again than culturally more distant or black groups). Our results thus clearly show the benefits of distinguishing between different pan-ethnic groups rather than lumping together all respondents with a migration background, as has been common in some previous research.

The similarity of patterns across these three integration domains could well be because of the causal interrelationships between economic resources, residential patterns, and social mixing: economically disadvantaged minorities are more likely to be concentrated in deprived, multi-ethnic neighborhoods where opportunities for mixing with the majority population will be smaller. There may well also be a reciprocal influence, with lack of bridging social capital inhibiting the acquisition of economic resources. These resource-based arguments are unlikely to apply to the cultural dimension to quite the same extent, although lack of bridging social capital may well inhibit acculturation to the norms and values prevailing among the majority group. Perhaps most surprising is thus the similarity between the structural and cultural dimensions, which most other approaches to integration also distinguish. One possibility is that this similarity is driven in part by the important role of fluency in the destination country language for successful integration: language fluency has been shown to be an important driver of migrant economic success (Dustmann, 1994), and is also likely to be an important factor in exposure to the host-country culture (through the mass media or social contacts). In turn, language acquisition may be facilitated by Western European educational systems. There could also be reciprocal causal relations between economic advance (often related to educational expansion and technological progress) and cultural change. More generally, Inglehart’s research on postmaterialist values has shown that more liberal values are more widespread in more economically advanced societies (Inglehart, 2020).

What is striking, however, is that ethnic differences are much less apparent, even among the first generation, in the case of political and civic integration. While the West European and North American & Australasian groups do exhibit rather higher levels of political participation than the other groups (and than the autochthonous), they are however by no means more integrated on the civic dimension than the non-European groups. Indeed, on both indicators of civic integration, the European groups even tend to fall slightly below the non-European. High national attachment of otherwise disadvantaged minorities may in fact serve to compensate for lack of integration on other domains, especially structural and social integration, which also contribute to a sense of belonging. A further potentially important factor is the ease of return. Especially for EU nationals, return to one’s country of origin is straightforward. For highly skilled migrants, too, such as from North America, migration may be a more temporary project, hindering civic integration. Non-EU migrants in contrast make a more substantial investment in migrating and have greater insecurity. Acquisition of citizenship in turn broadly follows the same rules for all groups, in terms of residential and other criteria (see MIPEX, Huddleston et al., 2015). The right to permanently stay and work in other EU countries however provides less incentive to acquire citizenship for EU nationals. Non-EU migrants also gain more substantial rights by acquiring host country citizenship, explaining their somewhat higher realized access to citizenship.

Turning to political integration, the groups that least participate in voting are groups that are otherwise rarely in the highly disadvantaged set: the East Asian as well as South-East and East European (but also Caribbean) groups. In contrast, the otherwise disadvantaged Middle Eastern, North African & Central Asian as well as Sub-Saharan African groups are politically as active as the autochthonous. This is not in line with theoretical expectations, since many of the respective origin countries are not functioning democracies themselves. However, many individuals leaving these countries may have been politically dissatisfied with this very situation, or even have fled for political reasons. Also, those first-generation migrants that are eligible to vote in their destination countries may again be a positively selected group that will already have gone through the process of naturalization, and may be very motivated to take part in the political process. While self-reports of voting are notoriously unreliable, more detailed work in Britain using measures of validated vote has shown that, among those eligible to vote, turnout of disadvantaged minorities is close to that of the autochthonous group (Heath et al., 2013).

We should therefore take seriously the possibility that integration on the political and civic dimensions is driven by rather different mechanisms than is, for example, the structural dimension. In these integration domains, unlike with structural and social integration, the attitudes and actions of the majority may have less of an impact. In the end, once eligible to vote, voting strongly depends on the individual’s motivation to vote, while friendship, employment and income also depend on other actors, who tend to discriminate against non-white groups (Quillian et al., 2019). Further qualitative research may shed light on the specific reasons for members of different groups to differentially participate in political processes.

Secondly, turning to generational differences, it is striking that in most dimensions and indicators, and for most groups, there tend to be large differences of roughly equal size between the first and second generation (with much smaller differences if any between the second and third generations). This applies clearly to the structural, cultural, political and civic dimensions, and to almost all groups alike. The finding of differences between the first and second generations make very good theoretical sense (and have been found in other cross-national studies of structural integration such as Heath and Cheung, 2007): migrants tend to have lower fluency in the destination language, foreign qualifications and human capital and so on. Their children, brought up in the countries of destination, will have made great progress in language fluency and qualifications, and great exposure to norms and values of the destination country. While racism against non-white groups does persist against the second generation, this seems to have the effect of leaving these groups disadvantaged in the second generation, rather than leading to downwards assimilation. Overall, the generational differences observed in our data are consistent with an account of across-the-board progress for all groups alike, with little indication that non-white or non-Christian groups make less progress between the first and second generations. Whether this is a straight-line or bumpy line pattern is less clear. Especially for interethnic friendship, bumpy-line assimilation appears more plausible given the lack of progress of the second generation. Looking at ethnic and generational effects together, on several indicators (e.g., citizenship, voting) approximate parity with the majority group has been achieved by the second generation already for almost all groups, suggesting that generational progress can quickly compensate for initial ethnic disadvantage. For cultural integration, while some ethnic effects are larger than the generational effects, many are also weaker, and even for those groups from starting points quite different from the majority, parity is reached by the third generation. Therefore, for political and cultural integration, the socialization of the individual may play a more important role than group heritage. In contrast, the ethnic effects tend to be substantially larger than the generational coefficients on the structural and especially social dimensions, suggesting that even in the third generation, substantial ethnic differences persist. For social and structural integration, the (lack of) integration responses on the side of the majority may explain the large ethnic as compared to generational effects.

Thirdly, the case of friendship (an indicator of social integration) does stand out in our data as anomalous with little difference between the first and second generations. Here the starting point is the same across ethnic groups, so that group effects cannot reflect e.g., economic or cultural differences between the origin and destination country. Other research using more robust measures of social mixing are however in line with our findings. Van Tubergen and Smith (2018) have studied the friendship patterns of second-generation young people in England, Germany, the Netherlands and Sweden. They found that in all four countries there were strong tendencies towards ethnic homophily among young people and furthermore that in none of the countries did generational status matter for inter-ethnic friendship (p. 17). They attribute this partly to opportunity structures and partly to socialization within the ethnic community. In conclusion, both majority and minority groups contribute to ethnic group closure since the majority is strongly ethnically segregated as well.

Finally, a striking result is that generational differences are negligible within the autochthonous population with respect to citizenship, while they are more substantial (and similar to those found among the allochthonous population) with respect to all other indicators. These results make sense if we interpret the autochthonous sample with a migration background as returnees. As the example of *pieds noirs* returning to France suggests, displacement from one’s foreign home following the territory’s independence may involve serious economic consequences as well as social disruption. It is also plausible that social and cultural attitudes may have evolved somewhat differently among settlers in the foreign territory than they did in the ‘metropole’, especially when the settlement lasted very long. In contrast, returnees would typically have ready access to citizenship in the metropole (and indeed in the case of the *pieds noirs* would already have citizenship).

Thus many of our results are consistent with the existing evidence on the integration of migrants in Western European countries while our new findings on the integration of people who might be termed autochthonous returnees make good theoretical sense. Our results also serve to vindicate our use of the new ESS measure of ethnic and cultural origins, in place of the conventional approach (at least in Europe) of using country of birth measures. Our robustness check comparing our results with those for country of birth measures show that our measure provides new insights, both on the third generation (which is invisible with the usual COB method) and on the integration experiences of autochthonous returnees. While defenders of the COB method (e.g., Eurostat) typically argue that it is more ‘objective,’ our robustness check indicates that the COB method does not in fact offer any additional explanatory power or more reliable results than our measure of ethnic and cultural origins.

Our multidimensional approach is also vindicated, showing clearly that ethnic and generational differences vary from one dimension to another. In particular there are striking differences in the ethnic patterns between the structural, cultural and social dimensions on the one hand and the political and civic dimensions on the other hand. Generational patterns also differentiate the social dimension (or at least the friendship indicator) from the structural and cultural dimensions.

Perhaps of greatest theoretical interest are the findings on generational differences, and particularly the lack of major interactions between our measure of generational status and of ethnic groups, related to our first research question. Our findings are broadly in line with the idea that ‘generations are the motor of assimilation’ and that it has been the ‘master trend’ among the new postwar immigration in a similar way as it had been among the pre-war immigration of Europeans to the United States. We found no evidence of negative interactions for particular ethnic groups, so that all groups appear to follow the “first path” suggested by Portes and Zhou (Portes and Zhou, 1993). The different context of post-war Europe (with replenishment of many migrants groups for example) from that of pre-war USA where there was a marked slowdown on immigration, does not seem to have held back integration, at least on the structural, cultural, political and civic dimensions.

Concerning our second research question, we thus do not find signs of downward assimilation, while we do find some entrenched structural and social disadvantage in that the Sub-Saharan African group is not yet fully on par with the autochthonous in the third generation. Since this does not equally apply to the Caribbean group (which is only substantially disadvantaged socially), we cannot conclude that racism is the likely driving force behind this structural ethnic disadvantage. For social integration, racism is more likely a relevant factor with all non-white groups being in the strongly disadvantaged set. Also, we find that the mostly Muslim Middle Eastern, North African & Central Asian group also remains disadvantaged into the third generation on the structural, cultural and social domains, so that cultural distance also likely plays its role. With respect to our third research question, we find two ethnic groups that structurally do not differ from the autochthonous, but remain different culturally and socially to some degree, in line with Portes and Zhou’s suggested “third path”: The South-East European and the South & South-East Asian groups. It is however unclear as yet whether Portes and Zhou’s third pattern of rapid economic advancement combined with deliberate preservation of the immigrant community’s values and internal solidarity is a stable long-term pattern or is more a matter of lagged assimilation on the side of social mixing and attitudes. The relative youth of the ‘new’ second-generation (the children of post-1965 migrants) and the sparsity of third or fourth generations in most destination countries means that we are not yet in the position that enabled Warner and Srole (1945) to trace the assimilation of the Irish in America over four generations.

What are then the implications of our findings for segmented assimilation theory? Of the three paths that Portes and Zhou identified, we have found least evidence for downward assimilation in the European context, at least among broad ethnic groups treated as a whole. A more granular analysis than we have been able to conduct might pick up some example of this trajectory, and there are certainly examples across Western Europe of particular second-generation groups some of whose members show disproportionately high rates of school drop-out and subsequent unemployment. But, as we have emphasized, the broad picture is one of improved fortunes among the second generation compared with the first. Our findings do support one important element of segmented assimilation theory though, namely that generational progress may proceed more rapidly on one dimension than another, with friendship lagging behind integration on the other dimensions, at least in the second generation. However, the lack of observed differences between the first and second generations with respect to friendship does seem to operate across-the-board rather than being specific to particular ethnic groups, as in the canonical account of Indian Sikhs (Portes and Zhou, 1993). While we have some misgivings about our indicator for friendship, friendship was the case where we found the least support for interactions between broad ethnic group and generation. Our findings, then, suggest that a rather different account might be needed to explain the similarity across generations, possibly an account based on opportunities for inter-mixing.

There are, to be sure, important limitations to the analyses which we have been able to carry out on the ESS data. Firstly, even with pooled data from ESS 7 and 8, sample sizes for the different broad ethnic groups are relatively small. This limits our statistical power to detect interaction effects (though in general, even where non-significant, the signs of the interaction terms for the supposedly disadvantaged groups are positive rather than the negative signs that critics had anticipated). Sample size also limits our ability to undertake more granular analysis of specific ‘narrow’ ethnic groups in particular destination countries. This highlights the importance of unpacking the first digit European group of the ESCEG, and is a strong argument against an over-simplified white/non-white distinction of ethnic groups.

Secondly, we need to remember that this is cross-sectional data and therefore the first generation are not the actual parents of the second generation, as a strict test of the above-mentioned theories of integration would require. While older members of the first generation could be parents of the second generation, or indeed grandparents of the third generation, this is less likely to hold true for younger members of the first generation. However, controls for age and education should mitigate such problems at least to some degree.

Thirdly, this paper could only study the integration of those immigrants speaking the language(s) of the destination country well enough to participate in the ESS to start with (see also Fitzgerald et al., 2014). The ESS requires countries to offer separate language versions of the questionnaire to all language groups exceeding 5% of the population. Any groups speaking a language smaller than that are not covered. Also, even in the absence of language issues, recent migrants are likely underrepresented in the ESS given their higher rates of geographic mobility and thus difficulty to reach them. Survey nonresponse may also generally be higher amongst structurally disadvantaged, socially segregated or politically disengaged populations. We thus expect that the results reported in this paper represent rather conservative estimates.

Finally, with the pooled sample fixed effects model, we are implicitly assuming that integration outcomes for different groups do not differ across individual countries. Unfortunately, we do not have the statistical power to test whether this assumption is sound. With further rounds of ESS data becoming available, for those integration indicators that were collected in all rounds, more can be done in the near future.

Overall, then, our findings tend to be consistent with the idea of assimilation as a ‘master trend’ today in post-war Europe just as it was in pre-war America, although we would wish to emphasize that our data in no way rules out the possibility that this trend may not apply equally well to more specific minority groups. We also need to qualify this ‘master trend’ with the observation that, even after three generations, some broad groups still lag behind on some important dimensions of integration because of the very different ‘starting points’ of the different immigrant groups. Only with respect to citizenship do we find that parity with the (non-migrant) majority group is clearly established across the board (see Figure 5C). On other dimensions such as the cultural, social and structural, major disparities exist between the majority (and Western European) groups and the second and third generations from some other backgrounds.

Our results clearly need to be replicated in due course, when and if the relevant long-term cohort studies become available. But in the absence of such data, our findings that the different broad ethnic groups show broadly comparable generational differences in integration outcomes is in line with the canonical straight line and bumpy line accounts offered for the pre-war assimilation of European migrants to the United States. In this respect our findings provide a broadly optimistic account of integration in Western Europe. However, with respect to social integration, the outlook is slightly less optimistic given the apparently general tendency for ethnic homophily (van Tubergen and Smith, 2018). Also, in a next step, it would be interesting to look at more specific hypotheses, such as the higher structural disadvantage of Muslim women compared to men.

We retain an open mind, however, as to whether our list of dimensions and indicators might need to be revised. The most pressing requirement is to obtain more reliable indicators on social integration. In addition to direct measures of interethnic friendship, data on patterns of ethnic intermarriage – a classic indicator of social integration – would strengthen the measurement of this dimension. This would likely require usage of different data, more specifically dedicated to researching immigrant integration, rather than a general population sample like the ESS. The indicators of the cultural dimension also need to be strengthened with direct measures of fluency in the destination-country language (or even further indicators of language use) and with measures of religious affiliation (as well as changes therein) and religiosity. As we noted earlier, language fluency is likely to be important for a range of structural outcomes, while religious affiliation and religiosity may well be important for social and cultural outcomes.

As we have demonstrated in this paper, the distinction of socio-cultural groups is a fruitful additional indicator to migrant generation and will, especially in the future, also work better than measures based on country of birth. Such a measure should therefore routinely be included in surveys specifically designed to study processes and outcomes of migrant integration.

## Data Availability

The datasets analyzed for this study can be found on the ESS website at https://www.europeansocialsurvey.org/data/.

## Footnotes

1 The ESS website has an excellent overview of its content and methodology and offers the data for download for free: www.europeansocialsurvey.org

2 The only Western European country not included in ESS rounds 7 and 8 is thus Luxembourg.

3 For ancestries that could be both autochthonous or allochthonous (e.g., Germans in Belgium or Slovaks in Czechia), these were coded as allochthonous if the autochthonous group is very small. Roma and Jews are coded as autochthonous if they are not 1st or 2nd generation migrants. In contrast to Heath et al. (2016) and Schneider and Heath (2019), for this paper those of mixed autochthonous/allochthonous ancestry are assigned to the allochthonous group, rather than the autochthonous group. Those mentioning West European and other European ancestry were coded into the respective other European ancestry. For other European combinations, preference was given in the following order, reflecting cultural distance from the Western European group: South-East European, East European, South European, and North European. Otherwise, the first mentioned ancestry was given preference over the second mentioned one for those who indicated two different allochthonous ancestries.

4 This number is higher than expected, probably reflecting both social desirability bias when responding to this question (despite the normalization statement at the beginning of the question), as well as nonresponse bias (since non-voters are also less likely to participate in surveys), which may also affect the non-electoral political participation measure (Peress, 2010).
