# Supplementary material for: Dimensions of Migrant Integration in Western Europe
Source: Front Sociol. 2021 Apr 29;6:510987. doi: 10.3389/fsoc.2021.510987 (PMC8116888; doi:10.3389/fsoc.2021.510987)
Supplement: Supplementary file 1 [file DataSheet1.zip › Appendix B.html]

Appendix B: The relationship between ancestry and the respondents’ and parents’ country of birth


# Appendix B: The relationship between ancestry and the respondents’ and parents’ country of birth

Silke Schneider

10 October 2019, last updated 17 Dec 2020

```
. *global data "/Users/silke/Documents/gesisbox/Data/ESS/ESS78"
. global data "C:/Users/schneise/Nextcloud/Data/ESS/Analysis datasets"

. global log "C:/Users/schneise/Nextcloud/Markstat"

. use "$data/ESS7e028e01reducedcoded_rev1.dta", clear
```

# Pooled sample (Western European ESS countries only)

How do origin groups relate to generational status? Are the combinations of backgrounds plausible, or do we find signs of measurement error in the ancestry or country of birth measures? *Now we use the revised measure of origin based on ancestry, where 1) for mixed ancestries, the non-European ones are prioritized (and if two non-European ancestries are mentioned, the first-mentioned one is used). For different European ancestries, all pairs are compared and classified depending on the survey country, such as: If West and South/East/South-East/North European ancestry, use South/East/South-East/North European. If combination of not West European ancestries, give priority to culturally most distant ancestry (1) South-East European, (2) East European, (3) South European and (4) North European. This leads to a rather small North European subsample thouhg. 2) “Antillais and other DOM-TOM” are regarded as allochthonous in France.*

```
. tabulate anctryall2r genstat

                      │             genstat
        Ethnic groups │ 0. 3rd ge  2. 2nd ge  5. 1st ge │     Total
──────────────────────┼─────────────────────────────────┼──────────
              Autocht │    24,371      1,537        298 │    26,206 
                 WEur │       657        474        674 │     1,805 
                 NEur │         9         12         24 │        45 
                 SEur │       334        452        353 │     1,139 
                SEEur │        32        196        390 │       618 
                 EEur │       107        129        599 │       835 
               NAmAus │        18         42         71 │       131 
               MENACA │        40        425        488 │       953 
               SubSAf │        10         39        202 │       251 
                 SSEA │        33        156        243 │       432 
                   EA │         8         20         50 │        78 
                  LAm │         7         38        116 │       161 
                  Car │        53         68         61 │       182 
──────────────────────┼─────────────────────────────────┼──────────
                Total │    25,679      3,588      3,569 │    32,836
```

About 1800 cases report no foreign ancestry but a foreign country of birth (self or parent). Let’s look at the combination of country of birth (respondent, mother, father) and ancestry for a few selected countries to find out more about this group.

# Single country analyses

Firstly, in which countries does this issue appear most often?

```
. tab c if anctryall2==0 & genstat!=0

           country │      Freq.     Percent        Cum.
───────────────────┼───────────────────────────────────
        1. Austria │        176        9.47        9.47
        2. Belgium │        182        9.79       19.26
         7. France │        259       13.93       33.19
        8. Germany │        428       23.02       56.21
       15. Ireland │        208       11.19       67.40
   18. Netherlands │        167        8.98       76.39
   26. Switzerland │        189       10.17       86.55
27. United Kingdom │        250       13.45      100.00
───────────────────┼───────────────────────────────────
             Total │      1,859      100.00
```

The group contains more than 200 respondents in France, Germany, Ireland, and the UK.

Secondly, we look at countries we know well, UK, France, and Germany, by checking the joint distribution of country of birth by (ancestry-based) ethnic origins for all respondents who indicated a foreign country of birth.

## UK

Starting with country of birth classified in ESCEG (and irrespective of whether the respondent, mother or father is foreign-born):

```
. tab coball anctryall2r if cntry=="gb"

    country of birth, │
 classified in ESCEG, │
     of respondent or │          Ethnic groups
     mother or father │   Autocht       WEur       SEur │     Total
──────────────────────┼─────────────────────────────────┼──────────
0. no migration backg │     2,997        191          9 │     3,230 
     1. West European │        73        108          0 │       182 
    3. South European │         1          0          6 │         8 
4. South-East Europea │         0          0          0 │         7 
     5. East European │         3          0          0 │        76 
6. North American and │        22          0          1 │        23 
7. MENA and Central A │         2          2          0 │         4 
8. Sub-Saharan Africa │        12          2          1 │        40 
9. South and South-Ea │        24          2          0 │       145 
       10. East Asian │         0          0          0 │         7 
   11. Latin American │         0          0          0 │        10 
        12. Caribbean │         3          0          0 │        22 
──────────────────────┼─────────────────────────────────┼──────────
                Total │     3,137        305         17 │     3,754 


    country of birth, │
 classified in ESCEG, │
     of respondent or │          Ethnic groups
     mother or father │     SEEur       EEur     NAmAus │     Total
──────────────────────┼─────────────────────────────────┼──────────
0. no migration backg │         0         14          0 │     3,230 
     1. West European │         1          0          0 │       182 
    3. South European │         1          0          0 │         8 
4. South-East Europea │         7          0          0 │         7 
     5. East European │         0         73          0 │        76 
6. North American and │         0          0          0 │        23 
7. MENA and Central A │         0          0          0 │         4 
8. Sub-Saharan Africa │         0          0          0 │        40 
9. South and South-Ea │         1          0          0 │       145 
       10. East Asian │         0          0          0 │         7 
   11. Latin American │         0          0          1 │        10 
        12. Caribbean │         0          0          0 │        22 
──────────────────────┼─────────────────────────────────┼──────────
                Total │        10         87          1 │     3,754 


    country of birth, │
 classified in ESCEG, │
     of respondent or │          Ethnic groups
     mother or father │    SubSAf       SSEA         EA │     Total
──────────────────────┼─────────────────────────────────┼──────────
0. no migration backg │         2          5          2 │     3,230 
     1. West European │         0          0          0 │       182 
    3. South European │         0          0          0 │         8 
4. South-East Europea │         0          0          0 │         7 
     5. East European │         0          0          0 │        76 
6. North American and │         0          0          0 │        23 
7. MENA and Central A │         0          0          0 │         4 
8. Sub-Saharan Africa │        16          9          0 │        40 
9. South and South-Ea │         0        117          1 │       145 
       10. East Asian │         0          0          7 │         7 
   11. Latin American │         0          0          0 │        10 
        12. Caribbean │         0          0          0 │        22 
──────────────────────┼─────────────────────────────────┼──────────
                Total │        18        131         10 │     3,754 


    country of birth, │
 classified in ESCEG, │
     of respondent or │     Ethnic groups
     mother or father │       LAm        Car │     Total
──────────────────────┼──────────────────────┼──────────
0. no migration backg │         0         10 │     3,230 
     1. West European │         0          0 │       182 
    3. South European │         0          0 │         8 
4. South-East Europea │         0          0 │         7 
     5. East European │         0          0 │        76 
6. North American and │         0          0 │        23 
7. MENA and Central A │         0          0 │         4 
8. Sub-Saharan Africa │         0          0 │        40 
9. South and South-Ea │         0          0 │       145 
       10. East Asian │         0          0 │         7 
   11. Latin American │         9          0 │        10 
        12. Caribbean │         0         19 │        22 
──────────────────────┼──────────────────────┼──────────
                Total │         9         29 │     3,754
```

What is interesting is that of those with some country of birth in Eastern Europe or the Caribbean, almost all also report Eastern European or Caribbean ancestry respectively. 16% of those with East European ancestry and a third of those with Caribbean ancestry however do not report themselves or one of their parents to be born in Eastern Europe or the Caribbean respectively. So here the measure based on country of birth underestimates the numbers of those with foreign origins. An even more serious problem is the underestimation of those of West European descent living in the UK without any apparent migration background, i.e. belonging to the third and higher generation: while 305 respondents report a West European ancestry, only 108 of them report themselves or one of their parents to be born in Western Europe (except the UK). This underestimation will increase in the future for most of the other groups as well.

Looking at the table the other way round, for those with some country of birth in Western Europe, Sub-Saharan Africa, South and South-East Asia or North America and Australasia, a substantial fraction does not report the respective ancestry. In Sub-Saharan Africa, South and South-East Asia as well as North America and Australasia, the UK formerly had colonial territories so that for these countries, it’s plausible that people of British ancestry were born in these countries. For those born in North America/Australasia (or a parent born there), nobody actually reports the respective ancestry. With respect to Western Europe, British troops in post-war Germany will also have had offspring for whom country of birth would be a bad indicator of ethnic and cultural origin. The more detailed analyses below may shed more light on this.

So for the UK, the apparent inconsistencies in the measures of origin based on ancestry and country of birth are in principle plausible. The question here is thus rather whether the *numbers* of foreign born autochthonous are also plausible, or whether they may be too high.

To check this in more detail, let’s look at the specific countries of birth, where we distinguish between country of birth of the respondent, mother, and father.

```
. tab rcob anctryall2r if cntry=="gb" & rcob!="66"

Country of │                Ethnic groups
     birth │   Autocht       WEur       SEur      SEEur │     Total
───────────┼────────────────────────────────────────────┼──────────
        77 │         0          0          0          0 │         1 
        88 │         1          0          0          0 │         1 
        99 │        51         11         14          1 │       109 
        AU │         4          0          0          0 │         4 
        CN │         0          0          0          0 │         6 
        DE │        12          6          0          0 │        18 
        IE │         3         36          0          1 │        40 
        IN │         4          0          0          1 │        53 
        IR │         0          2          0          0 │         2 
        JM │         2          0          0          0 │         9 
        KE │         0          0          0          0 │         5 
        LK │         2          0          0          0 │         2 
        LT │         0          0          0          0 │         8 
        MY │         1          0          0          0 │         4 
        NG │         1          0          0          0 │         7 
        PH │         0          0          0          0 │         6 
        PK │         2          0          0          0 │        28 
        PL │         1          0          0          0 │        63 
        RO │         0          0          0          7 │         7 
        US │         3          0          1          0 │         4 
        ZA │         3          1          1          0 │        11 
        ZW │         3          1          0          0 │         7 
───────────┼────────────────────────────────────────────┼──────────
     Total │        93         57         16         10 │       395 


Country of │                Ethnic groups
     birth │      EEur     NAmAus     SubSAf       SSEA │     Total
───────────┼────────────────────────────────────────────┼──────────
        77 │         0          0          0          0 │         1 
        88 │         0          0          0          0 │         1 
        99 │         4          0          6          7 │       109 
        AU │         0          0          0          0 │         4 
        CN │         0          0          0          0 │         6 
        DE │         0          0          0          0 │        18 
        IE │         0          0          0          0 │        40 
        IN │         0          0          0         48 │        53 
        IR │         0          0          0          0 │         2 
        JM │         0          0          0          0 │         9 
        KE │         0          0          0          5 │         5 
        LK │         0          0          0          0 │         2 
        LT │         8          0          0          0 │         8 
        MY │         0          0          0          2 │         4 
        NG │         0          0          6          0 │         7 
        PH │         0          1          0          0 │         6 
        PK │         0          0          0         26 │        28 
        PL │        62          0          0          0 │        63 
        RO │         0          0          0          0 │         7 
        US │         0          0          0          0 │         4 
        ZA │         0          0          6          0 │        11 
        ZW │         0          0          1          2 │         7 
───────────┼────────────────────────────────────────────┼──────────
     Total │        74          1         19         90 │       395 


Country of │          Ethnic groups
     birth │        EA        LAm        Car │     Total
───────────┼─────────────────────────────────┼──────────
        77 │         1          0          0 │         1 
        88 │         0          0          0 │         1 
        99 │        10          3          2 │       109 
        AU │         0          0          0 │         4 
        CN │         6          0          0 │         6 
        DE │         0          0          0 │        18 
        IE │         0          0          0 │        40 
        IN │         0          0          0 │        53 
        IR │         0          0          0 │         2 
        JM │         0          0          7 │         9 
        KE │         0          0          0 │         5 
        LK │         0          0          0 │         2 
        LT │         0          0          0 │         8 
        MY │         1          0          0 │         4 
        NG │         0          0          0 │         7 
        PH │         0          5          0 │         6 
        PK │         0          0          0 │        28 
        PL │         0          0          0 │        63 
        RO │         0          0          0 │         7 
        US │         0          0          0 │         4 
        ZA │         0          0          0 │        11 
        ZW │         0          0          0 │         7 
───────────┼─────────────────────────────────┼──────────
     Total │        18          8          9 │       395
```

Of the 4 respondents in the UK who were born in Australia, all report British ancestry. Since most Australians have British ancestry, and those that don’t would unlikely be in the British ESS sample, this is very plausible. All 6 respondents born in China also report East Asian ancestry. 12 out of 18 born in Germany report British ancestry only, and another 6 Western European (likely German/British mixed) ancestry. Most of those born in Ireland report Western European ancestry, rather than British. The large majority of those born in India or Pakistan also report South and South-East Asian ancestry, and almost all of those born in Poland East European ancestry. Etc. For respondents country of birth and ancestry, the combination of measures leads to highly plausible results.

```
. tab mcob anctryall2r if cntry=="gb" & mcob!="66"

Country of │
    birth, │                Ethnic groups
    mother │   Autocht       WEur       SEur      SEEur │     Total
───────────┼────────────────────────────────────────────┼──────────
        77 │         0          0          0          0 │         1 
        88 │         4          0          0          0 │         7 
        99 │        50         11          8          1 │        91 
        BD │         1          0          0          0 │         8 
        CN │         0          0          0          0 │        12 
        DE │         7          8          0          0 │        15 
        ES │         1          0          0          1 │         2 
        GH │         3          0          0          0 │         3 
        IE │        24         78          0          0 │       102 
        IN │        10          1          0          1 │        89 
        IQ │         1          0          0          0 │         1 
        IT │         0          0         13          0 │        13 
        JM │         3          0          0          0 │        23 
        KE │         1          0          0          0 │         5 
        LK │         4          1          0          0 │         5 
        LT │         0          0          0          0 │         8 
        NG │         1          0          0          0 │        14 
        PH │         0          0          0          0 │        12 
        PK │         3          0          0          0 │        38 
        PL │         1          1          0          0 │        64 
        RO │         0          0          0          7 │         7 
        US │         4          0          1          0 │         5 
        ZA │         1          0          1          0 │         8 
        ZW │         0          0          0          0 │         2 
───────────┼────────────────────────────────────────────┼──────────
     Total │       119        100         23         10 │       535 


Country of │
    birth, │                Ethnic groups
    mother │      EEur     NAmAus     SubSAf       SSEA │     Total
───────────┼────────────────────────────────────────────┼──────────
        77 │         0          0          0          0 │         1 
        88 │         0          0          1          1 │         7 
        99 │         3          0          0          9 │        91 
        BD │         0          0          0          7 │         8 
        CN │         0          0          0          0 │        12 
        DE │         0          0          0          0 │        15 
        ES │         0          0          0          0 │         2 
        GH │         0          0          0          0 │         3 
        IE │         0          0          0          0 │       102 
        IN │         0          0          0         77 │        89 
        IQ │         0          0          0          0 │         1 
        IT │         0          0          0          0 │        13 
        JM │         0          0          0          0 │        23 
        KE │         0          0          0          4 │         5 
        LK │         0          0          0          0 │         5 
        LT │         8          0          0          0 │         8 
        NG │         0          0         13          0 │        14 
        PH │         0          1          0          0 │        12 
        PK │         0          0          0         35 │        38 
        PL │        62          0          0          0 │        64 
        RO │         0          0          0          0 │         7 
        US │         0          0          0          0 │         5 
        ZA │         0          0          6          0 │         8 
        ZW │         0          0          0          2 │         2 
───────────┼────────────────────────────────────────────┼──────────
     Total │        73          1         20        135 │       535 


Country of │
    birth, │          Ethnic groups
    mother │        EA        LAm        Car │     Total
───────────┼─────────────────────────────────┼──────────
        77 │         1          0          0 │         1 
        88 │         1          0          0 │         7 
        99 │         6          0          3 │        91 
        BD │         0          0          0 │         8 
        CN │        12          0          0 │        12 
        DE │         0          0          0 │        15 
        ES │         0          0          0 │         2 
        GH │         0          0          0 │         3 
        IE │         0          0          0 │       102 
        IN │         0          0          0 │        89 
        IQ │         0          0          0 │         1 
        IT │         0          0          0 │        13 
        JM │         0          0         20 │        23 
        KE │         0          0          0 │         5 
        LK │         0          0          0 │         5 
        LT │         0          0          0 │         8 
        NG │         0          0          0 │        14 
        PH │         0         11          0 │        12 
        PK │         0          0          0 │        38 
        PL │         0          0          0 │        64 
        RO │         0          0          0 │         7 
        US │         0          0          0 │         5 
        ZA │         0          0          0 │         8 
        ZW │         0          0          0 │         2 
───────────┼─────────────────────────────────┼──────────
     Total │        20         11         23 │       535 

. tab fcob anctryall2r if cntry=="gb" & fcob!="66"

Country of │
    birth, │                Ethnic groups
    father │   Autocht       WEur       SEur      SEEur │     Total
───────────┼────────────────────────────────────────────┼──────────
        77 │         0          0          0          0 │         1 
        88 │         6          0          1          0 │        11 
        99 │        52          9         13          1 │        96 
        BD │         1          0          0          0 │         8 
        CA │         4          0          0          0 │         4 
        CN │         0          0          0          0 │        11 
        DE │         3          8          0          0 │        13 
        GH │         3          0          0          0 │         3 
        IE │        39         74          0          1 │       114 
        IN │        11          0          0          1 │        96 
        IQ │         1          0          0          0 │         1 
        IR │         1          1          0          0 │         2 
        IT │         0          0          6          0 │         6 
        JM │         3          0          0          0 │        24 
        KE │         2          0          0          0 │         5 
        LK │         5          0          0          0 │         5 
        LT │         0          0          0          0 │         8 
        NG │         1          0          0          0 │        15 
        PH │         0          0          0          0 │        12 
        PK │         3          0          0          0 │        38 
        PL │         4          0          0          0 │        67 
        RO │         0          0          0          7 │         7 
        US │        15          1          1          0 │        18 
        ZW │         2          1          0          0 │         5 
───────────┼────────────────────────────────────────────┼──────────
     Total │       156         94         21         10 │       570 


Country of │
    birth, │                Ethnic groups
    father │      EEur     NAmAus     SubSAf       SSEA │     Total
───────────┼────────────────────────────────────────────┼──────────
        77 │         0          0          0          0 │         1 
        88 │         0          0          1          1 │        11 
        99 │         2          0          3          6 │        96 
        BD │         0          0          0          7 │         8 
        CA │         0          0          0          0 │         4 
        CN │         0          0          0          0 │        11 
        DE │         1          0          0          0 │        13 
        GH │         0          0          0          0 │         3 
        IE │         0          0          0          0 │       114 
        IN │         0          0          0         84 │        96 
        IQ │         0          0          0          0 │         1 
        IR │         0          0          0          0 │         2 
        IT │         0          0          0          0 │         6 
        JM │         0          0          0          0 │        24 
        KE │         0          0          0          3 │         5 
        LK │         0          0          0          0 │         5 
        LT │         8          0          0          0 │         8 
        NG │         0          0         14          0 │        15 
        PH │         0          1          0          0 │        12 
        PK │         0          0          0         35 │        38 
        PL │        63          0          0          0 │        67 
        RO │         0          0          0          0 │         7 
        US │         1          0          0          0 │        18 
        ZW │         0          0          2          0 │         5 
───────────┼────────────────────────────────────────────┼──────────
     Total │        75          1         20        136 │       570 


Country of │
    birth, │          Ethnic groups
    father │        EA        LAm        Car │     Total
───────────┼─────────────────────────────────┼──────────
        77 │         1          0          0 │         1 
        88 │         1          0          1 │        11 
        99 │         6          0          4 │        96 
        BD │         0          0          0 │         8 
        CA │         0          0          0 │         4 
        CN │        11          0          0 │        11 
        DE │         1          0          0 │        13 
        GH │         0          0          0 │         3 
        IE │         0          0          0 │       114 
        IN │         0          0          0 │        96 
        IQ │         0          0          0 │         1 
        IR │         0          0          0 │         2 
        IT │         0          0          0 │         6 
        JM │         0          0         21 │        24 
        KE │         0          0          0 │         5 
        LK │         0          0          0 │         5 
        LT │         0          0          0 │         8 
        NG │         0          0          0 │        15 
        PH │         0         11          0 │        12 
        PK │         0          0          0 │        38 
        PL │         0          0          0 │        67 
        RO │         0          0          0 │         7 
        US │         0          0          0 │        18 
        ZW │         0          0          0 │         5 
───────────┼─────────────────────────────────┼──────────
     Total │        20         11         26 │       570
```

Looking at mothers’ and fathers’ country of birth, the results are maybe somewhat less plausible, but it’s hard to say. For example, 102 (114) respondents report that their mother (father) was born in Ireland. Out of these, 24 (39) at the same time report British ancestry only. So this would imply that their British mothers (fathers) were born in Ireland (not Northern Ireland, unless respondents report the latter as the former). As another example, 89 (96) respondents report that their mother (father) was born in India. Out of these, 10 (11) at the same time report British ancestry only, i.e. their British mothers and fathers would have been born in India. On the other hand, 64 (67) of those who report that their mother (father) was born in Poland, 62 (63) report East European ancestry, which given there were no British territories in Eastern Europe, looks very plausible.

Note that each country of birth is mentioned by at least 2 respondents, and that quite a high number of people do not have a valid code on the country of birth variable (30-50%!). Those with missing data on the specific country of birth could not be classified in ESCEG (unless the information is non-missing for a parent and consistent with respondents’ country of birth). So they will be recorded as having a migration background, but may appear like “no migration background” in the variable measuring ESCEG based on country of birth (‘coball’ in first table for the UK).

## Ireland

```
. tab coball anctryall2r if cntry=="ie"

    country of birth, │
 classified in ESCEG, │
     of respondent or │          Ethnic groups
     mother or father │   Autocht       WEur       NEur │     Total
──────────────────────┼─────────────────────────────────┼──────────
0. no migration backg │     3,985        115          0 │     4,122 
     1. West European │       194        263          0 │       473 
    2. North European │         0          0          1 │         1 
    3. South European │         2          0          0 │         9 
4. South-East Europea │         1          0          0 │        18 
     5. East European │         2          0          0 │       190 
6. North American and │        24          5          0 │        44 
7. MENA and Central A │         0          0          0 │         4 
8. Sub-Saharan Africa │         3          2          0 │        39 
9. South and South-Ea │         1          0          0 │        63 
       10. East Asian │         0          0          0 │         3 
   11. Latin American │         0          0          0 │        27 
──────────────────────┼─────────────────────────────────┼──────────
                Total │     4,212        385          1 │     4,993 


    country of birth, │
 classified in ESCEG, │
     of respondent or │          Ethnic groups
     mother or father │      SEur      SEEur       EEur │     Total
──────────────────────┼─────────────────────────────────┼──────────
0. no migration backg │         8          0          3 │     4,122 
     1. West European │         5          0          1 │       473 
    2. North European │         0          0          0 │         1 
    3. South European │         6          0          0 │         9 
4. South-East Europea │         0         16          1 │        18 
     5. East European │         0          0        187 │       190 
6. North American and │         0          0          0 │        44 
7. MENA and Central A │         0          0          1 │         4 
8. Sub-Saharan Africa │         0          0          0 │        39 
9. South and South-Ea │         0          0          0 │        63 
       10. East Asian │         0          0          0 │         3 
   11. Latin American │         0          0          0 │        27 
──────────────────────┼─────────────────────────────────┼──────────
                Total │        19         16        193 │     4,993 


    country of birth, │
 classified in ESCEG, │
     of respondent or │          Ethnic groups
     mother or father │    NAmAus     MENACA     SubSAf │     Total
──────────────────────┼─────────────────────────────────┼──────────
0. no migration backg │         2          0          2 │     4,122 
     1. West European │         5          1          0 │       473 
    2. North European │         0          0          0 │         1 
    3. South European │         0          0          0 │         9 
4. South-East Europea │         0          0          0 │        18 
     5. East European │         1          0          0 │       190 
6. North American and │        14          0          0 │        44 
7. MENA and Central A │         0          2          0 │         4 
8. Sub-Saharan Africa │         0          0         34 │        39 
9. South and South-Ea │         1          0          0 │        63 
       10. East Asian │         0          0          0 │         3 
   11. Latin American │         0          0          0 │        27 
──────────────────────┼─────────────────────────────────┼──────────
                Total │        23          3         36 │     4,993 


    country of birth, │
 classified in ESCEG, │
     of respondent or │          Ethnic groups
     mother or father │      SSEA         EA        LAm │     Total
──────────────────────┼─────────────────────────────────┼──────────
0. no migration backg │         6          1          0 │     4,122 
     1. West European │         3          0          0 │       473 
    2. North European │         0          0          0 │         1 
    3. South European │         0          0          1 │         9 
4. South-East Europea │         0          0          0 │        18 
     5. East European │         0          0          0 │       190 
6. North American and │         1          0          0 │        44 
7. MENA and Central A │         1          0          0 │         4 
8. Sub-Saharan Africa │         0          0          0 │        39 
9. South and South-Ea │        60          1          0 │        63 
       10. East Asian │         0          3          0 │         3 
   11. Latin American │         0          0         27 │        27 
──────────────────────┼─────────────────────────────────┼──────────
                Total │        71          5         28 │     4,993 


    country of birth, │
 classified in ESCEG, │   Ethnic
     of respondent or │   groups
     mother or father │       Car │     Total
──────────────────────┼───────────┼──────────
0. no migration backg │         0 │     4,122 
     1. West European │         1 │       473 
    2. North European │         0 │         1 
    3. South European │         0 │         9 
4. South-East Europea │         0 │        18 
     5. East European │         0 │       190 
6. North American and │         0 │        44 
7. MENA and Central A │         0 │         4 
8. Sub-Saharan Africa │         0 │        39 
9. South and South-Ea │         0 │        63 
       10. East Asian │         0 │         3 
   11. Latin American │         0 │        27 
──────────────────────┼───────────┼──────────
                Total │         1 │     4,993 

. tab rcob anctryall2r if cntry=="ie" & rcob!="66"

Country of │                Ethnic groups
     birth │   Autocht       WEur       NEur       SEur │     Total
───────────┼────────────────────────────────────────────┼──────────
        77 │         0          0          0          0 │         1 
        99 │         9          6          1          6 │        86 
        AF │         0          0          0          0 │         1 
        AU │         6          1          0          0 │        10 
        BD │         0          0          0          0 │         1 
        BR │         0          0          0          0 │        16 
        CA │         1          0          0          0 │         1 
        CG │         0          0          0          0 │         1 
        CN │         0          0          0          0 │         2 
        CZ │         0          0          0          0 │         5 
        DE │         0         14          0          0 │        15 
        ES │         0          0          0          3 │         3 
        ET │         0          0          0          0 │         1 
        FR │         0         12          0          1 │        14 
        GB │        98        154          0          1 │       259 
        GH │         0          0          0          0 │         2 
        GN │         0          0          0          0 │         1 
        HU │         0          0          0          0 │         2 
        IM │         1          0          0          0 │         1 
        IN │         1          0          0          0 │        45 
        IT │         0          0          0          2 │         2 
        KW │         0          0          0          0 │         1 
        KZ │         0          0          0          0 │         1 
        LT │         0          0          0          0 │        22 
        LV │         0          0          0          0 │        16 
        MD │         0          0          0          0 │         1 
        MN │         0          0          0          0 │         1 
        MU │         0          0          0          0 │         1 
        MY │         0          0          0          0 │         4 
        NG │         0          1          0          0 │        24 
        NL │         0          6          0          0 │         6 
        NZ │         0          1          0          0 │         2 
        PH │         0          0          0          0 │         8 
        PK │         0          0          0          0 │         4 
        PL │         0          0          0          0 │       136 
        PT │         0          0          0          1 │         1 
        RO │         1          0          0          0 │        15 
        RU │         0          0          0          0 │         1 
        SD │         0          0          0          0 │         1 
        TH │         0          0          0          0 │         1 
        UA │         0          0          0          0 │         2 
        US │         6          2          0          0 │        17 
        VE │         0          0          0          0 │         3 
        ZA │         0          1          0          0 │         3 
        ZW │         0          0          0          0 │         1 
───────────┼────────────────────────────────────────────┼──────────
     Total │       123        198          1         14 │       741 


Country of │                Ethnic groups
     birth │     SEEur       EEur     NAmAus     MENACA │     Total
───────────┼────────────────────────────────────────────┼──────────
        77 │         0          1          0          0 │         1 
        99 │         6         15          6          5 │        86 
        AF │         0          0          0          1 │         1 
        AU │         0          0          3          0 │        10 
        BD │         0          0          0          0 │         1 
        BR │         0          0          0          0 │        16 
        CA │         0          0          0          0 │         1 
        CG │         0          0          0          0 │         1 
        CN │         0          0          0          0 │         2 
        CZ │         0          5          0          0 │         5 
        DE │         0          1          0          0 │        15 
        ES │         0          0          0          0 │         3 
        ET │         0          0          0          0 │         1 
        FR │         0          0          0          0 │        14 
        GB │         0          0          3          0 │       259 
        GH │         0          0          0          0 │         2 
        GN │         0          0          0          0 │         1 
        HU │         0          2          0          0 │         2 
        IM │         0          0          0          0 │         1 
        IN │         0          0          1          0 │        45 
        IT │         0          0          0          0 │         2 
        KW │         0          0          0          1 │         1 
        KZ │         0          1          0          0 │         1 
        LT │         0         21          1          0 │        22 
        LV │         0         16          0          0 │        16 
        MD │         1          0          0          0 │         1 
        MN │         0          0          0          0 │         1 
        MU │         0          0          0          0 │         1 
        MY │         0          0          0          0 │         4 
        NG │         0          0          0          0 │        24 
        NL │         0          0          0          0 │         6 
        NZ │         0          0          1          0 │         2 
        PH │         0          0          0          0 │         8 
        PK │         0          0          0          0 │         4 
        PL │         0        136          0          0 │       136 
        PT │         0          0          0          0 │         1 
        RO │        13          1          0          0 │        15 
        RU │         0          1          0          0 │         1 
        SD │         0          0          0          0 │         1 
        TH │         0          0          0          0 │         1 
        UA │         0          2          0          0 │         2 
        US │         0          0          8          0 │        17 
        VE │         0          0          0          0 │         3 
        ZA │         0          0          0          0 │         3 
        ZW │         0          0          0          0 │         1 
───────────┼────────────────────────────────────────────┼──────────
     Total │        20        202         23          7 │       741 


Country of │                Ethnic groups
     birth │    SubSAf       SSEA         EA        LAm │     Total
───────────┼────────────────────────────────────────────┼──────────
        77 │         0          0          0          0 │         1 
        99 │         8         23          0          1 │        86 
        AF │         0          0          0          0 │         1 
        AU │         0          0          0          0 │        10 
        BD │         0          1          0          0 │         1 
        BR │         0          0          0         16 │        16 
        CA │         0          0          0          0 │         1 
        CG │         1          0          0          0 │         1 
        CN │         0          0          2          0 │         2 
        CZ │         0          0          0          0 │         5 
        DE │         0          0          0          0 │        15 
        ES │         0          0          0          0 │         3 
        ET │         1          0          0          0 │         1 
        FR │         0          0          0          0 │        14 
        GB │         0          3          0          0 │       259 
        GH │         2          0          0          0 │         2 
        GN │         1          0          0          0 │         1 
        HU │         0          0          0          0 │         2 
        IM │         0          0          0          0 │         1 
        IN │         0         43          0          0 │        45 
        IT │         0          0          0          0 │         2 
        KW │         0          0          0          0 │         1 
        KZ │         0          0          0          0 │         1 
        LT │         0          0          0          0 │        22 
        LV │         0          0          0          0 │        16 
        MD │         0          0          0          0 │         1 
        MN │         0          0          1          0 │         1 
        MU │         0          1          0          0 │         1 
        MY │         0          3          1          0 │         4 
        NG │        23          0          0          0 │        24 
        NL │         0          0          0          0 │         6 
        NZ │         0          0          0          0 │         2 
        PH │         0          0          0          8 │         8 
        PK │         0          4          0          0 │         4 
        PL │         0          0          0          0 │       136 
        PT │         0          0          0          0 │         1 
        RO │         0          0          0          0 │        15 
        RU │         0          0          0          0 │         1 
        SD │         1          0          0          0 │         1 
        TH │         0          1          0          0 │         1 
        UA │         0          0          0          0 │         2 
        US │         0          1          0          0 │        17 
        VE │         0          0          0          3 │         3 
        ZA │         2          0          0          0 │         3 
        ZW │         1          0          0          0 │         1 
───────────┼────────────────────────────────────────────┼──────────
     Total │        40         80          4         28 │       741 


           │   Ethnic
Country of │   groups
     birth │       Car │     Total
───────────┼───────────┼──────────
        77 │         0 │         1 
        99 │         0 │        86 
        AF │         0 │         1 
        AU │         0 │        10 
        BD │         0 │         1 
        BR │         0 │        16 
        CA │         0 │         1 
        CG │         0 │         1 
        CN │         0 │         2 
        CZ │         0 │         5 
        DE │         0 │        15 
        ES │         0 │         3 
        ET │         0 │         1 
        FR │         1 │        14 
        GB │         0 │       259 
        GH │         0 │         2 
        GN │         0 │         1 
        HU │         0 │         2 
        IM │         0 │         1 
        IN │         0 │        45 
        IT │         0 │         2 
        KW │         0 │         1 
        KZ │         0 │         1 
        LT │         0 │        22 
        LV │         0 │        16 
        MD │         0 │         1 
        MN │         0 │         1 
        MU │         0 │         1 
        MY │         0 │         4 
        NG │         0 │        24 
        NL │         0 │         6 
        NZ │         0 │         2 
        PH │         0 │         8 
        PK │         0 │         4 
        PL │         0 │       136 
        PT │         0 │         1 
        RO │         0 │        15 
        RU │         0 │         1 
        SD │         0 │         1 
        TH │         0 │         1 
        UA │         0 │         2 
        US │         0 │        17 
        VE │         0 │         3 
        ZA │         0 │         3 
        ZW │         0 │         1 
───────────┼───────────┼──────────
     Total │         1 │       741 

. tab mcob anctryall2r if cntry=="ie" & mcob!="66"

Country of │
    birth, │                Ethnic groups
    mother │   Autocht       WEur       NEur       SEur │     Total
───────────┼────────────────────────────────────────────┼──────────
        77 │         1          0          0          0 │         1 
        99 │         8         12          1          6 │        62 
        AF │         0          0          0          0 │         1 
        AU │         1          0          0          0 │         1 
        BD │         0          0          0          0 │         7 
        BR │         0          0          0          0 │        16 
        BY │         0          0          0          0 │         1 
        CA │         1          0          0          0 │         1 
        CG │         0          0          0          0 │         1 
        CH │         0          1          0          0 │         1 
        CN │         0          0          0          0 │         3 
        CZ │         0          0          0          0 │         5 
        DE │         2         15          0          0 │        19 
        ES │         0          0          0          2 │         2 
        ET │         0          0          0          0 │         1 
        FR │         0         14          0          1 │        16 
        GB │        63        147          0          2 │       220 
        GH │         0          0          0          0 │         2 
        GN │         0          0          0          0 │         1 
        HK │         0          1          0          0 │         1 
        HU │         0          0          0          0 │         7 
        IN │         0          0          0          0 │        51 
        IT │         0          0          0          2 │         2 
        KW │         0          0          0          0 │         1 
        KZ │         0          0          0          0 │         1 
        LT │         0          0          0          0 │        20 
        LV │         1          0          0          0 │        19 
        MD │         0          0          0          0 │         1 
        MN │         0          0          0          0 │         1 
        MU │         0          0          0          0 │         1 
        MY │         0          0          0          0 │         4 
        NG │         1          0          0          0 │        25 
        NL │         0          1          0          0 │         1 
        NP │         0          0          0          0 │         5 
        NZ │         0          1          0          0 │         3 
        PH │         0          0          0          0 │         8 
        PK │         0          0          0          0 │        13 
        PL │         0          0          0          0 │       132 
        PT │         0          0          0          1 │         1 
        RO │         1          0          0          0 │        15 
        RU │         0          0          0          0 │         8 
        SD │         0          0          0          0 │         1 
        UA │         0          0          0          0 │         2 
        US │         8          4          0          0 │        24 
        VE │         0          0          0          0 │         3 
        ZA │         0          0          0          0 │         2 
        ZW │         0          0          0          0 │         1 
───────────┼────────────────────────────────────────────┼──────────
     Total │        87        196          1         14 │       714 


Country of │
    birth, │                Ethnic groups
    mother │     SEEur       EEur     NAmAus     MENACA │     Total
───────────┼────────────────────────────────────────────┼──────────
        77 │         0          0          0          0 │         1 
        99 │         6          6          4          7 │        62 
        AF │         0          0          0          1 │         1 
        AU │         0          0          0          0 │         1 
        BD │         0          0          0          0 │         7 
        BR │         0          0          0          0 │        16 
        BY │         0          1          0          0 │         1 
        CA │         0          0          0          0 │         1 
        CG │         0          0          0          0 │         1 
        CH │         0          0          0          0 │         1 
        CN │         0          0          0          0 │         3 
        CZ │         0          5          0          0 │         5 
        DE │         0          2          0          0 │        19 
        ES │         0          0          0          0 │         2 
        ET │         0          0          0          0 │         1 
        FR │         0          0          0          0 │        16 
        GB │         0          1          5          1 │       220 
        GH │         0          0          0          0 │         2 
        GN │         0          0          0          0 │         1 
        HK │         0          0          0          0 │         1 
        HU │         0          7          0          0 │         7 
        IN │         0          0          1          0 │        51 
        IT │         0          0          0          0 │         2 
        KW │         0          0          0          1 │         1 
        KZ │         0          1          0          0 │         1 
        LT │         0         20          0          0 │        20 
        LV │         0         18          0          0 │        19 
        MD │         1          0          0          0 │         1 
        MN │         0          0          0          0 │         1 
        MU │         0          0          0          0 │         1 
        MY │         0          0          0          0 │         4 
        NG │         0          0          0          0 │        25 
        NL │         0          0          0          0 │         1 
        NP │         0          0          0          0 │         5 
        NZ │         0          0          2          0 │         3 
        PH │         0          0          0          0 │         8 
        PK │         0          0          0          0 │        13 
        PL │         0        132          0          0 │       132 
        PT │         0          0          0          0 │         1 
        RO │        13          1          0          0 │        15 
        RU │         0          7          1          0 │         8 
        SD │         0          0          0          0 │         1 
        UA │         0          2          0          0 │         2 
        US │         0          0         11          0 │        24 
        VE │         0          0          0          0 │         3 
        ZA │         0          0          0          0 │         2 
        ZW │         0          0          0          0 │         1 
───────────┼────────────────────────────────────────────┼──────────
     Total │        20        203         24         10 │       714 


Country of │
    birth, │                Ethnic groups
    mother │    SubSAf       SSEA         EA        LAm │     Total
───────────┼────────────────────────────────────────────┼──────────
        77 │         0          0          0          0 │         1 
        99 │         8          3          0          1 │        62 
        AF │         0          0          0          0 │         1 
        AU │         0          0          0          0 │         1 
        BD │         0          7          0          0 │         7 
        BR │         0          0          0         16 │        16 
        BY │         0          0          0          0 │         1 
        CA │         0          0          0          0 │         1 
        CG │         1          0          0          0 │         1 
        CH │         0          0          0          0 │         1 
        CN │         0          1          2          0 │         3 
        CZ │         0          0          0          0 │         5 
        DE │         0          0          0          0 │        19 
        ES │         0          0          0          0 │         2 
        ET │         1          0          0          0 │         1 
        FR │         0          0          0          0 │        16 
        GB │         0          1          0          0 │       220 
        GH │         2          0          0          0 │         2 
        GN │         1          0          0          0 │         1 
        HK │         0          0          0          0 │         1 
        HU │         0          0          0          0 │         7 
        IN │         0         50          0          0 │        51 
        IT │         0          0          0          0 │         2 
        KW │         0          0          0          0 │         1 
        KZ │         0          0          0          0 │         1 
        LT │         0          0          0          0 │        20 
        LV │         0          0          0          0 │        19 
        MD │         0          0          0          0 │         1 
        MN │         0          0          1          0 │         1 
        MU │         0          1          0          0 │         1 
        MY │         0          3          1          0 │         4 
        NG │        24          0          0          0 │        25 
        NL │         0          0          0          0 │         1 
        NP │         0          5          0          0 │         5 
        NZ │         0          0          0          0 │         3 
        PH │         0          0          0          8 │         8 
        PK │         0         13          0          0 │        13 
        PL │         0          0          0          0 │       132 
        PT │         0          0          0          0 │         1 
        RO │         0          0          0          0 │        15 
        RU │         0          0          0          0 │         8 
        SD │         1          0          0          0 │         1 
        UA │         0          0          0          0 │         2 
        US │         0          1          0          0 │        24 
        VE │         0          0          0          3 │         3 
        ZA │         2          0          0          0 │         2 
        ZW │         1          0          0          0 │         1 
───────────┼────────────────────────────────────────────┼──────────
     Total │        41         85          4         28 │       714 


Country of │   Ethnic
    birth, │   groups
    mother │       Car │     Total
───────────┼───────────┼──────────
        77 │         0 │         1 
        99 │         0 │        62 
        AF │         0 │         1 
        AU │         0 │         1 
        BD │         0 │         7 
        BR │         0 │        16 
        BY │         0 │         1 
        CA │         0 │         1 
        CG │         0 │         1 
        CH │         0 │         1 
        CN │         0 │         3 
        CZ │         0 │         5 
        DE │         0 │        19 
        ES │         0 │         2 
        ET │         0 │         1 
        FR │         1 │        16 
        GB │         0 │       220 
        GH │         0 │         2 
        GN │         0 │         1 
        HK │         0 │         1 
        HU │         0 │         7 
        IN │         0 │        51 
        IT │         0 │         2 
        KW │         0 │         1 
        KZ │         0 │         1 
        LT │         0 │        20 
        LV │         0 │        19 
        MD │         0 │         1 
        MN │         0 │         1 
        MU │         0 │         1 
        MY │         0 │         4 
        NG │         0 │        25 
        NL │         0 │         1 
        NP │         0 │         5 
        NZ │         0 │         3 
        PH │         0 │         8 
        PK │         0 │        13 
        PL │         0 │       132 
        PT │         0 │         1 
        RO │         0 │        15 
        RU │         0 │         8 
        SD │         0 │         1 
        UA │         0 │         2 
        US │         0 │        24 
        VE │         0 │         3 
        ZA │         0 │         2 
        ZW │         0 │         1 
───────────┼───────────┼──────────
     Total │         1 │       714 

. tab fcob anctryall2r if cntry=="ie" & fcob!="66"

Country of │
    birth, │                Ethnic groups
    father │   Autocht       WEur       NEur       SEur │     Total
───────────┼────────────────────────────────────────────┼──────────
        77 │         1          0          0          0 │         1 
        99 │         5          8          1          2 │        59 
        AF │         0          0          0          0 │         1 
        AU │         0          0          0          0 │         1 
        BD │         0          0          0          0 │         7 
        BE │         0          1          0          0 │         1 
        BR │         0          0          0          0 │        14 
        CA │         1          0          0          0 │         1 
        CG │         0          0          0          0 │         1 
        CH │         0          1          0          0 │         1 
        CN │         0          0          0          0 │         3 
        CZ │         0          0          0          0 │         5 
        DE │         1         16          0          0 │        19 
        ES │         0          0          0          3 │         3 
        ET │         0          0          0          0 │         1 
        FR │         1         11          0          1 │        13 
        GB │        62        154          0          1 │       221 
        GH │         0          0          0          0 │         2 
        GN │         0          0          0          0 │         1 
        GP │         0          0          0          0 │         1 
        HU │         0          0          0          0 │         7 
        IN │         0          0          0          0 │        49 
        IT │         2          0          0          7 │        10 
        KW │         0          0          0          0 │         1 
        KZ │         0          0          0          0 │         1 
        LB │         0          1          0          0 │         1 
        LT │         0          0          0          0 │        21 
        LV │         0          0          0          0 │        17 
        MD │         0          0          0          0 │         1 
        MN │         0          0          0          0 │         1 
        MU │         0          0          0          0 │         1 
        MY │         0          0          0          0 │         4 
        NG │         2          0          0          0 │        27 
        NL │         0          5          0          0 │         5 
        NP │         0          0          0          0 │         5 
        NZ │         0          1          0          0 │         2 
        PH │         0          0          0          0 │         8 
        PK │         0          0          0          0 │        13 
        PL │         1          0          0          0 │       137 
        PT │         0          0          0          1 │         1 
        RO │         1          0          0          0 │        16 
        RU │         0          0          0          0 │         2 
        SD │         0          0          0          0 │         1 
        SE │         0          0          1          0 │         1 
        UA │         0          0          0          0 │         2 
        US │         4          3          0          0 │        18 
        VE │         0          0          0          0 │         3 
        ZA │         1          4          0          0 │         8 
        ZW │         0          0          0          0 │         2 
───────────┼────────────────────────────────────────────┼──────────
     Total │        82        205          2         15 │       721 


Country of │
    birth, │                Ethnic groups
    father │     SEEur       EEur     NAmAus     MENACA │     Total
───────────┼────────────────────────────────────────────┼──────────
        77 │         0          0          0          0 │         1 
        99 │         6         10          5          7 │        59 
        AF │         0          0          0          1 │         1 
        AU │         0          0          1          0 │         1 
        BD │         0          0          0          0 │         7 
        BE │         0          0          0          0 │         1 
        BR │         0          0          0          0 │        14 
        CA │         0          0          0          0 │         1 
        CG │         0          0          0          0 │         1 
        CH │         0          0          0          0 │         1 
        CN │         0          0          0          0 │         3 
        CZ │         0          5          0          0 │         5 
        DE │         0          2          0          0 │        19 
        ES │         0          0          0          0 │         3 
        ET │         0          0          0          0 │         1 
        FR │         0          0          0          0 │        13 
        GB │         0          0          4          0 │       221 
        GH │         0          0          0          0 │         2 
        GN │         0          0          0          0 │         1 
        GP │         0          0          0          0 │         1 
        HU │         0          7          0          0 │         7 
        IN │         0          0          1          0 │        49 
        IT │         0          0          0          0 │        10 
        KW │         0          0          0          1 │         1 
        KZ │         0          1          0          0 │         1 
        LB │         0          0          0          0 │         1 
        LT │         0         21          0          0 │        21 
        LV │         0         17          0          0 │        17 
        MD │         1          0          0          0 │         1 
        MN │         0          0          0          0 │         1 
        MU │         0          0          0          0 │         1 
        MY │         0          0          0          0 │         4 
        NG │         0          0          0          0 │        27 
        NL │         0          0          0          0 │         5 
        NP │         0          0          0          0 │         5 
        NZ │         0          0          1          0 │         2 
        PH │         0          0          0          0 │         8 
        PK │         0          0          0          0 │        13 
        PL │         0        136          0          0 │       137 
        PT │         0          0          0          0 │         1 
        RO │        14          1          0          0 │        16 
        RU │         0          2          0          0 │         2 
        SD │         0          0          0          0 │         1 
        SE │         0          0          0          0 │         1 
        UA │         0          2          0          0 │         2 
        US │         0          0          9          0 │        18 
        VE │         0          0          0          0 │         3 
        ZA │         0          0          0          0 │         8 
        ZW │         0          0          1          0 │         2 
───────────┼────────────────────────────────────────────┼──────────
     Total │        21        204         22          9 │       721 


Country of │
    birth, │                Ethnic groups
    father │    SubSAf       SSEA         EA        LAm │     Total
───────────┼────────────────────────────────────────────┼──────────
        77 │         0          0          0          0 │         1 
        99 │         7          5          0          3 │        59 
        AF │         0          0          0          0 │         1 
        AU │         0          0          0          0 │         1 
        BD │         0          7          0          0 │         7 
        BE │         0          0          0          0 │         1 
        BR │         0          0          0         14 │        14 
        CA │         0          0          0          0 │         1 
        CG │         1          0          0          0 │         1 
        CH │         0          0          0          0 │         1 
        CN │         0          1          2          0 │         3 
        CZ │         0          0          0          0 │         5 
        DE │         0          0          0          0 │        19 
        ES │         0          0          0          0 │         3 
        ET │         1          0          0          0 │         1 
        FR │         0          0          0          0 │        13 
        GB │         0          0          0          0 │       221 
        GH │         2          0          0          0 │         2 
        GN │         1          0          0          0 │         1 
        GP │         0          0          0          0 │         1 
        HU │         0          0          0          0 │         7 
        IN │         0         48          0          0 │        49 
        IT │         0          0          0          1 │        10 
        KW │         0          0          0          0 │         1 
        KZ │         0          0          0          0 │         1 
        LB │         0          0          0          0 │         1 
        LT │         0          0          0          0 │        21 
        LV │         0          0          0          0 │        17 
        MD │         0          0          0          0 │         1 
        MN │         0          0          1          0 │         1 
        MU │         0          1          0          0 │         1 
        MY │         0          3          1          0 │         4 
        NG │        25          0          0          0 │        27 
        NL │         0          0          0          0 │         5 
        NP │         0          5          0          0 │         5 
        NZ │         0          0          0          0 │         2 
        PH │         0          0          0          8 │         8 
        PK │         0         13          0          0 │        13 
        PL │         0          0          0          0 │       137 
        PT │         0          0          0          0 │         1 
        RO │         0          0          0          0 │        16 
        RU │         0          0          0          0 │         2 
        SD │         1          0          0          0 │         1 
        SE │         0          0          0          0 │         1 
        UA │         0          0          0          0 │         2 
        US │         0          2          0          0 │        18 
        VE │         0          0          0          3 │         3 
        ZA │         3          0          0          0 │         8 
        ZW │         1          0          0          0 │         2 
───────────┼────────────────────────────────────────────┼──────────
     Total │        42         85          4         29 │       721 


Country of │   Ethnic
    birth, │   groups
    father │       Car │     Total
───────────┼───────────┼──────────
        77 │         0 │         1 
        99 │         0 │        59 
        AF │         0 │         1 
        AU │         0 │         1 
        BD │         0 │         7 
        BE │         0 │         1 
        BR │         0 │        14 
        CA │         0 │         1 
        CG │         0 │         1 
        CH │         0 │         1 
        CN │         0 │         3 
        CZ │         0 │         5 
        DE │         0 │        19 
        ES │         0 │         3 
        ET │         0 │         1 
        FR │         0 │        13 
        GB │         0 │       221 
        GH │         0 │         2 
        GN │         0 │         1 
        GP │         1 │         1 
        HU │         0 │         7 
        IN │         0 │        49 
        IT │         0 │        10 
        KW │         0 │         1 
        KZ │         0 │         1 
        LB │         0 │         1 
        LT │         0 │        21 
        LV │         0 │        17 
        MD │         0 │         1 
        MN │         0 │         1 
        MU │         0 │         1 
        MY │         0 │         4 
        NG │         0 │        27 
        NL │         0 │         5 
        NP │         0 │         5 
        NZ │         0 │         2 
        PH │         0 │         8 
        PK │         0 │        13 
        PL │         0 │       137 
        PT │         0 │         1 
        RO │         0 │        16 
        RU │         0 │         2 
        SD │         0 │         1 
        SE │         0 │         1 
        UA │         0 │         2 
        US │         0 │        18 
        VE │         0 │         3 
        ZA │         0 │         8 
        ZW │         0 │         2 
───────────┼───────────┼──────────
     Total │         1 │       721
```

For Ireland we can see that there is a substantial migration between the UK and Ireland in both directions, i.e. not only Irish people living in the UK, but also substantial numbers of people in Ireland who were born in Britain with non-Irish ancestry (most likely British, n=154). The large numbers of autochthonous with GB place of birth could easily represent return migration of Irish people from GB (n=98). There was extensive return migration after the ‘Celtic tiger’ boom after Ireland joined the EU (which ended with the financial crash of 2008). We also find that some people born in Australia and the US report exclusively Irish ancestry, which makes sense. None of the other groups of respondents born abroad report to have Irish ancestry except isolated cases.

## France

```
. tab coball anctryall2r if cntry=="fr"

    country of birth, │
 classified in ESCEG, │
     of respondent or │          Ethnic groups
     mother or father │   Autocht       WEur       NEur │     Total
──────────────────────┼─────────────────────────────────┼──────────
0. no migration backg │     2,643         83          0 │     3,007 
     1. West European │        36         60          1 │       108 
    2. North European │         1          0          2 │         3 
    3. South European │        37          0          0 │       230 
4. South-East Europea │         6          0          0 │        14 
     5. East European │        16          3          0 │        36 
6. North American and │         8          1          0 │         9 
7. MENA and Central A │       101          3          0 │       358 
8. Sub-Saharan Africa │        29          0          0 │        84 
9. South and South-Ea │         7          0          0 │        20 
       10. East Asian │         3          0          0 │         5 
   11. Latin American │         5          1          0 │        18 
        12. Caribbean │         3          0          0 │        11 
──────────────────────┼─────────────────────────────────┼──────────
                Total │     2,895        151          3 │     3,903 


    country of birth, │
 classified in ESCEG, │
     of respondent or │          Ethnic groups
     mother or father │      SEur      SEEur       EEur │     Total
──────────────────────┼─────────────────────────────────┼──────────
0. no migration backg │       196          2         15 │     3,007 
     1. West European │         7          1          2 │       108 
    2. North European │         0          0          0 │         3 
    3. South European │       187          0          0 │       230 
4. South-East Europea │         0          7          0 │        14 
     5. East European │         0          0         17 │        36 
6. North American and │         0          0          0 │         9 
7. MENA and Central A │        31          0          0 │       358 
8. Sub-Saharan Africa │         5          0          0 │        84 
9. South and South-Ea │         0          0          0 │        20 
       10. East Asian │         0          0          0 │         5 
   11. Latin American │         8          0          0 │        18 
        12. Caribbean │         0          0          0 │        11 
──────────────────────┼─────────────────────────────────┼──────────
                Total │       434         10         34 │     3,903 


    country of birth, │
 classified in ESCEG, │
     of respondent or │          Ethnic groups
     mother or father │    NAmAus     MENACA     SubSAf │     Total
──────────────────────┼─────────────────────────────────┼──────────
0. no migration backg │         1         20          6 │     3,007 
     1. West European │         1          0          0 │       108 
    2. North European │         0          0          0 │         3 
    3. South European │         2          2          2 │       230 
4. South-East Europea │         0          1          0 │        14 
     5. East European │         0          0          0 │        36 
6. North American and │         0          0          0 │         9 
7. MENA and Central A │         3        215          0 │       358 
8. Sub-Saharan Africa │         1          1         40 │        84 
9. South and South-Ea │         0          0          0 │        20 
       10. East Asian │         0          0          0 │         5 
   11. Latin American │         0          0          0 │        18 
        12. Caribbean │         0          0          0 │        11 
──────────────────────┼─────────────────────────────────┼──────────
                Total │         8        239         48 │     3,903 


    country of birth, │
 classified in ESCEG, │
     of respondent or │          Ethnic groups
     mother or father │      SSEA         EA        LAm │     Total
──────────────────────┼─────────────────────────────────┼──────────
0. no migration backg │         3          2          0 │     3,007 
     1. West European │         0          0          0 │       108 
    2. North European │         0          0          0 │         3 
    3. South European │         0          0          0 │       230 
4. South-East Europea │         0          0          0 │        14 
     5. East European │         0          0          0 │        36 
6. North American and │         0          0          0 │         9 
7. MENA and Central A │         3          0          0 │       358 
8. Sub-Saharan Africa │         1          0          0 │        84 
9. South and South-Ea │        11          2          0 │        20 
       10. East Asian │         0          2          0 │         5 
   11. Latin American │         0          0          4 │        18 
        12. Caribbean │         0          0          0 │        11 
──────────────────────┼─────────────────────────────────┼──────────
                Total │        18          6          4 │     3,903 


    country of birth, │
 classified in ESCEG, │   Ethnic
     of respondent or │   groups
     mother or father │       Car │     Total
──────────────────────┼───────────┼──────────
0. no migration backg │        36 │     3,007 
     1. West European │         0 │       108 
    2. North European │         0 │         3 
    3. South European │         0 │       230 
4. South-East Europea │         0 │        14 
     5. East European │         0 │        36 
6. North American and │         0 │         9 
7. MENA and Central A │         2 │       358 
8. Sub-Saharan Africa │         7 │        84 
9. South and South-Ea │         0 │        20 
       10. East Asian │         0 │         5 
   11. Latin American │         0 │        18 
        12. Caribbean │         8 │        11 
──────────────────────┼───────────┼──────────
                Total │        53 │     3,903
```

In France, we find some similarities to the UK situation. Out of 108 respondents reporting a West European country as a country of birth, 36 report French ancestry only. Again this may partly be due to French troops having children in post-war Germany, and partly to cross-border labor markets. Out of 230 with a country of birth in Southern Europe, 35 report French ancestry exclusively. Given France borders with several Southern European countries, and the languages are related, there may be French people working in Southern European countries and having children there. 356 respondents in France report a MENA or Central Asian country as a country of birth. Out of these, 94 report French ancestry only. Given the presence of the French in many of these countries until the middle of the 20th century, this may not be implausible and point to an important weakness of the country of birth measure, which mis-identify ‘returning’ children of colonial settlers as immigrants. We find a similar situation for respondents reporting Sub-Saharan Africa as a country of birth. Out of the 11 with the Caribbean as a country of birth, 3 report exclusively French ancestry, and out of the 9 with North America/Australasia as a country of birth, 6 report an exclusively French ancestry, all of which is plausible.

```
. tab rcob anctryall2r if cntry=="fr" & rcob!="66"

Country of │                Ethnic groups
     birth │   Autocht       WEur       NEur       SEur │     Total
───────────┼────────────────────────────────────────────┼──────────
        AF │         0          0          0          0 │         1 
        AL │         1          0          0          0 │         1 
        AM │         0          0          0          0 │         1 
        AO │         1          0          0          0 │         1 
        AR │         2          0          0          0 │         3 
        AT │         1          0          0          0 │         1 
        BA │         0          0          0          0 │         1 
        BD │         1          0          0          0 │         1 
        BE │         1         11          0          0 │        12 
        BJ │         0          0          0          1 │         3 
        BR │         2          0          0          2 │         4 
        BY │         0          0          0          0 │         1 
        CA │         1          0          0          0 │         1 
        CD │         1          0          0          0 │         6 
        CG │         2          0          0          1 │         4 
        CH │         2          6          0          1 │         9 
        CI │         5          0          0          0 │         9 
        CL │         0          0          0          2 │         3 
        CM │         1          0          0          0 │         3 
        CN │         1          0          0          0 │         2 
        CO │         0          0          0          1 │         1 
        CV │         0          0          0          1 │         2 
        DE │         1          7          0          0 │         8 
        DK │         0          0          1          0 │         1 
        DZ │        20          1          0          8 │        82 
        EC │         0          0          0          1 │         1 
        EG │         1          0          0          0 │         2 
        ES │         1          0          0         10 │        11 
        FI │         0          0          1          0 │         1 
        FR │         0          0          0          1 │         1 
        GA │         1          0          0          0 │         1 
        GB │         0          9          1          1 │        11 
        GE │         0          0          0          0 │         1 
        GP │         0          0          0          0 │         1 
        GR │         0          0          0          1 │         1 
        IN │         0          0          0          0 │         1 
        IT │         0          0          0         11 │        13 
        KH │         1          0          0          0 │         1 
        KR │         2          0          0          0 │         2 
        LA │         0          0          0          0 │         1 
        LB │         0          0          0          0 │         1 
        LC │         0          0          0          0 │         1 
        LK │         1          0          0          0 │         1 
        LT │         0          0          0          0 │         1 
        LV │         0          1          0          0 │         1 
        MA │        16          0          0          2 │        58 
        MG │         3          0          0          0 │         6 
        ML │         0          0          0          0 │         3 
        MQ │         1          0          0          0 │         4 
        MU │         0          0          0          0 │         3 
        MX │         0          0          0          0 │         1 
        NC │         1          0          0          0 │         1 
        NE │         0          0          0          0 │         1 
        PE │         1          0          0          1 │         2 
        PF │         1          0          0          0 │         1 
        PL │         1          0          0          0 │         3 
        PT │         1          0          0         29 │        32 
        RE │         0          0          0          0 │         2 
        RO │         0          0          0          0 │         4 
        RU │         0          0          0          0 │         3 
        RW │         0          0          0          0 │         1 
        SA │         0          0          0          0 │         1 
        SN │         2          0          0          0 │         8 
        SO │         0          0          0          0 │         1 
        ST │         0          0          0          1 │         1 
        TD │         1          0          0          0 │         3 
        TG │         0          0          0          0 │         1 
        TN │         4          1          0          4 │        25 
        TR │         0          0          0          0 │         4 
        UA │         0          0          0          0 │         1 
        US │         1          1          0          0 │         2 
        VN │         1          0          0          0 │         5 
        YT │         0          0          0          0 │         1 
        ZA │         1          0          0          0 │         1 
───────────┼────────────────────────────────────────────┼──────────
     Total │        85         37          3         79 │       394 


Country of │                Ethnic groups
     birth │     SEEur       EEur     NAmAus     MENACA │     Total
───────────┼────────────────────────────────────────────┼──────────
        AF │         0          0          0          1 │         1 
        AL │         0          0          0          0 │         1 
        AM │         0          0          0          1 │         1 
        AO │         0          0          0          0 │         1 
        AR │         0          0          0          0 │         3 
        AT │         0          0          0          0 │         1 
        BA │         1          0          0          0 │         1 
        BD │         0          0          0          0 │         1 
        BE │         0          0          0          0 │        12 
        BJ │         0          0          0          0 │         3 
        BR │         0          0          0          0 │         4 
        BY │         0          1          0          0 │         1 
        CA │         0          0          0          0 │         1 
        CD │         0          0          0          0 │         6 
        CG │         0          0          0          0 │         4 
        CH │         0          0          0          0 │         9 
        CI │         0          0          0          0 │         9 
        CL │         0          0          0          0 │         3 
        CM │         0          0          0          0 │         3 
        CN │         0          0          0          0 │         2 
        CO │         0          0          0          0 │         1 
        CV │         0          0          0          0 │         2 
        DE │         0          0          0          0 │         8 
        DK │         0          0          0          0 │         1 
        DZ │         0          0          1         52 │        82 
        EC │         0          0          0          0 │         1 
        EG │         0          0          0          1 │         2 
        ES │         0          0          0          0 │        11 
        FI │         0          0          0          0 │         1 
        FR │         0          0          0          0 │         1 
        GA │         0          0          0          0 │         1 
        GB │         0          0          0          0 │        11 
        GE │         0          0          0          1 │         1 
        GP │         0          0          0          0 │         1 
        GR │         0          0          0          0 │         1 
        IN │         0          0          0          0 │         1 
        IT │         0          0          1          1 │        13 
        KH │         0          0          0          0 │         1 
        KR │         0          0          0          0 │         2 
        LA │         0          0          0          0 │         1 
        LB │         0          0          0          1 │         1 
        LC │         0          0          0          0 │         1 
        LK │         0          0          0          0 │         1 
        LT │         0          1          0          0 │         1 
        LV │         0          0          0          0 │         1 
        MA │         0          0          0         40 │        58 
        MG │         0          0          0          0 │         6 
        ML │         0          0          0          0 │         3 
        MQ │         0          0          0          0 │         4 
        MU │         0          0          0          0 │         3 
        MX │         0          0          0          0 │         1 
        NC │         0          0          0          0 │         1 
        NE │         0          0          0          0 │         1 
        PE │         0          0          0          0 │         2 
        PF │         0          0          0          0 │         1 
        PL │         0          2          0          0 │         3 
        PT │         0          0          1          0 │        32 
        RE │         0          0          0          0 │         2 
        RO │         4          0          0          0 │         4 
        RU │         0          3          0          0 │         3 
        RW │         0          0          0          0 │         1 
        SA │         0          0          0          1 │         1 
        SN │         0          0          0          0 │         8 
        SO │         0          0          0          0 │         1 
        ST │         0          0          0          0 │         1 
        TD │         0          0          0          0 │         3 
        TG │         0          0          0          0 │         1 
        TN │         0          0          1         15 │        25 
        TR │         0          0          0          4 │         4 
        UA │         0          1          0          0 │         1 
        US │         0          0          0          0 │         2 
        VN │         0          0          0          0 │         5 
        YT │         0          0          1          0 │         1 
        ZA │         0          0          0          0 │         1 
───────────┼────────────────────────────────────────────┼──────────
     Total │         5          8          5        118 │       394 


Country of │                Ethnic groups
     birth │    SubSAf       SSEA         EA        LAm │     Total
───────────┼────────────────────────────────────────────┼──────────
        AF │         0          0          0          0 │         1 
        AL │         0          0          0          0 │         1 
        AM │         0          0          0          0 │         1 
        AO │         0          0          0          0 │         1 
        AR │         0          0          0          1 │         3 
        AT │         0          0          0          0 │         1 
        BA │         0          0          0          0 │         1 
        BD │         0          0          0          0 │         1 
        BE │         0          0          0          0 │        12 
        BJ │         2          0          0          0 │         3 
        BR │         0          0          0          0 │         4 
        BY │         0          0          0          0 │         1 
        CA │         0          0          0          0 │         1 
        CD │         5          0          0          0 │         6 
        CG │         1          0          0          0 │         4 
        CH │         0          0          0          0 │         9 
        CI │         4          0          0          0 │         9 
        CL │         0          0          0          1 │         3 
        CM │         2          0          0          0 │         3 
        CN │         0          0          1          0 │         2 
        CO │         0          0          0          0 │         1 
        CV │         1          0          0          0 │         2 
        DE │         0          0          0          0 │         8 
        DK │         0          0          0          0 │         1 
        DZ │         0          0          0          0 │        82 
        EC │         0          0          0          0 │         1 
        EG │         0          0          0          0 │         2 
        ES │         0          0          0          0 │        11 
        FI │         0          0          0          0 │         1 
        FR │         0          0          0          0 │         1 
        GA │         0          0          0          0 │         1 
        GB │         0          0          0          0 │        11 
        GE │         0          0          0          0 │         1 
        GP │         0          0          0          0 │         1 
        GR │         0          0          0          0 │         1 
        IN │         0          1          0          0 │         1 
        IT │         0          0          0          0 │        13 
        KH │         0          0          0          0 │         1 
        KR │         0          0          0          0 │         2 
        LA │         0          0          1          0 │         1 
        LB │         0          0          0          0 │         1 
        LC │         0          0          0          0 │         1 
        LK │         0          0          0          0 │         1 
        LT │         0          0          0          0 │         1 
        LV │         0          0          0          0 │         1 
        MA │         0          0          0          0 │        58 
        MG │         2          1          0          0 │         6 
        ML │         3          0          0          0 │         3 
        MQ │         0          0          0          0 │         4 
        MU │         0          2          0          0 │         3 
        MX │         0          0          0          1 │         1 
        NC │         0          0          0          0 │         1 
        NE │         1          0          0          0 │         1 
        PE │         0          0          0          0 │         2 
        PF │         0          0          0          0 │         1 
        PL │         0          0          0          0 │         3 
        PT │         1          0          0          0 │        32 
        RE │         0          0          0          0 │         2 
        RO │         0          0          0          0 │         4 
        RU │         0          0          0          0 │         3 
        RW │         0          0          0          0 │         1 
        SA │         0          0          0          0 │         1 
        SN │         6          0          0          0 │         8 
        SO │         1          0          0          0 │         1 
        ST │         0          0          0          0 │         1 
        TD │         2          0          0          0 │         3 
        TG │         1          0          0          0 │         1 
        TN │         0          0          0          0 │        25 
        TR │         0          0          0          0 │         4 
        UA │         0          0          0          0 │         1 
        US │         0          0          0          0 │         2 
        VN │         0          4          0          0 │         5 
        YT │         0          0          0          0 │         1 
        ZA │         0          0          0          0 │         1 
───────────┼────────────────────────────────────────────┼──────────
     Total │        32          8          2          3 │       394 


           │   Ethnic
Country of │   groups
     birth │       Car │     Total
───────────┼───────────┼──────────
        AF │         0 │         1 
        AL │         0 │         1 
        AM │         0 │         1 
        AO │         0 │         1 
        AR │         0 │         3 
        AT │         0 │         1 
        BA │         0 │         1 
        BD │         0 │         1 
        BE │         0 │        12 
        BJ │         0 │         3 
        BR │         0 │         4 
        BY │         0 │         1 
        CA │         0 │         1 
        CD │         0 │         6 
        CG │         0 │         4 
        CH │         0 │         9 
        CI │         0 │         9 
        CL │         0 │         3 
        CM │         0 │         3 
        CN │         0 │         2 
        CO │         0 │         1 
        CV │         0 │         2 
        DE │         0 │         8 
        DK │         0 │         1 
        DZ │         0 │        82 
        EC │         0 │         1 
        EG │         0 │         2 
        ES │         0 │        11 
        FI │         0 │         1 
        FR │         0 │         1 
        GA │         0 │         1 
        GB │         0 │        11 
        GE │         0 │         1 
        GP │         1 │         1 
        GR │         0 │         1 
        IN │         0 │         1 
        IT │         0 │        13 
        KH │         0 │         1 
        KR │         0 │         2 
        LA │         0 │         1 
        LB │         0 │         1 
        LC │         1 │         1 
        LK │         0 │         1 
        LT │         0 │         1 
        LV │         0 │         1 
        MA │         0 │        58 
        MG │         0 │         6 
        ML │         0 │         3 
        MQ │         3 │         4 
        MU │         1 │         3 
        MX │         0 │         1 
        NC │         0 │         1 
        NE │         0 │         1 
        PE │         0 │         2 
        PF │         0 │         1 
        PL │         0 │         3 
        PT │         0 │        32 
        RE │         2 │         2 
        RO │         0 │         4 
        RU │         0 │         3 
        RW │         1 │         1 
        SA │         0 │         1 
        SN │         0 │         8 
        SO │         0 │         1 
        ST │         0 │         1 
        TD │         0 │         3 
        TG │         0 │         1 
        TN │         0 │        25 
        TR │         0 │         4 
        UA │         0 │         1 
        US │         0 │         2 
        VN │         0 │         5 
        YT │         0 │         1 
        ZA │         0 │         1 
───────────┼───────────┼──────────
     Total │         9 │       394
```

Looking at the specific countries of birth of the respondents and their reported ancestry, we firstly note that in contrast to the UK, there is no problem of missing data - maybe it’s rather that the UK did not code the data well (countries are post-coded from verbatim information) or information was suppressed for anonymization. Anyway, we’ll only look at countries where more than 5 respondents were born to simplify the task. Those born in Belgium (BE), Germany (DE), Switzerland (CH) or the UK (GB) almost all report West European, not French, ancestry. Thus, there is no evidence of children born to French troops in Germany during the occupation after WWII, but rather of a West European labor market. Moving to Southern European countries of birth, almost all born in Spain (ES), Italy (IT) or Portugal (PT) report South European ancestry. Looking at Africa, those born in Congo (CD), Côte d’Ivoire (CI) and Senegal (SN) mostly report Sub-Saharan ancestry but around a quarteralso exclusively French ancestry. The situation is similar for those born in Algeria (DZ), Tunisia (TN) and Morocco (MA), reporting mostly MENA ancestry, but also exclusively French ancestry in about a quarter of cases. Some of the respondents born in North Africa also report South European ancestry.

```
. tab mcob anctryall2r if cntry=="fr" & mcob!="66"

Country of │
    birth, │                Ethnic groups
    mother │   Autocht       WEur       NEur       SEur │     Total
───────────┼────────────────────────────────────────────┼──────────
        AD │         1          0          0          0 │         1 
        AO │         1          0          0          0 │         1 
        AR │         0          0          0          1 │         2 
        AT │         3          0          0          0 │         3 
        AU │         1          0          0          0 │         1 
        BA │         1          0          0          0 │         2 
        BD │         1          0          0          0 │         1 
        BE │         8         19          0          0 │        29 
        BJ │         0          0          0          0 │         2 
        BR │         0          0          0          2 │         2 
        BY │         0          0          0          0 │         1 
        CD │         2          0          0          0 │         6 
        CF │         1          0          0          0 │         1 
        CG │         0          0          0          1 │         2 
        CH │         1          5          0          0 │         6 
        CI │         3          0          0          0 │         8 
        CL │         0          0          0          1 │         2 
        CM │         1          0          0          0 │         4 
        CN │         0          0          0          0 │         1 
        CV │         0          0          0          1 │         2 
        CZ │         1          1          0          0 │         2 
        DE │         8         10          0          1 │        21 
        DK │         0          0          1          0 │         1 
        DZ │        33          0          0          9 │       117 
        EC │         0          0          0          1 │         1 
        EG │         0          0          0          0 │         2 
        ES │         7          0          0         34 │        42 
        FI │         1          0          1          0 │         2 
        GB │         0          6          1          2 │         9 
        GE │         0          0          0          0 │         1 
        GP │         1          0          0          0 │         3 
        GR │         0          1          0          2 │         3 
        GW │         1          0          0          0 │         1 
        HT │         0          0          0          0 │         1 
        HU │         0          0          0          0 │         2 
        IN │         0          1          0          0 │         2 
        IR │         0          0          0          0 │         1 
        IT │         9          0          0         57 │        68 
        JM │         0          1          0          0 │         1 
        KE │         0          1          0          0 │         1 
        KH │         1          0          0          0 │         2 
        KM │         1          0          0          0 │         1 
        LA │         0          0          0          0 │         2 
        LB │         0          1          0          0 │         2 
        LC │         0          0          0          0 │         1 
        LK │         1          0          0          0 │         1 
        LT │         0          0          0          0 │         1 
        LV │         0          1          0          0 │         1 
        LY │         0          0          0          0 │         1 
        MA │        12          0          0          4 │        81 
        MG │         4          0          0          0 │         8 
        ML │         0          0          0          0 │         5 
        MQ │         1          0          0          0 │         4 
        MU │         1          0          0          0 │         6 
        MZ │         0          0          0          0 │         1 
        NE │         0          0          0          0 │         1 
        NL │         0          1          0          0 │         2 
        PE │         1          0          0          1 │         3 
        PK │         0          0          0          0 │         1 
        PL │         8          1          0          0 │        14 
        PR │         0          0          0          1 │         1 
        PT │         3          0          0         44 │        48 
        RE │         0          0          0          0 │         3 
        RO │         1          1          0          0 │         6 
        RS │         0          0          0          0 │         1 
        RU │         0          0          0          0 │         5 
        RW │         0          0          0          0 │         2 
        SG │         0          0          0          0 │         1 
        SK │         0          0          0          0 │         1 
        SM │         0          0          0          1 │         1 
        SN │         0          0          0          0 │        10 
        SO │         0          0          0          0 │         1 
        ST │         0          0          0          1 │         1 
        TD │         0          0          0          0 │         2 
        TN │         8          1          0          4 │        33 
        TR │         1          0          0          0 │        13 
        UA │         2          0          0          0 │         4 
        VN │         1          0          0          0 │         6 
        YT │         0          0          0          0 │         1 
───────────┼────────────────────────────────────────────┼──────────
     Total │       131         51          3        168 │       636 


Country of │
    birth, │                Ethnic groups
    mother │     SEEur       EEur     NAmAus     MENACA │     Total
───────────┼────────────────────────────────────────────┼──────────
        AD │         0          0          0          0 │         1 
        AO │         0          0          0          0 │         1 
        AR │         0          0          0          0 │         2 
        AT │         0          0          0          0 │         3 
        AU │         0          0          0          0 │         1 
        BA │         1          0          0          0 │         2 
        BD │         0          0          0          0 │         1 
        BE │         1          0          0          1 │        29 
        BJ │         0          0          0          0 │         2 
        BR │         0          0          0          0 │         2 
        BY │         0          1          0          0 │         1 
        CD │         0          0          0          0 │         6 
        CF │         0          0          0          0 │         1 
        CG │         0          0          0          0 │         2 
        CH │         0          0          0          0 │         6 
        CI │         0          0          0          0 │         8 
        CL │         0          0          0          0 │         2 
        CM │         0          0          0          0 │         4 
        CN │         0          0          0          0 │         1 
        CV │         0          0          0          0 │         2 
        CZ │         0          0          0          0 │         2 
        DE │         0          2          0          0 │        21 
        DK │         0          0          0          0 │         1 
        DZ │         0          0          0         75 │       117 
        EC │         0          0          0          0 │         1 
        EG │         0          0          0          2 │         2 
        ES │         0          0          1          0 │        42 
        FI │         0          0          0          0 │         2 
        GB │         0          0          0          0 │         9 
        GE │         0          0          0          1 │         1 
        GP │         0          0          0          0 │         3 
        GR │         0          0          0          0 │         3 
        GW │         0          0          0          0 │         1 
        HT │         0          0          0          0 │         1 
        HU │         0          2          0          0 │         2 
        IN │         0          0          0          0 │         2 
        IR │         0          0          0          1 │         1 
        IT │         0          0          0          1 │        68 
        JM │         0          0          0          0 │         1 
        KE │         0          0          0          0 │         1 
        KH │         0          0          0          0 │         2 
        KM │         0          0          0          0 │         1 
        LA │         0          0          0          0 │         2 
        LB │         0          0          0          1 │         2 
        LC │         0          0          0          0 │         1 
        LK │         0          0          0          0 │         1 
        LT │         0          1          0          0 │         1 
        LV │         0          0          0          0 │         1 
        LY │         0          0          1          0 │         1 
        MA │         0          0          0         65 │        81 
        MG │         0          0          0          0 │         8 
        ML │         0          0          0          0 │         5 
        MQ │         0          0          0          0 │         4 
        MU │         0          0          1          0 │         6 
        MZ │         0          0          0          0 │         1 
        NE │         0          0          0          0 │         1 
        NL │         0          0          1          0 │         2 
        PE │         0          0          0          0 │         3 
        PK │         0          0          0          0 │         1 
        PL │         0          5          0          0 │        14 
        PR │         0          0          0          0 │         1 
        PT │         0          0          1          0 │        48 
        RE │         0          0          0          0 │         3 
        RO │         4          0          0          0 │         6 
        RS │         1          0          0          0 │         1 
        RU │         0          4          0          1 │         5 
        RW │         0          0          0          0 │         2 
        SG │         0          0          0          0 │         1 
        SK │         0          1          0          0 │         1 
        SM │         0          0          0          0 │         1 
        SN │         0          0          0          0 │        10 
        SO │         0          0          0          0 │         1 
        ST │         0          0          0          0 │         1 
        TD │         0          0          0          0 │         2 
        TN │         0          0          0         20 │        33 
        TR │         0          0          0         12 │        13 
        UA │         0          2          0          0 │         4 
        VN │         0          0          0          0 │         6 
        YT │         0          0          1          0 │         1 
───────────┼────────────────────────────────────────────┼──────────
     Total │         7         18          6        180 │       636 


Country of │
    birth, │                Ethnic groups
    mother │    SubSAf       SSEA         EA        LAm │     Total
───────────┼────────────────────────────────────────────┼──────────
        AD │         0          0          0          0 │         1 
        AO │         0          0          0          0 │         1 
        AR │         0          0          0          1 │         2 
        AT │         0          0          0          0 │         3 
        AU │         0          0          0          0 │         1 
        BA │         0          0          0          0 │         2 
        BD │         0          0          0          0 │         1 
        BE │         0          0          0          0 │        29 
        BJ │         2          0          0          0 │         2 
        BR │         0          0          0          0 │         2 
        BY │         0          0          0          0 │         1 
        CD │         4          0          0          0 │         6 
        CF │         0          0          0          0 │         1 
        CG │         1          0          0          0 │         2 
        CH │         0          0          0          0 │         6 
        CI │         5          0          0          0 │         8 
        CL │         0          0          0          1 │         2 
        CM │         3          0          0          0 │         4 
        CN │         0          0          1          0 │         1 
        CV │         1          0          0          0 │         2 
        CZ │         0          0          0          0 │         2 
        DE │         0          0          0          0 │        21 
        DK │         0          0          0          0 │         1 
        DZ │         0          0          0          0 │       117 
        EC │         0          0          0          0 │         1 
        EG │         0          0          0          0 │         2 
        ES │         0          0          0          0 │        42 
        FI │         0          0          0          0 │         2 
        GB │         0          0          0          0 │         9 
        GE │         0          0          0          0 │         1 
        GP │         0          0          0          0 │         3 
        GR │         0          0          0          0 │         3 
        GW │         0          0          0          0 │         1 
        HT │         0          0          0          0 │         1 
        HU │         0          0          0          0 │         2 
        IN │         0          1          0          0 │         2 
        IR │         0          0          0          0 │         1 
        IT │         1          0          0          0 │        68 
        JM │         0          0          0          0 │         1 
        KE │         0          0          0          0 │         1 
        KH │         0          0          1          0 │         2 
        KM │         0          0          0          0 │         1 
        LA │         0          1          1          0 │         2 
        LB │         0          0          0          0 │         2 
        LC │         0          0          0          0 │         1 
        LK │         0          0          0          0 │         1 
        LT │         0          0          0          0 │         1 
        LV │         0          0          0          0 │         1 
        LY │         0          0          0          0 │         1 
        MA │         0          0          0          0 │        81 
        MG │         3          0          0          0 │         8 
        ML │         5          0          0          0 │         5 
        MQ │         0          0          0          0 │         4 
        MU │         0          2          0          0 │         6 
        MZ │         0          1          0          0 │         1 
        NE │         1          0          0          0 │         1 
        NL │         0          0          0          0 │         2 
        PE │         0          0          0          1 │         3 
        PK │         0          1          0          0 │         1 
        PL │         0          0          0          0 │        14 
        PR │         0          0          0          0 │         1 
        PT │         0          0          0          0 │        48 
        RE │         0          0          0          0 │         3 
        RO │         0          0          0          0 │         6 
        RS │         0          0          0          0 │         1 
        RU │         0          0          0          0 │         5 
        RW │         1          0          0          0 │         2 
        SG │         0          0          1          0 │         1 
        SK │         0          0          0          0 │         1 
        SM │         0          0          0          0 │         1 
        SN │         9          0          0          0 │        10 
        SO │         1          0          0          0 │         1 
        ST │         0          0          0          0 │         1 
        TD │         2          0          0          0 │         2 
        TN │         0          0          0          0 │        33 
        TR │         0          0          0          0 │        13 
        UA │         0          0          0          0 │         4 
        VN │         0          5          0          0 │         6 
        YT │         0          0          0          0 │         1 
───────────┼────────────────────────────────────────────┼──────────
     Total │        39         11          4          3 │       636 


Country of │   Ethnic
    birth, │   groups
    mother │       Car │     Total
───────────┼───────────┼──────────
        AD │         0 │         1 
        AO │         0 │         1 
        AR │         0 │         2 
        AT │         0 │         3 
        AU │         0 │         1 
        BA │         0 │         2 
        BD │         0 │         1 
        BE │         0 │        29 
        BJ │         0 │         2 
        BR │         0 │         2 
        BY │         0 │         1 
        CD │         0 │         6 
        CF │         0 │         1 
        CG │         0 │         2 
        CH │         0 │         6 
        CI │         0 │         8 
        CL │         0 │         2 
        CM │         0 │         4 
        CN │         0 │         1 
        CV │         0 │         2 
        CZ │         0 │         2 
        DE │         0 │        21 
        DK │         0 │         1 
        DZ │         0 │       117 
        EC │         0 │         1 
        EG │         0 │         2 
        ES │         0 │        42 
        FI │         0 │         2 
        GB │         0 │         9 
        GE │         0 │         1 
        GP │         2 │         3 
        GR │         0 │         3 
        GW │         0 │         1 
        HT │         1 │         1 
        HU │         0 │         2 
        IN │         0 │         2 
        IR │         0 │         1 
        IT │         0 │        68 
        JM │         0 │         1 
        KE │         0 │         1 
        KH │         0 │         2 
        KM │         0 │         1 
        LA │         0 │         2 
        LB │         0 │         2 
        LC │         1 │         1 
        LK │         0 │         1 
        LT │         0 │         1 
        LV │         0 │         1 
        LY │         0 │         1 
        MA │         0 │        81 
        MG │         1 │         8 
        ML │         0 │         5 
        MQ │         3 │         4 
        MU │         2 │         6 
        MZ │         0 │         1 
        NE │         0 │         1 
        NL │         0 │         2 
        PE │         0 │         3 
        PK │         0 │         1 
        PL │         0 │        14 
        PR │         0 │         1 
        PT │         0 │        48 
        RE │         3 │         3 
        RO │         0 │         6 
        RS │         0 │         1 
        RU │         0 │         5 
        RW │         1 │         2 
        SG │         0 │         1 
        SK │         0 │         1 
        SM │         0 │         1 
        SN │         1 │        10 
        SO │         0 │         1 
        ST │         0 │         1 
        TD │         0 │         2 
        TN │         0 │        33 
        TR │         0 │        13 
        UA │         0 │         4 
        VN │         0 │         6 
        YT │         0 │         1 
───────────┼───────────┼──────────
     Total │        15 │       636 

. tab fcob anctryall2r if cntry=="fr" & fcob!="66"

Country of │
    birth, │                Ethnic groups
    father │   Autocht       WEur       NEur       SEur │     Total
───────────┼────────────────────────────────────────────┼──────────
        AF │         0          0          0          0 │         1 
        AL │         1          0          0          0 │         1 
        AM │         0          0          0          0 │         1 
        AO │         1          0          0          0 │         1 
        AR │         0          0          0          0 │         1 
        AT │         2          0          0          0 │         3 
        BA │         1          0          0          0 │         3 
        BD │         1          0          0          0 │         1 
        BE │         8         21          0          1 │        30 
        BJ │         1          0          0          0 │         2 
        BR │         0          0          0          2 │         2 
        BY │         0          0          0          0 │         1 
        BZ │         0          1          0          0 │         1 
        CA │         1          0          0          0 │         1 
        CD │         1          0          0          1 │         6 
        CF │         1          0          0          0 │         1 
        CG │         1          0          0          1 │         3 
        CH │         1          4          0          2 │         7 
        CI │         2          0          0          2 │         9 
        CL │         0          0          0          0 │         1 
        CM │         2          0          0          0 │         6 
        CN │         0          0          0          0 │         1 
        CV │         0          0          0          1 │         2 
        CZ │         0          0          0          0 │         1 
        DE │         4         10          0          0 │        14 
        DK │         0          0          1          0 │         1 
        DZ │        38          0          0         10 │       137 
        EC │         0          0          0          1 │         1 
        EG │         2          0          0          0 │         5 
        ES │        12          0          0         37 │        49 
        FI │         0          0          1          0 │         1 
        GB │         2          8          1          0 │        12 
        GE │         0          0          0          0 │         1 
        GP │         2          0          0          0 │         4 
        GR │         1          0          0          1 │         2 
        HR │         2          0          0          0 │         3 
        HU │         0          0          0          0 │         1 
        IE │         0          2          0          0 │         2 
        IL │         1          0          0          1 │         2 
        IN │         0          0          0          0 │         1 
        IT │        13          0          0         70 │        86 
        JM │         0          1          0          0 │         1 
        KH │         1          0          0          0 │         3 
        KM │         1          0          0          0 │         2 
        LA │         0          0          0          0 │         2 
        LB │         1          0          0          0 │         1 
        LC │         0          0          0          0 │         1 
        LK │         1          0          0          0 │         1 
        LT │         0          0          0          0 │         1 
        LV │         0          1          0          0 │         1 
        MA │         9          0          0          4 │        79 
        MG │         3          0          0          1 │         8 
        MK │         0          0          0          0 │         1 
        ML │         0          0          0          0 │         4 
        MQ │         1          0          0          0 │         6 
        MU │         1          0          0          0 │         6 
        MX │         0          0          0          0 │         1 
        NE │         0          0          0          0 │         1 
        NL │         1          0          0          0 │         2 
        PE │         1          0          0          1 │         2 
        PF │         1          0          0          0 │         1 
        PK │         1          0          0          0 │         2 
        PL │         9          2          0          0 │        15 
        PT │         5          0          0         50 │        56 
        RE │         1          0          0          0 │         5 
        RO │         1          0          0          0 │         5 
        RU │         1          0          0          0 │         5 
        RW │         0          0          0          0 │         2 
        SI │         1          0          0          0 │         1 
        SN │         0          0          0          0 │        10 
        SO │         0          0          0          0 │         1 
        ST │         0          0          0          1 │         1 
        SY │         1          0          0          0 │         1 
        TD │         0          0          0          0 │         2 
        TG │         0          0          0          0 │         1 
        TN │        10          1          0          4 │        42 
        TR │         2          0          0          1 │        17 
        UA │         1          0          0          0 │         2 
        US │         2          0          0          0 │         2 
        VN │         2          0          0          0 │         6 
        YT │         0          0          0          0 │         1 
───────────┼────────────────────────────────────────────┼──────────
     Total │       159         51          3        192 │       709 


Country of │
    birth, │                Ethnic groups
    father │     SEEur       EEur     NAmAus     MENACA │     Total
───────────┼────────────────────────────────────────────┼──────────
        AF │         0          0          0          1 │         1 
        AL │         0          0          0          0 │         1 
        AM │         0          0          0          1 │         1 
        AO │         0          0          0          0 │         1 
        AR │         0          0          0          0 │         1 
        AT │         0          1          0          0 │         3 
        BA │         1          0          0          1 │         3 
        BD │         0          0          0          0 │         1 
        BE │         0          0          0          0 │        30 
        BJ │         0          0          0          0 │         2 
        BR │         0          0          0          0 │         2 
        BY │         0          1          0          0 │         1 
        BZ │         0          0          0          0 │         1 
        CA │         0          0          0          0 │         1 
        CD │         0          0          0          0 │         6 
        CF │         0          0          0          0 │         1 
        CG │         0          0          0          0 │         3 
        CH │         0          0          0          0 │         7 
        CI │         0          0          0          0 │         9 
        CL │         0          0          0          0 │         1 
        CM │         0          0          0          0 │         6 
        CN │         0          0          0          0 │         1 
        CV │         0          0          0          0 │         2 
        CZ │         0          1          0          0 │         1 
        DE │         0          0          0          0 │        14 
        DK │         0          0          0          0 │         1 
        DZ │         0          0          1         88 │       137 
        EC │         0          0          0          0 │         1 
        EG │         0          0          0          3 │         5 
        ES │         0          0          0          0 │        49 
        FI │         0          0          0          0 │         1 
        GB │         0          0          0          0 │        12 
        GE │         0          0          0          1 │         1 
        GP │         0          0          0          0 │         4 
        GR │         0          0          0          0 │         2 
        HR │         1          0          0          0 │         3 
        HU │         0          1          0          0 │         1 
        IE │         0          0          0          0 │         2 
        IL │         0          0          0          0 │         2 
        IN │         0          0          0          0 │         1 
        IT │         0          0          2          0 │        86 
        JM │         0          0          0          0 │         1 
        KH │         0          0          0          0 │         3 
        KM │         0          0          0          0 │         2 
        LA │         0          0          0          0 │         2 
        LB │         0          0          0          0 │         1 
        LC │         0          0          0          0 │         1 
        LK │         0          0          0          0 │         1 
        LT │         0          1          0          0 │         1 
        LV │         0          0          0          0 │         1 
        MA │         0          0          0         66 │        79 
        MG │         0          0          0          0 │         8 
        MK │         1          0          0          0 │         1 
        ML │         0          0          0          0 │         4 
        MQ │         0          0          0          0 │         6 
        MU │         0          0          0          0 │         6 
        MX │         0          0          0          0 │         1 
        NE │         0          0          0          0 │         1 
        NL │         0          0          1          0 │         2 
        PE │         0          0          0          0 │         2 
        PF │         0          0          0          0 │         1 
        PK │         0          0          0          0 │         2 
        PL │         0          4          0          0 │        15 
        PT │         0          0          1          0 │        56 
        RE │         0          0          0          0 │         5 
        RO │         4          0          0          0 │         5 
        RU │         0          4          0          0 │         5 
        RW │         0          0          0          0 │         2 
        SI │         0          0          0          0 │         1 
        SN │         0          0          0          1 │        10 
        SO │         0          0          0          0 │         1 
        ST │         0          0          0          0 │         1 
        SY │         0          0          0          0 │         1 
        TD │         0          0          0          0 │         2 
        TG │         0          0          0          0 │         1 
        TN │         0          0          1         26 │        42 
        TR │         0          0          0         14 │        17 
        UA │         0          1          0          0 │         2 
        US │         0          0          0          0 │         2 
        VN │         0          0          0          0 │         6 
        YT │         0          0          1          0 │         1 
───────────┼────────────────────────────────────────────┼──────────
     Total │         7         14          7        202 │       709 


Country of │
    birth, │                Ethnic groups
    father │    SubSAf       SSEA         EA        LAm │     Total
───────────┼────────────────────────────────────────────┼──────────
        AF │         0          0          0          0 │         1 
        AL │         0          0          0          0 │         1 
        AM │         0          0          0          0 │         1 
        AO │         0          0          0          0 │         1 
        AR │         0          0          0          1 │         1 
        AT │         0          0          0          0 │         3 
        BA │         0          0          0          0 │         3 
        BD │         0          0          0          0 │         1 
        BE │         0          0          0          0 │        30 
        BJ │         1          0          0          0 │         2 
        BR │         0          0          0          0 │         2 
        BY │         0          0          0          0 │         1 
        BZ │         0          0          0          0 │         1 
        CA │         0          0          0          0 │         1 
        CD │         4          0          0          0 │         6 
        CF │         0          0          0          0 │         1 
        CG │         1          0          0          0 │         3 
        CH │         0          0          0          0 │         7 
        CI │         5          0          0          0 │         9 
        CL │         0          0          0          1 │         1 
        CM │         4          0          0          0 │         6 
        CN │         0          0          1          0 │         1 
        CV │         1          0          0          0 │         2 
        CZ │         0          0          0          0 │         1 
        DE │         0          0          0          0 │        14 
        DK │         0          0          0          0 │         1 
        DZ │         0          0          0          0 │       137 
        EC │         0          0          0          0 │         1 
        EG │         0          0          0          0 │         5 
        ES │         0          0          0          0 │        49 
        FI │         0          0          0          0 │         1 
        GB │         1          0          0          0 │        12 
        GE │         0          0          0          0 │         1 
        GP │         0          0          0          0 │         4 
        GR │         0          0          0          0 │         2 
        HR │         0          0          0          0 │         3 
        HU │         0          0          0          0 │         1 
        IE │         0          0          0          0 │         2 
        IL │         0          0          0          0 │         2 
        IN │         0          1          0          0 │         1 
        IT │         1          0          0          0 │        86 
        JM │         0          0          0          0 │         1 
        KH │         0          1          1          0 │         3 
        KM │         0          0          0          0 │         2 
        LA │         0          1          1          0 │         2 
        LB │         0          0          0          0 │         1 
        LC │         0          0          0          0 │         1 
        LK │         0          0          0          0 │         1 
        LT │         0          0          0          0 │         1 
        LV │         0          0          0          0 │         1 
        MA │         0          0          0          0 │        79 
        MG │         3          1          0          0 │         8 
        MK │         0          0          0          0 │         1 
        ML │         4          0          0          0 │         4 
        MQ │         0          0          0          0 │         6 
        MU │         0          3          0          0 │         6 
        MX │         0          0          0          1 │         1 
        NE │         1          0          0          0 │         1 
        NL │         0          0          0          0 │         2 
        PE │         0          0          0          0 │         2 
        PF │         0          0          0          0 │         1 
        PK │         0          1          0          0 │         2 
        PL │         0          0          0          0 │        15 
        PT │         0          0          0          0 │        56 
        RE │         0          0          0          0 │         5 
        RO │         0          0          0          0 │         5 
        RU │         0          0          0          0 │         5 
        RW │         1          0          0          0 │         2 
        SI │         0          0          0          0 │         1 
        SN │         9          0          0          0 │        10 
        SO │         1          0          0          0 │         1 
        ST │         0          0          0          0 │         1 
        SY │         0          0          0          0 │         1 
        TD │         2          0          0          0 │         2 
        TG │         1          0          0          0 │         1 
        TN │         0          0          0          0 │        42 
        TR │         0          0          0          0 │        17 
        UA │         0          0          0          0 │         2 
        US │         0          0          0          0 │         2 
        VN │         0          4          0          0 │         6 
        YT │         0          0          0          0 │         1 
───────────┼────────────────────────────────────────────┼──────────
     Total │        40         12          3          3 │       709 


Country of │   Ethnic
    birth, │   groups
    father │       Car │     Total
───────────┼───────────┼──────────
        AF │         0 │         1 
        AL │         0 │         1 
        AM │         0 │         1 
        AO │         0 │         1 
        AR │         0 │         1 
        AT │         0 │         3 
        BA │         0 │         3 
        BD │         0 │         1 
        BE │         0 │        30 
        BJ │         0 │         2 
        BR │         0 │         2 
        BY │         0 │         1 
        BZ │         0 │         1 
        CA │         0 │         1 
        CD │         0 │         6 
        CF │         0 │         1 
        CG │         0 │         3 
        CH │         0 │         7 
        CI │         0 │         9 
        CL │         0 │         1 
        CM │         0 │         6 
        CN │         0 │         1 
        CV │         0 │         2 
        CZ │         0 │         1 
        DE │         0 │        14 
        DK │         0 │         1 
        DZ │         0 │       137 
        EC │         0 │         1 
        EG │         0 │         5 
        ES │         0 │        49 
        FI │         0 │         1 
        GB │         0 │        12 
        GE │         0 │         1 
        GP │         2 │         4 
        GR │         0 │         2 
        HR │         0 │         3 
        HU │         0 │         1 
        IE │         0 │         2 
        IL │         0 │         2 
        IN │         0 │         1 
        IT │         0 │        86 
        JM │         0 │         1 
        KH │         0 │         3 
        KM │         1 │         2 
        LA │         0 │         2 
        LB │         0 │         1 
        LC │         1 │         1 
        LK │         0 │         1 
        LT │         0 │         1 
        LV │         0 │         1 
        MA │         0 │        79 
        MG │         0 │         8 
        MK │         0 │         1 
        ML │         0 │         4 
        MQ │         5 │         6 
        MU │         2 │         6 
        MX │         0 │         1 
        NE │         0 │         1 
        NL │         0 │         2 
        PE │         0 │         2 
        PF │         0 │         1 
        PK │         0 │         2 
        PL │         0 │        15 
        PT │         0 │        56 
        RE │         4 │         5 
        RO │         0 │         5 
        RU │         0 │         5 
        RW │         1 │         2 
        SI │         0 │         1 
        SN │         0 │        10 
        SO │         0 │         1 
        ST │         0 │         1 
        SY │         0 │         1 
        TD │         0 │         2 
        TG │         0 │         1 
        TN │         0 │        42 
        TR │         0 │        17 
        UA │         0 │         2 
        US │         0 │         2 
        VN │         0 │         6 
        YT │         0 │         1 
───────────┼───────────┼──────────
     Total │        16 │       709
```

Turning to the country of birth of mothers and fathers of respondents in France, 29 (30) respondents report that their mother (father) was born in Belgium. Out of these, 8 (8) at the same time report French ancestry only. So this would imply that their French mothers (fathers) were born in Belgium. Given Belgium is partly French-speaking and a neighbor of France, this is not implausible. The situation is not dissimilar for the other neighboring countries of France. Looking at Italy, while 68 (86) say their mother was born in Italy, 9 (13) report French ancestry only. Of the 21 (18) respondents reporting their mother (father) to be born in Germany, 8 (4) report exclusively French ancestry (we could look at their year of birth to help interpretation). Of the 7 (10) saying that their mother (father) was born in the UK, none (2) report exclusively French ancestry. The Spanish are a more important migrant group in France, many having fled from the Franco regime. Out of the 41 (48) respondents reporting that their mother (father) was born in Spain, only 7 (12) report exclusively French ancestry. A few of these may be Basque people (and we could check this if needed). Of those reporting parents born in Portugal, even fewer report French ancestry.

Turning to North Africa, amongst those 117 (134) reporting that their mother (father) was born in Algeria, 33 (35) report exclusively French ancestry. These would be descendants of the ‘pieds noirs’ leaving Algeria after the Algerian war in the 1960s. Since also people from Italy and Spain had settled in Algeria and acquired French citizenship in Algeria, it is not surprising that South-European ancestry is also mentioned by some people in France whose parents were born in Algeria. Out of the 79 (77) respondents saying that their mother (father) was born in Morocco, 11 (8) claim to have exclusively French ancestry. It is similar for respondents with parents born in Tunisia. So with respect to these groups, they are a mix of (‘re’-)migrating descendants of former (mostly French) settlers and North African migrants of North African descent.

Overall, these results look plausible, even though the number of people of French ancestry born abroad (or with parents born abroad) is quite high. I was surprised to see that of those 14 (15) respondents saying that their mother (father) was born in Poland, 5 (5) would claim French ancestry only since historically there have been many Polish immigrants in France, but not the other way round.

## Germany

It will be interesting to see what the situation looks like for Germany, where colonial conquests played a much smaller role, but an important diaspora had developed in Eastern Europe over the centuries and the two World Wars led to changing boundaries and large-scale migration.

```
. tab coball anctryall2r if cntry=="de"

    country of birth, │
 classified in ESCEG, │
     of respondent or │          Ethnic groups
     mother or father │   Autocht       WEur       NEur │     Total
──────────────────────┼─────────────────────────────────┼──────────
0. no migration backg │     4,573         25          7 │     4,692 
     1. West European │        42         71          0 │       122 
    2. North European │         7          0          6 │        13 
    3. South European │         7          0          0 │        77 
4. South-East Europea │        35          1          0 │       124 
     5. East European │       260          2          0 │       451 
6. North American and │         9          0          0 │        21 
7. MENA and Central A │        18          0          0 │       240 
8. Sub-Saharan Africa │         3          1          0 │        24 
9. South and South-Ea │         1          0          0 │        26 
       10. East Asian │         3          0          0 │         9 
   11. Latin American │         7          0          0 │        29 
        12. Caribbean │         0          1          0 │         1 
──────────────────────┼─────────────────────────────────┼──────────
                Total │     4,965        101         13 │     5,829 


    country of birth, │
 classified in ESCEG, │
     of respondent or │          Ethnic groups
     mother or father │      SEur      SEEur       EEur │     Total
──────────────────────┼─────────────────────────────────┼──────────
0. no migration backg │        20          6         46 │     4,692 
     1. West European │         3          2          2 │       122 
    2. North European │         0          0          0 │        13 
    3. South European │        69          0          0 │        77 
4. South-East Europea │         0         83          3 │       124 
     5. East European │         0          2        183 │       451 
6. North American and │         0          1          0 │        21 
7. MENA and Central A │         0          0         26 │       240 
8. Sub-Saharan Africa │         0          0          0 │        24 
9. South and South-Ea │         0          0          0 │        26 
       10. East Asian │         0          0          0 │         9 
   11. Latin American │         1          0          0 │        29 
        12. Caribbean │         0          0          0 │         1 
──────────────────────┼─────────────────────────────────┼──────────
                Total │        93         94        260 │     5,829 


    country of birth, │
 classified in ESCEG, │
     of respondent or │          Ethnic groups
     mother or father │    NAmAus     MENACA     SubSAf │     Total
──────────────────────┼─────────────────────────────────┼──────────
0. no migration backg │         6          4          0 │     4,692 
     1. West European │         0          1          0 │       122 
    2. North European │         0          0          0 │        13 
    3. South European │         1          0          0 │        77 
4. South-East Europea │         2          0          0 │       124 
     5. East European │         2          2          0 │       451 
6. North American and │        11          0          0 │        21 
7. MENA and Central A │         1        194          0 │       240 
8. Sub-Saharan Africa │         0          0         18 │        24 
9. South and South-Ea │         0          0          0 │        26 
       10. East Asian │         0          0          0 │         9 
   11. Latin American │         0          0          0 │        29 
        12. Caribbean │         0          0          0 │         1 
──────────────────────┼─────────────────────────────────┼──────────
                Total │        23        201         18 │     5,829 


    country of birth, │
 classified in ESCEG, │
     of respondent or │          Ethnic groups
     mother or father │      SSEA         EA        LAm │     Total
──────────────────────┼─────────────────────────────────┼──────────
0. no migration backg │         1          1          2 │     4,692 
     1. West European │         0          0          1 │       122 
    2. North European │         0          0          0 │        13 
    3. South European │         0          0          0 │        77 
4. South-East Europea │         0          0          0 │       124 
     5. East European │         0          0          0 │       451 
6. North American and │         0          0          0 │        21 
7. MENA and Central A │         1          0          0 │       240 
8. Sub-Saharan Africa │         1          0          1 │        24 
9. South and South-Ea │        25          0          0 │        26 
       10. East Asian │         0          6          0 │         9 
   11. Latin American │         2          0         19 │        29 
        12. Caribbean │         0          0          0 │         1 
──────────────────────┼─────────────────────────────────┼──────────
                Total │        30          7         23 │     5,829 


    country of birth, │
 classified in ESCEG, │   Ethnic
     of respondent or │   groups
     mother or father │       Car │     Total
──────────────────────┼───────────┼──────────
0. no migration backg │         1 │     4,692 
     1. West European │         0 │       122 
    2. North European │         0 │        13 
    3. South European │         0 │        77 
4. South-East Europea │         0 │       124 
     5. East European │         0 │       451 
6. North American and │         0 │        21 
7. MENA and Central A │         0 │       240 
8. Sub-Saharan Africa │         0 │        24 
9. South and South-Ea │         0 │        26 
       10. East Asian │         0 │         9 
   11. Latin American │         0 │        29 
        12. Caribbean │         0 │         1 
──────────────────────┼───────────┼──────────
                Total │         1 │     5,829
```

A rather large number (225) of people in Germany report an Eastern European country of birth, either for themselves or one or both of their parents, while claiming German ancestry only, out of a total of 411 with a Eastern European country of birth. At a long distance, this is followed by 42 (out of 122) respondents with a Western European country of birth, and 24 (out of 103) with a South-East European country of birth but German ancestry only. 18 say one person was born in MENA/Central Asia without any non-German ancestry, out of all 240 with a MENA/Central Asian country of birth (and here we also a remarkable number of 26 respondents claiming East European ancestry). So while for the MENA/Central Asian group, country of birth and ancestry largely agree, especially for migrants from Eastern Europe, a large fraction appears to be of German descent. Note that so-called ‘late emigrants’, i.e. people of German descent “returning” from countries of the former Warsaw Pact (as well as Yugoslavia) have the right to obtain German citizenship right away. **Here (like maybe also with the ‘pieds noirs’), I wonder whether ancestry is the best indicator to study integration because the ’re-’integration of returning migrants is not obvious at all. In the German case, this often even meant that no knowledge of German was present any more since the Soviet Union strongly enforced usage of Russian (while the French in Algeria always spoke French).**

```
. tab rcob anctryall2r if cntry=="de" & rcob!="66"

Country of │                Ethnic groups
     birth │   Autocht       WEur       NEur       SEur │     Total
───────────┼────────────────────────────────────────────┼──────────
        02 │         0          0          0          0 │         3 
        03 │         7          0          0          0 │         7 
        04 │         1          0          0          0 │         4 
        06 │         0          0          0          0 │         1 
        77 │         1          0          0          0 │         1 
        99 │         0          0          0          0 │         1 
        AF │         1          0          0          0 │        10 
        AL │         0          0          0          0 │         2 
        AM │         0          0          0          0 │         4 
        AR │         0          0          0          0 │         2 
        AT │         5          8          0          1 │        14 
        AU │         0          0          0          0 │         2 
        AZ │         0          0          0          0 │         1 
        BA │         0          0          0          0 │         9 
        BD │         0          0          0          0 │         1 
        BE │         0          2          0          0 │         2 
        BG │         0          0          0          0 │         5 
        BR │         0          0          0          0 │         5 
        BY │         0          0          0          0 │         2 
        CA │         0          0          0          0 │         1 
        CD │         0          0          0          0 │         1 
        CG │         0          0          0          0 │         1 
        CH │         1          2          0          1 │         4 
        CN │         1          0          0          0 │         4 
        CO │         0          0          0          0 │         1 
        CU │         0          0          0          0 │         3 
        CZ │         8          0          0          0 │        15 
        DK │         1          0          2          0 │         3 
        DZ │         1          0          0          0 │         4 
        EE │         0          0          0          0 │         1 
        ER │         0          0          0          0 │         1 
        ES │         0          0          0          9 │         9 
        FR │         1          5          0          0 │         8 
        GB │         0          4          0          1 │         6 
        GM │         0          0          0          0 │         1 
        GR │         0          0          0          7 │         8 
        GT │         0          0          0          0 │         1 
        HR │         0          0          0          0 │        11 
        HU │         2          0          0          0 │         7 
        IL │         0          0          0          0 │         1 
        IN │         0          0          0          0 │         1 
        IQ │         0          0          0          0 │         8 
        IR │         0          0          0          0 │        11 
        IT │         0          0          0         11 │        11 
        JO │         1          0          0          0 │         1 
        JP │         0          0          0          0 │         2 
        KE │         0          0          0          0 │         1 
        KG │         1          0          0          0 │         4 
        KW │         0          0          0          0 │         1 
        KZ │         6          0          0          0 │        36 
        LB │         1          0          0          0 │         2 
        LK │         0          0          0          0 │         1 
        LT │         0          0          0          0 │         4 
        LV │         0          0          0          0 │         1 
        MA │         0          0          0          0 │         4 
        MD │         1          0          0          0 │         1 
        MK │         0          0          0          0 │         3 
        MX │         1          0          0          0 │         2 
        NG │         0          0          0          0 │         4 
        NL │         0         15          0          0 │        15 
        PH │         0          0          0          0 │         1 
        PK │         0          0          0          0 │         4 
        PL │        27          0          0          0 │        95 
        PS │         0          0          0          0 │         1 
        PT │         0          0          0          4 │         4 
        RO │         8          0          0          0 │        26 
        RS │         0          0          0          0 │         5 
        RU │         6          0          0          0 │        42 
        SA │         0          0          0          0 │         1 
        SE │         0          0          1          0 │         1 
        SI │         0          0          0          0 │         3 
        SK │         0          0          0          0 │         3 
        SO │         0          0          0          0 │         2 
        SV │         0          0          0          0 │         1 
        SY │         1          0          0          0 │        19 
        TD │         0          1          0          0 │         1 
        TG │         0          0          0          0 │         1 
        TM │         0          0          0          0 │         1 
        TN │         0          0          0          0 │         3 
        TR │         0          0          0          0 │        49 
        TZ │         0          0          0          0 │         1 
        UA │         2          0          0          0 │        20 
        US │         1          0          0          0 │         5 
        UZ │         1          0          0          0 │         5 
        VN │         0          0          0          0 │         6 
        XK │         0          0          0          0 │         9 
        ZA │         0          0          0          0 │         2 
        ZM │         0          0          0          0 │         2 
───────────┼────────────────────────────────────────────┼──────────
     Total │        87         37          3         34 │       593 


Country of │                Ethnic groups
     birth │     SEEur       EEur     NAmAus     MENACA │     Total
───────────┼────────────────────────────────────────────┼──────────
        02 │         0          3          0          0 │         3 
        03 │         0          0          0          0 │         7 
        04 │         3          0          0          0 │         4 
        06 │         1          0          0          0 │         1 
        77 │         0          0          0          0 │         1 
        99 │         0          0          0          1 │         1 
        AF │         0          0          0          9 │        10 
        AL │         2          0          0          0 │         2 
        AM │         0          0          0          4 │         4 
        AR │         0          0          0          0 │         2 
        AT │         0          0          0          0 │        14 
        AU │         1          0          1          0 │         2 
        AZ │         0          1          0          0 │         1 
        BA │         9          0          0          0 │         9 
        BD │         0          0          0          0 │         1 
        BE │         0          0          0          0 │         2 
        BG │         5          0          0          0 │         5 
        BR │         0          0          0          0 │         5 
        BY │         0          2          0          0 │         2 
        CA │         0          0          1          0 │         1 
        CD │         0          0          0          0 │         1 
        CG │         0          0          0          0 │         1 
        CH │         0          0          0          0 │         4 
        CN │         0          0          0          0 │         4 
        CO │         0          0          0          0 │         1 
        CU │         0          0          0          0 │         3 
        CZ │         0          7          0          0 │        15 
        DK │         0          0          0          0 │         3 
        DZ │         0          0          0          3 │         4 
        EE │         0          1          0          0 │         1 
        ER │         0          0          0          0 │         1 
        ES │         0          0          0          0 │         9 
        FR │         1          0          0          1 │         8 
        GB │         0          0          0          0 │         6 
        GM │         0          0          0          0 │         1 
        GR │         0          0          1          0 │         8 
        GT │         0          0          0          0 │         1 
        HR │        10          1          0          0 │        11 
        HU │         0          5          0          0 │         7 
        IL │         0          0          0          1 │         1 
        IN │         0          0          0          0 │         1 
        IQ │         0          0          0          8 │         8 
        IR │         0          0          0         11 │        11 
        IT │         0          0          0          0 │        11 
        JO │         0          0          0          0 │         1 
        JP │         0          0          0          0 │         2 
        KE │         0          0          0          0 │         1 
        KG │         0          1          0          2 │         4 
        KW │         0          0          0          1 │         1 
        KZ │         0         20          1          9 │        36 
        LB │         0          0          0          1 │         2 
        LK │         0          0          0          0 │         1 
        LT │         0          4          0          0 │         4 
        LV │         0          1          0          0 │         1 
        MA │         0          0          0          4 │         4 
        MD │         0          0          0          0 │         1 
        MK │         2          0          1          0 │         3 
        MX │         0          0          0          0 │         2 
        NG │         0          0          0          0 │         4 
        NL │         0          0          0          0 │        15 
        PH │         0          0          0          0 │         1 
        PK │         0          0          0          0 │         4 
        PL │         0         68          0          0 │        95 
        PS │         0          0          0          1 │         1 
        PT │         0          0          0          0 │         4 
        RO │        17          1          0          0 │        26 
        RS │         4          1          0          0 │         5 
        RU │         0         35          0          1 │        42 
        SA │         0          0          0          1 │         1 
        SE │         0          0          0          0 │         1 
        SI │         3          0          0          0 │         3 
        SK │         0          3          0          0 │         3 
        SO │         0          0          0          0 │         2 
        SV │         0          0          0          0 │         1 
        SY │         0          0          0         18 │        19 
        TD │         0          0          0          0 │         1 
        TG │         0          0          0          0 │         1 
        TM │         0          0          0          1 │         1 
        TN │         0          0          0          3 │         3 
        TR │         0          0          0         49 │        49 
        TZ │         0          0          0          0 │         1 
        UA │         0         18          0          0 │        20 
        US │         0          0          4          0 │         5 
        UZ │         0          3          0          1 │         5 
        VN │         0          0          0          0 │         6 
        XK │         8          0          0          1 │         9 
        ZA │         0          0          0          0 │         2 
        ZM │         0          0          0          0 │         2 
───────────┼────────────────────────────────────────────┼──────────
     Total │        66        175          9        131 │       593 


Country of │                Ethnic groups
     birth │    SubSAf       SSEA         EA        LAm │     Total
───────────┼────────────────────────────────────────────┼──────────
        02 │         0          0          0          0 │         3 
        03 │         0          0          0          0 │         7 
        04 │         0          0          0          0 │         4 
        06 │         0          0          0          0 │         1 
        77 │         0          0          0          0 │         1 
        99 │         0          0          0          0 │         1 
        AF │         0          0          0          0 │        10 
        AL │         0          0          0          0 │         2 
        AM │         0          0          0          0 │         4 
        AR │         0          0          0          2 │         2 
        AT │         0          0          0          0 │        14 
        AU │         0          0          0          0 │         2 
        AZ │         0          0          0          0 │         1 
        BA │         0          0          0          0 │         9 
        BD │         0          1          0          0 │         1 
        BE │         0          0          0          0 │         2 
        BG │         0          0          0          0 │         5 
        BR │         0          0          0          5 │         5 
        BY │         0          0          0          0 │         2 
        CA │         0          0          0          0 │         1 
        CD │         1          0          0          0 │         1 
        CG │         1          0          0          0 │         1 
        CH │         0          0          0          0 │         4 
        CN │         0          0          3          0 │         4 
        CO │         0          0          0          1 │         1 
        CU │         0          0          0          3 │         3 
        CZ │         0          0          0          0 │        15 
        DK │         0          0          0          0 │         3 
        DZ │         0          0          0          0 │         4 
        EE │         0          0          0          0 │         1 
        ER │         1          0          0          0 │         1 
        ES │         0          0          0          0 │         9 
        FR │         0          0          0          0 │         8 
        GB │         0          0          0          1 │         6 
        GM │         1          0          0          0 │         1 
        GR │         0          0          0          0 │         8 
        GT │         0          0          0          1 │         1 
        HR │         0          0          0          0 │        11 
        HU │         0          0          0          0 │         7 
        IL │         0          0          0          0 │         1 
        IN │         0          1          0          0 │         1 
        IQ │         0          0          0          0 │         8 
        IR │         0          0          0          0 │        11 
        IT │         0          0          0          0 │        11 
        JO │         0          0          0          0 │         1 
        JP │         0          0          2          0 │         2 
        KE │         1          0          0          0 │         1 
        KG │         0          0          0          0 │         4 
        KW │         0          0          0          0 │         1 
        KZ │         0          0          0          0 │        36 
        LB │         0          0          0          0 │         2 
        LK │         0          1          0          0 │         1 
        LT │         0          0          0          0 │         4 
        LV │         0          0          0          0 │         1 
        MA │         0          0          0          0 │         4 
        MD │         0          0          0          0 │         1 
        MK │         0          0          0          0 │         3 
        MX │         0          0          0          1 │         2 
        NG │         4          0          0          0 │         4 
        NL │         0          0          0          0 │        15 
        PH │         0          1          0          0 │         1 
        PK │         0          4          0          0 │         4 
        PL │         0          0          0          0 │        95 
        PS │         0          0          0          0 │         1 
        PT │         0          0          0          0 │         4 
        RO │         0          0          0          0 │        26 
        RS │         0          0          0          0 │         5 
        RU │         0          0          0          0 │        42 
        SA │         0          0          0          0 │         1 
        SE │         0          0          0          0 │         1 
        SI │         0          0          0          0 │         3 
        SK │         0          0          0          0 │         3 
        SO │         2          0          0          0 │         2 
        SV │         0          0          0          1 │         1 
        SY │         0          0          0          0 │        19 
        TD │         0          0          0          0 │         1 
        TG │         1          0          0          0 │         1 
        TM │         0          0          0          0 │         1 
        TN │         0          0          0          0 │         3 
        TR │         0          0          0          0 │        49 
        TZ │         1          0          0          0 │         1 
        UA │         0          0          0          0 │        20 
        US │         0          0          0          0 │         5 
        UZ │         0          0          0          0 │         5 
        VN │         0          6          0          0 │         6 
        XK │         0          0          0          0 │         9 
        ZA │         1          0          0          1 │         2 
        ZM │         2          0          0          0 │         2 
───────────┼────────────────────────────────────────────┼──────────
     Total │        16         14          5         16 │       593
```

Looking at respondents’ country of birth, the patterns look very plausible for respondents born in Spain, Italy, Greece, Turkey, France, the UK, with almost all *not* reporting German ancestry and reflecting mostly labour-market related migration. Looking at recent and mostly refugee origins, out of the 10 respondents born in Afghanistan and the 19 born in Syria, one claims exclusively German ancestry in each case (born in 1987 and 1994 respectively). This looks rather unlikely, and there may be language issues involved since these are usually not native speakers of German. A detailed look reveals that they are both recent young migrants who arrived in Germany in 2012 and 2015 respectively, supporting the suspicion that this may indeed be measurement error on the ancestry variable.

list age livecnta rcob mcob fcob anctry1 anctry2 if (rcob==“AF”|rcob==“SY”) & cntry==“de” & anctryall2r==0

All respondents born in Iraq (8) or Iran (11) report MENA ancestry only. Looking further back, people born in former Yugoslavia (BA, HR, RS, SI, XK), who fled to Germany in large numbers in the 1990s, do not report German ancestry either. These are thus all simple cases where we don’t find any indications of measurement issues.

27 of the 95 born in Poland say they have exclusively German ancestry. Here we expect to find individuals of German ancestry who were expelled from Eastern Europe in its new boundaries in the aftermath of WWII, and indeed 10 of these respondents report to have ‘moved’ to Germany between 1944 and 1949. Even though the question on country of birth in the German questionnaire has a longish note as to what should be considered as “Germany”, namely all territories that belonged to Germany when the respondent was born, it seems like a number of respondents did not understand or ignored the instruction and reported e.g. Poland as their country of birth because their birthplace today belongs to Poland, even though it was part of Germany when they were born. This then leads to measurement error on the country of birth and migration background variables.

With respect to the countries of origin of the German diaspora in Eastern Europe and Romania (i.e. German-speaking people in territories that were never part of Germany or only in occupation during WWII, thus forming ethnic minorities in these countries), 6 out of the 42 born in Russia, 6 out of 36 born in Kazakhstan, 8 out of 26 born in Romania, 2 out of 20 born in Ukraine, 8 out of 15 born in the Czech Republic, 2 out of 7 born in Hungary, and 1 out of 5 born in Uzbekistan say they have German ancestry only. (Note that a substantial German minority lived in Western Czechia before WWII, and the respective area was usurped by Hitler in 1938 and its inhabitants were also expelled after WWII.) I would have expected higher proportions of people not reporting East European ancestry here. Maybe this partly is due to the ambiguous translation of the ancestry item in German where the term ‘Herkunft’ (origin) was used, which can be understood to refer to geographic rather than ethnic origin. Also, there is migration of ethnic Eastern Europeans to Germany since 1990 as well. **Here again I could do extra checks using year of birth and year of migration if needed.**

14 respondents were born in Austria, out of which 5 claim German ancestry only. This is plausible given Austria is German-speaking and there is some common labor market between these two countries.

```
. tab mcob anctryall2r if cntry=="de" & mcob!="66"

Country of │
    birth, │                Ethnic groups
    mother │   Autocht       WEur       NEur       SEur │     Total
───────────┼────────────────────────────────────────────┼──────────
        02 │         3          0          0          0 │        12 
        03 │        21          0          0          0 │        24 
        04 │         7          0          0          0 │        15 
        88 │         1          0          0          1 │         3 
        99 │         0          0          0          0 │         1 
        AF │         1          0          0          0 │         9 
        AL │         0          0          0          0 │         2 
        AM │         0          0          0          0 │         4 
        AR │         1          0          0          0 │         3 
        AT │        17         14          0          1 │        33 
        AU │         1          0          0          0 │         3 
        AZ │         0          0          0          0 │         1 
        BA │         0          0          0          0 │        13 
        BD │         0          0          0          0 │         1 
        BE │         2          3          0          0 │         5 
        BG │         0          0          0          0 │         5 
        BR │         2          0          0          0 │         7 
        BY │         0          0          0          0 │         2 
        CD │         0          0          0          0 │         1 
        CG │         0          0          0          0 │         1 
        CH │         3          3          0          0 │         6 
        CN │         0          0          0          0 │         4 
        CO │         0          0          0          0 │         1 
        CR │         0          0          0          0 │         1 
        CU │         0          0          0          0 │         3 
        CZ │        30          0          0          0 │        36 
        DK │         0          0          2          0 │         2 
        DZ │         1          0          0          0 │         4 
        EE │         1          0          0          0 │         2 
        ER │         0          0          0          0 │         1 
        ES │         0          0          0         16 │        16 
        FI │         1          0          1          0 │         2 
        FR │         2          6          0          0 │         9 
        GB │         0          7          0          0 │         7 
        GH │         0          0          0          0 │         1 
        GM │         0          0          0          0 │         1 
        GR │         1          0          0         10 │        13 
        GT │         0          0          0          0 │         2 
        HR │         0          0          0          0 │        18 
        HU │        12          0          0          0 │        18 
        ID │         0          0          0          0 │         1 
        IL │         0          0          0          0 │         2 
        IN │         0          0          0          0 │         2 
        IQ │         0          0          0          0 │         8 
        IR │         0          0          0          0 │        12 
        IT │         0          0          0         19 │        19 
        JM │         0          1          0          0 │         1 
        JO │         1          0          0          0 │         1 
        JP │         1          0          0          0 │         2 
        KE │         0          0          0          0 │         1 
        KG │         0          0          0          0 │         4 
        KW │         0          0          0          0 │         1 
        KZ │         3          0          0          0 │        22 
        LB │         1          0          0          0 │         6 
        LK │         0          0          0          0 │         2 
        LT │         0          0          0          0 │         3 
        LV │         3          0          0          0 │         3 
        MA │         0          0          0          0 │         5 
        MD │         1          0          0          0 │         2 
        MK │         0          0          0          0 │         3 
        MX │         0          0          0          0 │         1 
        MZ │         0          0          0          1 │         1 
        NG │         0          0          0          0 │         2 
        NL │         0         18          0          0 │        18 
        NO │         1          0          0          0 │         1 
        NP │         0          0          0          0 │         1 
        PH │         1          0          0          0 │         4 
        PK │         0          0          0          0 │         5 
        PL │        62          1          0          0 │       138 
        PS │         0          0          0          0 │         4 
        PT │         0          0          0          3 │         3 
        RO │        12          0          0          0 │        37 
        RS │         1          1          0          0 │         9 
        RU │        15          0          0          0 │        60 
        SE │         0          0          2          0 │         2 
        SI │         3          0          0          0 │         4 
        SK │         0          0          0          0 │         2 
        SO │         0          0          0          0 │         1 
        SV │         0          0          0          0 │         1 
        SY │         1          0          0          0 │        20 
        TD │         0          1          0          0 │         1 
        TG │         0          0          0          0 │         1 
        TM │         0          0          0          0 │         1 
        TN │         0          0          0          0 │         4 
        TR │         0          0          0          0 │        93 
        UA │         6          0          0          0 │        26 
        US │         2          0          0          0 │         4 
        UZ │         1          0          0          0 │         4 
        VN │         0          0          0          0 │        10 
        XK │         0          0          0          0 │        13 
        ZA │         0          0          0          0 │         2 
        ZM │         0          0          0          0 │         2 
───────────┼────────────────────────────────────────────┼──────────
     Total │       222         55          5         51 │       867 


Country of │
    birth, │                Ethnic groups
    mother │     SEEur       EEur     NAmAus     MENACA │     Total
───────────┼────────────────────────────────────────────┼──────────
        02 │         0          9          0          0 │        12 
        03 │         0          3          0          0 │        24 
        04 │         7          0          1          0 │        15 
        88 │         0          0          0          1 │         3 
        99 │         1          0          0          0 │         1 
        AF │         0          0          0          7 │         9 
        AL │         2          0          0          0 │         2 
        AM │         0          0          0          4 │         4 
        AR │         0          0          0          0 │         3 
        AT │         0          1          0          0 │        33 
        AU │         0          0          2          0 │         3 
        AZ │         0          1          0          0 │         1 
        BA │        13          0          0          0 │        13 
        BD │         0          0          0          0 │         1 
        BE │         0          0          0          0 │         5 
        BG │         5          0          0          0 │         5 
        BR │         0          0          0          0 │         7 
        BY │         0          2          0          0 │         2 
        CD │         0          0          0          0 │         1 
        CG │         0          0          0          0 │         1 
        CH │         0          0          0          0 │         6 
        CN │         0          0          0          0 │         4 
        CO │         0          0          0          0 │         1 
        CR │         0          0          0          0 │         1 
        CU │         0          0          0          0 │         3 
        CZ │         0          6          0          0 │        36 
        DK │         0          0          0          0 │         2 
        DZ │         0          0          0          3 │         4 
        EE │         0          1          0          0 │         2 
        ER │         0          0          0          0 │         1 
        ES │         0          0          0          0 │        16 
        FI │         0          0          0          0 │         2 
        FR │         1          0          0          0 │         9 
        GB │         0          0          0          0 │         7 
        GH │         0          0          0          0 │         1 
        GM │         0          0          0          0 │         1 
        GR │         0          1          1          0 │        13 
        GT │         0          0          1          0 │         2 
        HR │        17          1          0          0 │        18 
        HU │         0          6          0          0 │        18 
        ID │         0          0          0          0 │         1 
        IL │         0          0          0          2 │         2 
        IN │         0          0          0          0 │         2 
        IQ │         0          0          0          8 │         8 
        IR │         0          0          0         12 │        12 
        IT │         0          0          0          0 │        19 
        JM │         0          0          0          0 │         1 
        JO │         0          0          0          0 │         1 
        JP │         0          0          0          0 │         2 
        KE │         0          0          0          0 │         1 
        KG │         0          3          0          1 │         4 
        KW │         0          0          0          1 │         1 
        KZ │         0         11          1          7 │        22 
        LB │         0          0          0          5 │         6 
        LK │         0          0          0          0 │         2 
        LT │         0          3          0          0 │         3 
        LV │         0          0          0          0 │         3 
        MA │         0          0          0          5 │         5 
        MD │         0          1          0          0 │         2 
        MK │         2          0          1          0 │         3 
        MX │         0          0          0          0 │         1 
        MZ │         0          0          0          0 │         1 
        NG │         0          0          0          0 │         2 
        NL │         0          0          0          0 │        18 
        NO │         0          0          0          0 │         1 
        NP │         0          0          0          0 │         1 
        PH │         0          0          0          0 │         4 
        PK │         0          0          0          0 │         5 
        PL │         0         73          1          1 │       138 
        PS │         0          0          0          4 │         4 
        PT │         0          0          0          0 │         3 
        RO │        23          1          1          0 │        37 
        RS │         6          1          0          0 │         9 
        RU │         0         39          0          6 │        60 
        SE │         0          0          0          0 │         2 
        SI │         1          0          0          0 │         4 
        SK │         0          2          0          0 │         2 
        SO │         0          0          0          0 │         1 
        SV │         0          0          0          0 │         1 
        SY │         0          0          0         19 │        20 
        TD │         0          0          0          0 │         1 
        TG │         0          0          0          0 │         1 
        TM │         0          0          0          1 │         1 
        TN │         0          0          0          4 │         4 
        TR │         0          0          0         93 │        93 
        UA │         0         20          0          0 │        26 
        US │         0          0          2          0 │         4 
        UZ │         0          2          0          1 │         4 
        VN │         0          0          0          0 │        10 
        XK │        12          0          0          1 │        13 
        ZA │         0          0          0          0 │         2 
        ZM │         0          0          0          0 │         2 
───────────┼────────────────────────────────────────────┼──────────
     Total │        90        187         11        186 │       867 


Country of │
    birth, │                Ethnic groups
    mother │    SubSAf       SSEA         EA        LAm │     Total
───────────┼────────────────────────────────────────────┼──────────
        02 │         0          0          0          0 │        12 
        03 │         0          0          0          0 │        24 
        04 │         0          0          0          0 │        15 
        88 │         0          0          0          0 │         3 
        99 │         0          0          0          0 │         1 
        AF │         0          1          0          0 │         9 
        AL │         0          0          0          0 │         2 
        AM │         0          0          0          0 │         4 
        AR │         0          0          0          2 │         3 
        AT │         0          0          0          0 │        33 
        AU │         0          0          0          0 │         3 
        AZ │         0          0          0          0 │         1 
        BA │         0          0          0          0 │        13 
        BD │         0          1          0          0 │         1 
        BE │         0          0          0          0 │         5 
        BG │         0          0          0          0 │         5 
        BR │         0          0          0          5 │         7 
        BY │         0          0          0          0 │         2 
        CD │         1          0          0          0 │         1 
        CG │         1          0          0          0 │         1 
        CH │         0          0          0          0 │         6 
        CN │         0          0          4          0 │         4 
        CO │         0          0          0          1 │         1 
        CR │         0          0          0          1 │         1 
        CU │         0          0          0          3 │         3 
        CZ │         0          0          0          0 │        36 
        DK │         0          0          0          0 │         2 
        DZ │         0          0          0          0 │         4 
        EE │         0          0          0          0 │         2 
        ER │         1          0          0          0 │         1 
        ES │         0          0          0          0 │        16 
        FI │         0          0          0          0 │         2 
        FR │         0          0          0          0 │         9 
        GB │         0          0          0          0 │         7 
        GH │         1          0          0          0 │         1 
        GM │         1          0          0          0 │         1 
        GR │         0          0          0          0 │        13 
        GT │         0          0          0          1 │         2 
        HR │         0          0          0          0 │        18 
        HU │         0          0          0          0 │        18 
        ID │         0          1          0          0 │         1 
        IL │         0          0          0          0 │         2 
        IN │         0          2          0          0 │         2 
        IQ │         0          0          0          0 │         8 
        IR │         0          0          0          0 │        12 
        IT │         0          0          0          0 │        19 
        JM │         0          0          0          0 │         1 
        JO │         0          0          0          0 │         1 
        JP │         0          0          1          0 │         2 
        KE │         1          0          0          0 │         1 
        KG │         0          0          0          0 │         4 
        KW │         0          0          0          0 │         1 
        KZ │         0          0          0          0 │        22 
        LB │         0          0          0          0 │         6 
        LK │         0          2          0          0 │         2 
        LT │         0          0          0          0 │         3 
        LV │         0          0          0          0 │         3 
        MA │         0          0          0          0 │         5 
        MD │         0          0          0          0 │         2 
        MK │         0          0          0          0 │         3 
        MX │         0          0          0          1 │         1 
        MZ │         0          0          0          0 │         1 
        NG │         2          0          0          0 │         2 
        NL │         0          0          0          0 │        18 
        NO │         0          0          0          0 │         1 
        NP │         0          1          0          0 │         1 
        PH │         0          2          0          1 │         4 
        PK │         0          5          0          0 │         5 
        PL │         0          0          0          0 │       138 
        PS │         0          0          0          0 │         4 
        PT │         0          0          0          0 │         3 
        RO │         0          0          0          0 │        37 
        RS │         0          0          0          0 │         9 
        RU │         0          0          0          0 │        60 
        SE │         0          0          0          0 │         2 
        SI │         0          0          0          0 │         4 
        SK │         0          0          0          0 │         2 
        SO │         1          0          0          0 │         1 
        SV │         0          0          0          1 │         1 
        SY │         0          0          0          0 │        20 
        TD │         0          0          0          0 │         1 
        TG │         1          0          0          0 │         1 
        TM │         0          0          0          0 │         1 
        TN │         0          0          0          0 │         4 
        TR │         0          0          0          0 │        93 
        UA │         0          0          0          0 │        26 
        US │         0          0          0          0 │         4 
        UZ │         0          0          0          0 │         4 
        VN │         0         10          0          0 │        10 
        XK │         0          0          0          0 │        13 
        ZA │         1          1          0          0 │         2 
        ZM │         2          0          0          0 │         2 
───────────┼────────────────────────────────────────────┼──────────
     Total │        13         26          5         16 │       867 

. tab fcob anctryall2r if cntry=="de" & fcob!="66"

Country of │
    birth, │                Ethnic groups
    father │   Autocht       WEur       NEur       SEur │     Total
───────────┼────────────────────────────────────────────┼──────────
        02 │         1          0          0          0 │         8 
        03 │        25          0          0          0 │        28 
        04 │        10          0          0          0 │        22 
        06 │         0          0          0          0 │         1 
        77 │         1          0          0          0 │         1 
        88 │         5          0          0          0 │         5 
        99 │         2          0          0          0 │         4 
        AF │         1          0          0          0 │        11 
        AL │         0          0          0          0 │         3 
        AM │         0          0          0          0 │         4 
        AO │         1          0          0          0 │         1 
        AT │        22         18          0          0 │        41 
        AU │         0          0          0          0 │         1 
        AZ │         0          0          0          0 │         1 
        BA │         0          0          0          0 │        14 
        BD │         1          0          0          0 │         2 
        BE │         1          2          0          0 │         4 
        BG │         0          0          0          0 │         5 
        BR │         1          0          0          0 │         6 
        BY │         1          0          0          1 │         3 
        CD │         0          0          0          0 │         1 
        CG │         0          0          0          0 │         1 
        CH │         1          3          0          0 │         4 
        CL │         1          0          0          0 │         2 
        CN │         0          0          0          0 │         5 
        CO │         0          0          0          0 │         1 
        CU │         0          0          0          1 │         4 
        CZ │        36          0          0          0 │        44 
        DE │         1          0          0          0 │         1 
        DK │         3          0          1          0 │         4 
        DZ │         2          0          0          0 │         5 
        EE │         1          0          0          0 │         2 
        EG │         1          0          0          0 │         2 
        ER │         0          0          0          0 │         1 
        ES │         1          0          0         15 │        16 
        FI │         1          0          0          0 │         1 
        FR │         1          7          0          0 │        10 
        GB │         0          7          0          0 │         7 
        GE │         0          0          0          0 │         1 
        GM │         0          0          0          0 │         1 
        GR │         3          0          0         12 │        16 
        GT │         0          0          0          0 │         1 
        HR │         1          0          0          0 │        18 
        HU │        10          0          0          0 │        17 
        ID │         0          1          0          0 │         2 
        IL │         0          0          0          0 │         2 
        IN │         1          0          0          0 │         2 
        IQ │         0          0          0          0 │         8 
        IR │         0          0          0          0 │        11 
        IT │         3          0          0         32 │        35 
        JO │         1          0          0          0 │         1 
        JP │         1          0          0          0 │         3 
        KE │         0          0          0          0 │         1 
        KG │         0          0          0          0 │         3 
        KW │         0          0          0          0 │         1 
        KZ │         3          0          0          0 │        23 
        LB │         1          0          0          0 │         5 
        LK │         0          0          0          0 │         2 
        LT │         1          0          0          0 │         4 
        LV │         2          0          0          0 │         2 
        MA │         0          0          0          0 │         4 
        MD │         1          0          0          0 │         1 
        MK │         0          0          0          0 │         3 
        MO │         0          0          0          0 │         2 
        MX │         0          0          0          0 │         1 
        MZ │         0          0          0          0 │         1 
        NA │         1          0          0          0 │         1 
        NG │         1          0          0          0 │         6 
        NL │         2         17          0          0 │        19 
        PH │         0          0          0          0 │         3 
        PK │         0          0          0          0 │         7 
        PL │        71          2          0          0 │       143 
        PS │         1          0          0          0 │         5 
        PT │         0          0          0          5 │         5 
        RO │        13          0          0          0 │        37 
        RS │         1          0          0          0 │         5 
        RU │        14          0          0          0 │        61 
        SE │         0          0          2          0 │         2 
        SI │         0          0          0          0 │         1 
        SK │         1          0          0          0 │         3 
        SO │         0          0          0          0 │         2 
        SV │         0          0          0          0 │         1 
        SY │         1          0          0          0 │        21 
        TD │         0          1          0          0 │         1 
        TG │         0          0          0          0 │         1 
        TM │         0          0          0          0 │         2 
        TN │         0          0          0          0 │         4 
        TR │         1          0          0          1 │       105 
        TZ │         0          0          0          0 │         1 
        UA │        14          0          0          0 │        37 
        US │         6          0          0          0 │        15 
        UY │         0          0          0          0 │         1 
        UZ │         0          0          0          0 │         1 
        VN │         0          0          0          0 │        10 
        XK │         0          0          0          0 │        13 
        ZA │         0          0          0          0 │         1 
        ZM │         0          0          0          0 │         2 
───────────┼────────────────────────────────────────────┼──────────
     Total │       276         58          3         67 │       963 


Country of │
    birth, │                Ethnic groups
    father │     SEEur       EEur     NAmAus     MENACA │     Total
───────────┼────────────────────────────────────────────┼──────────
        02 │         0          7          0          0 │         8 
        03 │         1          2          0          0 │        28 
        04 │        10          0          1          1 │        22 
        06 │         1          0          0          0 │         1 
        77 │         0          0          0          0 │         1 
        88 │         0          0          0          0 │         5 
        99 │         2          0          0          0 │         4 
        AF │         0          0          0          9 │        11 
        AL │         3          0          0          0 │         3 
        AM │         0          0          0          4 │         4 
        AO │         0          0          0          0 │         1 
        AT │         0          1          0          0 │        41 
        AU │         0          0          1          0 │         1 
        AZ │         0          1          0          0 │         1 
        BA │        14          0          0          0 │        14 
        BD │         0          0          0          0 │         2 
        BE │         0          1          0          0 │         4 
        BG │         5          0          0          0 │         5 
        BR │         0          0          1          0 │         6 
        BY │         0          1          0          0 │         3 
        CD │         0          0          0          0 │         1 
        CG │         0          0          0          0 │         1 
        CH │         0          0          0          0 │         4 
        CL │         0          0          0          0 │         2 
        CN │         0          0          1          0 │         5 
        CO │         0          0          0          0 │         1 
        CU │         0          0          0          0 │         4 
        CZ │         0          8          0          0 │        44 
        DE │         0          0          0          0 │         1 
        DK │         0          0          0          0 │         4 
        DZ │         0          0          0          3 │         5 
        EE │         0          1          0          0 │         2 
        EG │         0          0          0          1 │         2 
        ER │         0          0          0          0 │         1 
        ES │         0          0          0          0 │        16 
        FI │         0          0          0          0 │         1 
        FR │         0          2          0          0 │        10 
        GB │         0          0          0          0 │         7 
        GE │         0          0          0          1 │         1 
        GM │         0          0          0          0 │         1 
        GR │         0          0          1          0 │        16 
        GT │         0          0          0          0 │         1 
        HR │        16          1          0          0 │        18 
        HU │         1          6          0          0 │        17 
        ID │         0          0          0          0 │         2 
        IL │         0          0          0          2 │         2 
        IN │         0          0          0          0 │         2 
        IQ │         0          0          0          8 │         8 
        IR │         0          0          0         11 │        11 
        IT │         0          0          0          0 │        35 
        JO │         0          0          0          0 │         1 
        JP │         0          0          0          0 │         3 
        KE │         0          0          0          0 │         1 
        KG │         0          2          0          1 │         3 
        KW │         0          0          0          1 │         1 
        KZ │         0         12          0          8 │        23 
        LB │         0          0          0          4 │         5 
        LK │         0          0          0          0 │         2 
        LT │         0          3          0          0 │         4 
        LV │         0          0          0          0 │         2 
        MA │         0          0          0          4 │         4 
        MD │         0          0          0          0 │         1 
        MK │         2          0          1          0 │         3 
        MO │         0          0          0          2 │         2 
        MX │         0          0          0          0 │         1 
        MZ │         0          0          0          0 │         1 
        NA │         0          0          0          0 │         1 
        NG │         0          0          0          0 │         6 
        NL │         0          0          0          0 │        19 
        PH │         0          0          0          0 │         3 
        PK │         0          0          0          0 │         7 
        PL │         1         68          1          0 │       143 
        PS │         0          0          0          4 │         5 
        PT │         0          0          0          0 │         5 
        RO │        22          2          0          0 │        37 
        RS │         3          1          0          0 │         5 
        RU │         0         41          1          5 │        61 
        SE │         0          0          0          0 │         2 
        SI │         1          0          0          0 │         1 
        SK │         0          2          0          0 │         3 
        SO │         0          0          0          0 │         2 
        SV │         0          0          0          0 │         1 
        SY │         0          0          0         20 │        21 
        TD │         0          0          0          0 │         1 
        TG │         0          0          0          0 │         1 
        TM │         0          1          0          1 │         2 
        TN │         0          0          0          4 │         4 
        TR │         0          0          0        103 │       105 
        TZ │         0          0          0          0 │         1 
        UA │         0         23          0          0 │        37 
        US │         0          0          8          1 │        15 
        UY │         0          0          0          0 │         1 
        UZ │         0          1          0          0 │         1 
        VN │         0          0          0          0 │        10 
        XK │        12          0          0          1 │        13 
        ZA │         0          0          0          0 │         1 
        ZM │         0          0          0          0 │         2 
───────────┼────────────────────────────────────────────┼──────────
     Total │        94        187         16        199 │       963 


Country of │
    birth, │                Ethnic groups
    father │    SubSAf       SSEA         EA        LAm │     Total
───────────┼────────────────────────────────────────────┼──────────
        02 │         0          0          0          0 │         8 
        03 │         0          0          0          0 │        28 
        04 │         0          0          0          0 │        22 
        06 │         0          0          0          0 │         1 
        77 │         0          0          0          0 │         1 
        88 │         0          0          0          0 │         5 
        99 │         0          0          0          0 │         4 
        AF │         0          1          0          0 │        11 
        AL │         0          0          0          0 │         3 
        AM │         0          0          0          0 │         4 
        AO │         0          0          0          0 │         1 
        AT │         0          0          0          0 │        41 
        AU │         0          0          0          0 │         1 
        AZ │         0          0          0          0 │         1 
        BA │         0          0          0          0 │        14 
        BD │         0          1          0          0 │         2 
        BE │         0          0          0          0 │         4 
        BG │         0          0          0          0 │         5 
        BR │         0          0          0          4 │         6 
        BY │         0          0          0          0 │         3 
        CD │         1          0          0          0 │         1 
        CG │         1          0          0          0 │         1 
        CH │         0          0          0          0 │         4 
        CL │         0          0          0          1 │         2 
        CN │         0          0          4          0 │         5 
        CO │         0          0          0          1 │         1 
        CU │         0          0          0          3 │         4 
        CZ │         0          0          0          0 │        44 
        DE │         0          0          0          0 │         1 
        DK │         0          0          0          0 │         4 
        DZ │         0          0          0          0 │         5 
        EE │         0          0          0          0 │         2 
        EG │         0          0          0          0 │         2 
        ER │         1          0          0          0 │         1 
        ES │         0          0          0          0 │        16 
        FI │         0          0          0          0 │         1 
        FR │         0          0          0          0 │        10 
        GB │         0          0          0          0 │         7 
        GE │         0          0          0          0 │         1 
        GM │         1          0          0          0 │         1 
        GR │         0          0          0          0 │        16 
        GT │         0          0          0          1 │         1 
        HR │         0          0          0          0 │        18 
        HU │         0          0          0          0 │        17 
        ID │         0          1          0          0 │         2 
        IL │         0          0          0          0 │         2 
        IN │         0          1          0          0 │         2 
        IQ │         0          0          0          0 │         8 
        IR │         0          0          0          0 │        11 
        IT │         0          0          0          0 │        35 
        JO │         0          0          0          0 │         1 
        JP │         0          0          2          0 │         3 
        KE │         1          0          0          0 │         1 
        KG │         0          0          0          0 │         3 
        KW │         0          0          0          0 │         1 
        KZ │         0          0          0          0 │        23 
        LB │         0          0          0          0 │         5 
        LK │         0          2          0          0 │         2 
        LT │         0          0          0          0 │         4 
        LV │         0          0          0          0 │         2 
        MA │         0          0          0          0 │         4 
        MD │         0          0          0          0 │         1 
        MK │         0          0          0          0 │         3 
        MO │         0          0          0          0 │         2 
        MX │         0          0          0          1 │         1 
        MZ │         1          0          0          0 │         1 
        NA │         0          0          0          0 │         1 
        NG │         5          0          0          0 │         6 
        NL │         0          0          0          0 │        19 
        PH │         0          2          0          1 │         3 
        PK │         0          7          0          0 │         7 
        PL │         0          0          0          0 │       143 
        PS │         0          0          0          0 │         5 
        PT │         0          0          0          0 │         5 
        RO │         0          0          0          0 │        37 
        RS │         0          0          0          0 │         5 
        RU │         0          0          0          0 │        61 
        SE │         0          0          0          0 │         2 
        SI │         0          0          0          0 │         1 
        SK │         0          0          0          0 │         3 
        SO │         2          0          0          0 │         2 
        SV │         0          0          0          1 │         1 
        SY │         0          0          0          0 │        21 
        TD │         0          0          0          0 │         1 
        TG │         1          0          0          0 │         1 
        TM │         0          0          0          0 │         2 
        TN │         0          0          0          0 │         4 
        TR │         0          0          0          0 │       105 
        TZ │         1          0          0          0 │         1 
        UA │         0          0          0          0 │        37 
        US │         0          0          0          0 │        15 
        UY │         0          0          0          1 │         1 
        UZ │         0          0          0          0 │         1 
        VN │         0         10          0          0 │        10 
        XK │         0          0          0          0 │        13 
        ZA │         1          0          0          0 │         1 
        ZM │         2          0          0          0 │         2 
───────────┼────────────────────────────────────────────┼──────────
     Total │        18         25          6         14 │       963
```

The results concerning mothers’ and fathers’ country of birth are in line with what was found for respondents, just that for example more mothers and fathers were born in Hungary and the respective respondents report exclusively German ancestry, which is again highly plausible.

# Summary and Conclusions

Looking at the relationship between socio-cultural origins based on measures of ancestry vs. measures of country of birth, a surprisingly high number of respondents does not report a foreign ancestry despite being born abroad (or mother or father being born abroad). A closer inspection of the specific countries of origin and ancestry-based origin in the UK, France and Germany does not reveal any apparent measurement problems though. In contrast, the results differentiate quite clearly between different countries of birth in relation to historic migration movements between origin and the destination (survey) country, reflecting 1. ‘simple’ migration from origin to destination country without previous migration between the affected countries, resulting in consistent measures of country of birth and ancestry, 2. migration along established links between origin and destination country, where the destination country had substantial numbers of country nationals living in the origin country for sometimes many generations, resulting in large numbers where ancestry agrees with the survey (destination) country, but the country of birth (origin) is different. This is e.g. commonly the case when an origin country was a colony of the destination country, or destination country nationals had settled a foreign country and established a ‘diaspora’ there without ruling that country, e.g. because they fled from religious or political prosecution or overpopulation. In both cases, a ‘return’ to the homeland is more likely than migration elsewhere if living conditions deteriorate. The measures even work so well that we can see combinations of variables that look unlikely without any knowledge on the history of migration of the affected countries, but that are totally plausible once this knowledge is available to aid interpretation, such as respondents of South European ancestry in France who were born in Algeria.

In conclusion, both measures in combination tell a richer story of countries’ diversity in terms of migration old and new than either measure by itself. While the country of birth-based measure often suggests a migration background when there was migration involved but the destination country is the actual ‘cultural home’ of the respondent probably leading to rather smooth integration, the ancestry measure does not reveal whether somebody (or their parents) with majority ancestry has migrated or not (and more often than expected, they did).

So for modeling integration, it is probably best to include both measures of ancestry as well as migration background, where migration background only needs to reflect generational status while the ancestry-based measure picks up the effect of non-autochthonous origins.
