# Supplementary material for: Dimensions of Migrant Integration in Western Europe
Source: Front Sociol. 2021 Apr 29;6:510987. doi: 10.3389/fsoc.2021.510987 (PMC8116888; doi:10.3389/fsoc.2021.510987)
Supplement: Supplementary file 1 [file DataSheet1.zip › Appendix C.pdf]

Appendix C: Figures comparing models with ancestry and country-of-birth (COB) based measures of ethnic group

Note that with measures of ethnic group based on COB, the third generation cannot be identified since the country of birth of grandparents was not asked. Third generation respondents with grandparents born abroad will thus be included in the majority group without migration background. Also, the majority is expected to be homogeneous with respect to generation since only people without migration background are considered as belonging to the majority group. Returnees (first- and second-generation migrants with autochthonous background) do thus also not exist in this measure of ethnic group. So the main difference in results between using one or the other measure is that when using the measure based on ancestry, we have richer results that also cover the 3rd+ generation with ethnic minority background as well as the 1st and 2nd generation with autochthonous background.

Table 1: Crosstabs of country of birth and ancestry in ESCEG by migrant generation

COB	1ST GEN	2ND GEN	MAJ	TOTAL	ANCESTRY	1ST GEN	2ND GEN	3RD GEN+	TOTAL
**NO MIG BG**	0	0	25,735	25,735	**Autocht**	298	1,537	24,371	26,206
**WEUR**	758	1,042	0	1,800	**WEur**	674	474	657	1,805
**NEUR**	21	21	0	42	**NEur**	24	12	9	45
**SEUR**	304	515	0	819	**SEur**	353	452	334	1,139
**SEEUR**	357	243	0	600	**SEEur**	390	196	32	618
**EEUR**	608	425	0	1,033	**EEur**	599	129	107	835
**NAMAUS**	52	86	0	138	**NAmAus**	71	42	18	131
**MENACA**	580	540	0	1,120	**MENACA**	488	425	40	953
**SUBSAF**	269	114	0	383	**SubSAf**	202	39	10	251
**SSEA**	229	200	0	429	**SSEA**	243	156	33	432
**EA**	34	21	0	55	**EA**	50	20	8	78
**LAM**	143	61	0	204	**LAm**	116	38	7	161
**CAR**	63	61	0	124	**Car**	61	68	53	182
**TOTAL**	3,418	3,329	25,735	32,482	**Total**	3,569	3,588	25,679	32,836

Figure 1: Adjusted predictions at representative values for structural integration indicators with 95% confidence intervals (main effects models) using both ancestry and country of birth-based measures

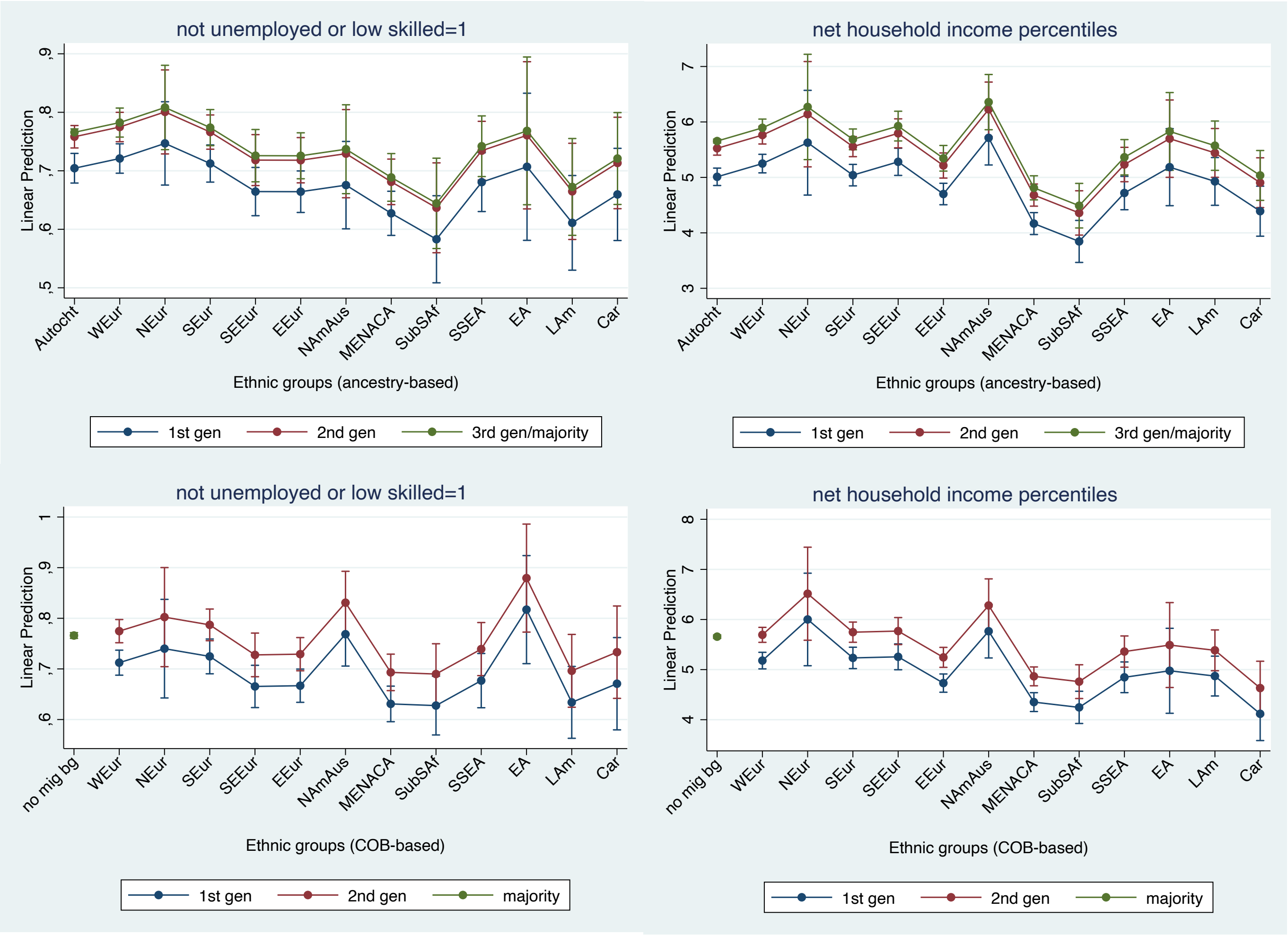

Figure 2: Adjusted predictions at representative values for cultural integration indicators with 95% confidence intervals using both ancestry and country of birth-based measures

a) non-homophobic attitude (main effects model)

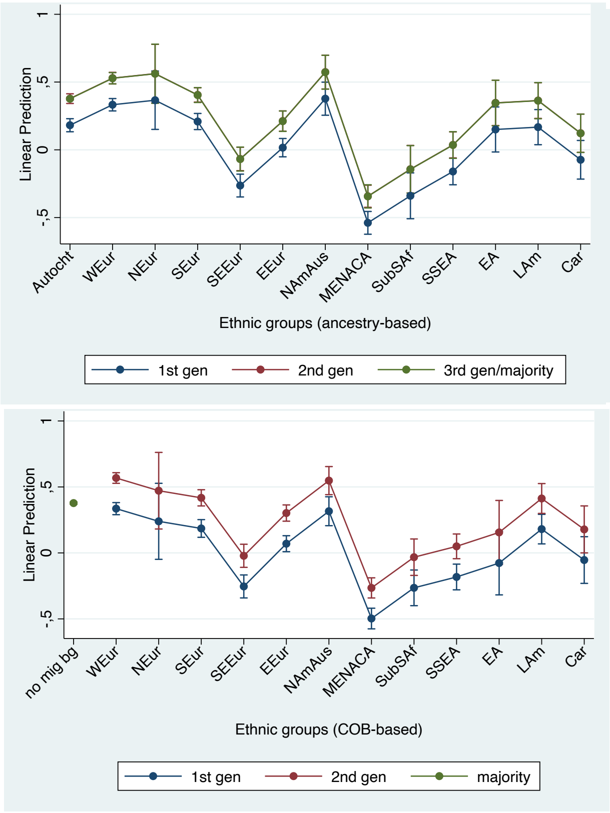

b) gender equality (main effects model)

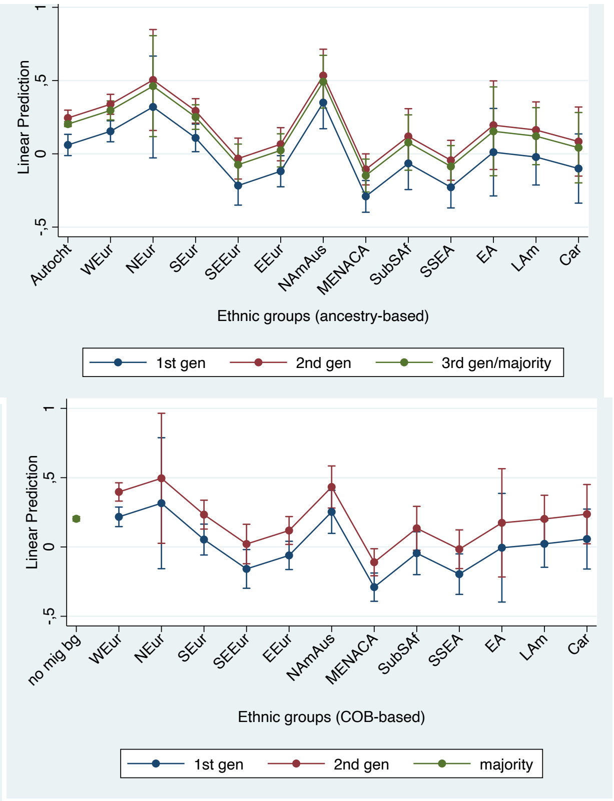

c) non-homophobic attitude (interaction effects)

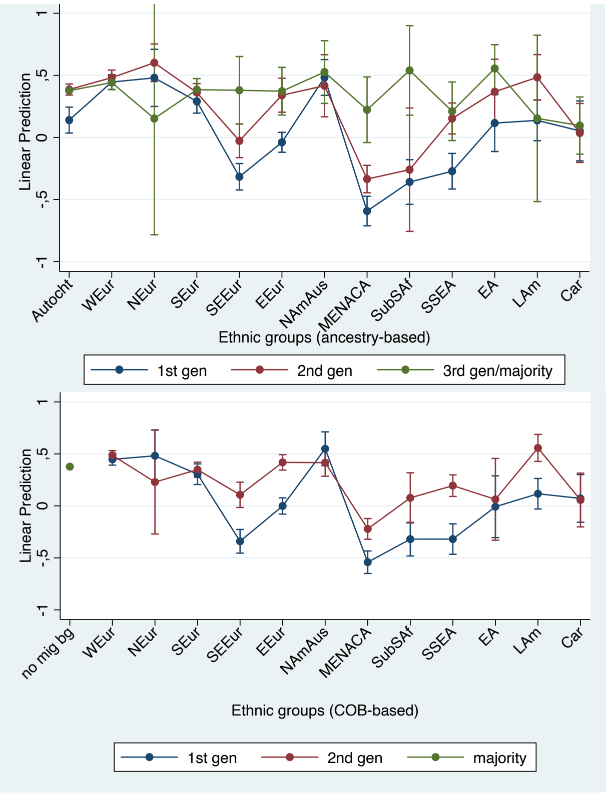

Figure 3: Adjusted predictions at representative values for social integration indicators with 95% confidence intervals (main effects models) using both ancestry and country of birth-based measures

a) no or some minority ethnic minority people in living area

b) no ethnic minority friends

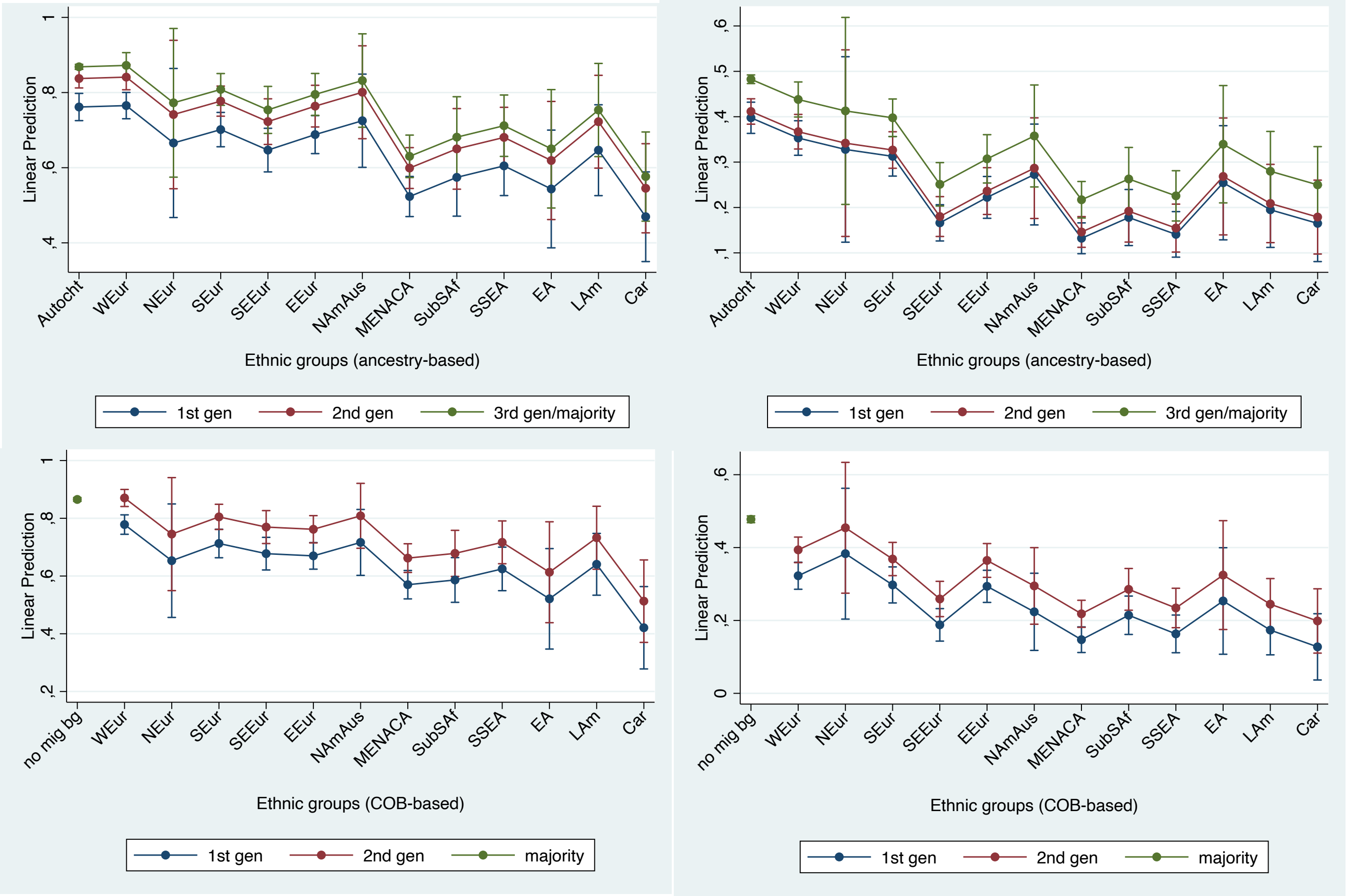

Figure 4: Adjusted predictions at representative values for political integration indicators with 95% confidence intervals (main effects models) using both ancestry and country of birth-based measures

a) voting

b) non-electoral participation

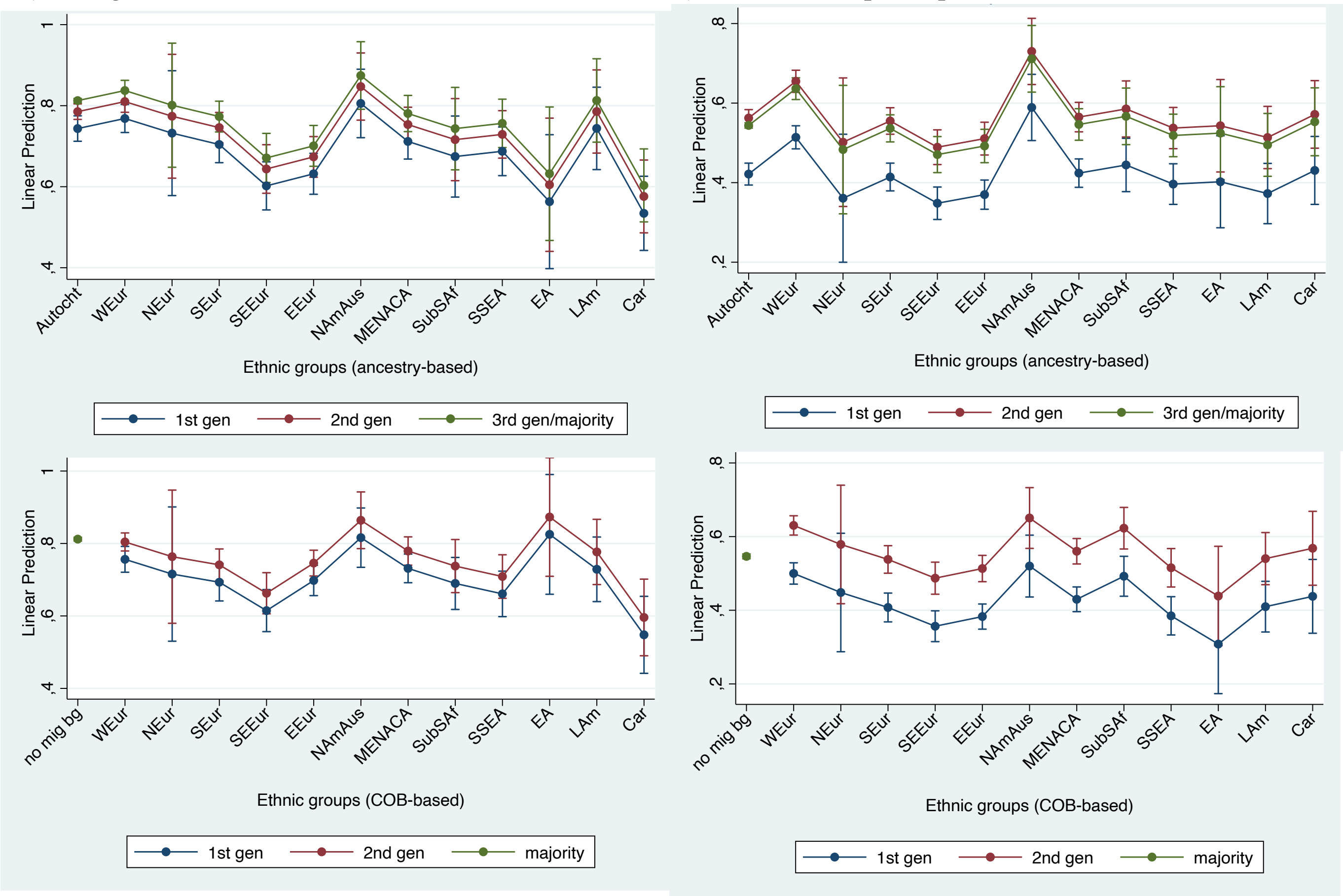

Figure 5: Adjusted predictions at representative values for civic integration indicators with 95% confidence intervals using both ancestry and country of birth-based measures

a) citizenship (main effects model)

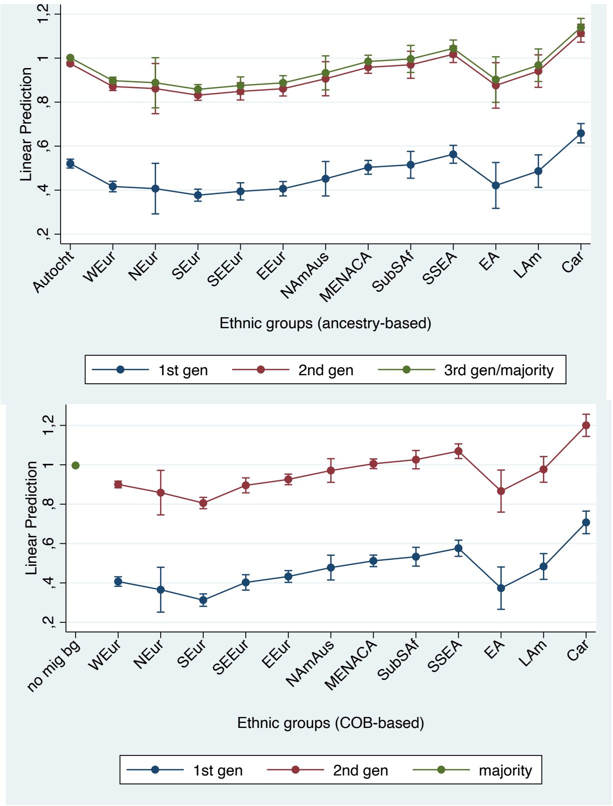

b) national attachment (main effects model)

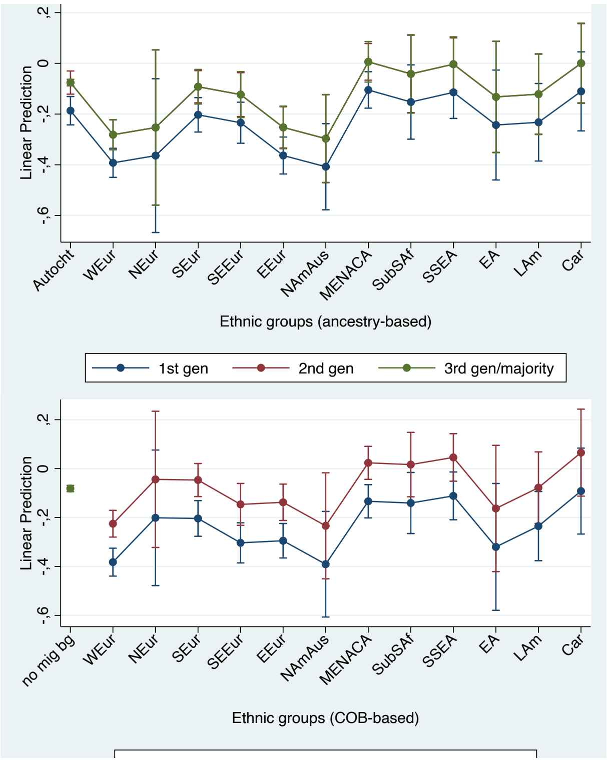

e) citizenship (interaction effects model)

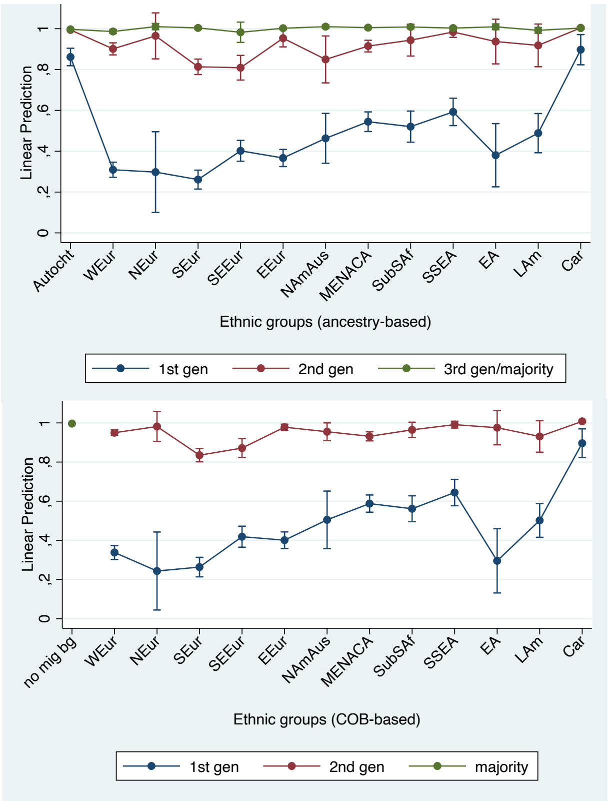

f) citizenship (interaction effects model)
